# Hotspots of human impact on threatened terrestrial vertebrates

**DOI:** 10.1371/journal.pbio.3000158

**Published:** 2019-03-12

**Authors:** James R. Allan, James E. M. Watson, Moreno Di Marco, Christopher J. O’Bryan, Hugh P. Possingham, Scott C. Atkinson, Oscar Venter

**Affiliations:** 1 School of Earth and Environmental Sciences, The University of Queensland, Brisbane, Australia; 2 Centre for Biodiversity and Conservation Science, The University of Queensland, Brisbane, Australia; 3 Wildlife Conservation Society, Global Conservation Program, New York, New York, United States of America; 4 CSIRO Land & Water, EcoSciences Precinct, Brisbane, Australia; 5 The Nature Conservancy, Arlington, Virginia, United States of America; 6 United Nations Development Programme (UNDP), New York, New York, United States of America; 7 Natural Resource and Environmental Studies Institute, University of Northern British Columbia, Prince George, Canada; Estacion Biologica de Doñana CSIC, SPAIN

## Abstract

Conserving threatened species requires identifying where across their range they are being impacted by threats, yet this remains unresolved across most of Earth. Here, we present a global analysis of cumulative human impacts on threatened species by using a spatial framework that jointly considers the co-occurrence of eight threatening processes and the distribution of 5,457 terrestrial vertebrates. We show that impacts to species are widespread, occurring across 84% of Earth’s surface, and identify hotspots of impacted species richness and coolspots of unimpacted species richness. Almost one-quarter of assessed species are impacted across >90% of their distribution, and approximately 7% are impacted across their entire range. These results foreshadow localised extirpations and potential extinctions without conservation action. The spatial framework developed here offers a tool for defining strategies to directly mitigate the threats driving species’ declines, providing essential information for future national and global conservation agendas.

## Abstract 2

A global analysis of human impacts on threatened vertebrates shows that they are almost ubiquitous across Earth, and hundreds of species have no refuge from these impacts. The study also identifies hotspots of impacted species richness and coolspots of unimpacted species richness.

## Author summary

The biggest drivers of global biodiversity loss are hunting, harvesting, and the conversion of natural habitats for agriculture, urbanisation, and other industrial activity. However, our understanding of where these ‘threats’ actually impact sensitive species is extremely limited across Earth. Here, we map the distribution of threats within the known ranges of 5,457 terrestrial birds, mammals, and amphibians globally. We map only those threats within a species range that are known to specifically endanger that species. We show that threats are extensive across the majority of species’ ranges, severely limiting the area within which species can survive. Concerningly, 1,237 species (almost one-quarter of those assessed) are impacted by threats across >90% of their distribution, and 395 species are impacted by threats across their entire range. These species will almost certainly face extinction without conservation intervention to remove threats. We identify global hotspots of impacted species richness and also ‘coolspots’ that act as refuges from threats, providing essential information for conservation planning and action.

## Introduction

Human activities and land usage are exerting unprecedented pressure on natural environments [1,2], threatening to drive tens of thousands of species to extinction [3]. The main drivers of species declines include the conversion of natural habitats for land usage such as crops, pasture, and infrastructure, as well as the overexploitation of species through activities such as hunting [3,4]. The distribution of these activities varies across Earth’s terrestrial surface [1], as do the distributions of the species they threaten [5]. Understanding and quantifying spatial patterns of where human pressures overlap with sensitive species (i.e., mapping human impacts to threatened species) will improve our ability to prioritise actions to manage and mitigate human impacts on biodiversity [6,7]. Importantly, it will allow for the identification of areas across species distributions that are free from those threats that the species is sensitive to, and this information can be used to map global ‘coolspots’ of what we call ‘threat refugia’. Both forms of information are essential for conservation planning and can guide action towards securing these impact-free refugia, which are paramount for the survival of many threatened species [8–11].

Mapping impacts to biodiversity requires linking spatial data on the distributions of threats with the distributions of species known to be sensitive to those threats [12]. To date, no efforts undertaken at either regional [13,14] or global extents [1,15–18] have accounted for the distribution and sensitivity of species and their threats and therefore do not directly map likely human impacts [19]. Past efforts that simply map threats [1] fail to account for the distribution of species that respond to those threats, and even overlapping threats with species ranges [20] does not account for the specific sensitivities of each species to co-occurring threats. Some efforts to map threats to the marine realm estimated their impacts at the coarse ecosystem scale but did not account for individual species sensitivities [12,21]. The few studies that do account for species have either been conducted at fine spatial resolutions [22] or consider a limited number of taxonomic groups [23,24], and many suffer from the assumption that species are exposed to threats across their entire range, not just where the threat occurs, overestimating impacts [20,25,26]. Clearly, our understanding of where individual species are being impacted by threats or where their threat-free refugia are remains limited at the global scale [27] and is a major gap in our ability to prioritise conservation actions [27,28].

**Fig 1:**
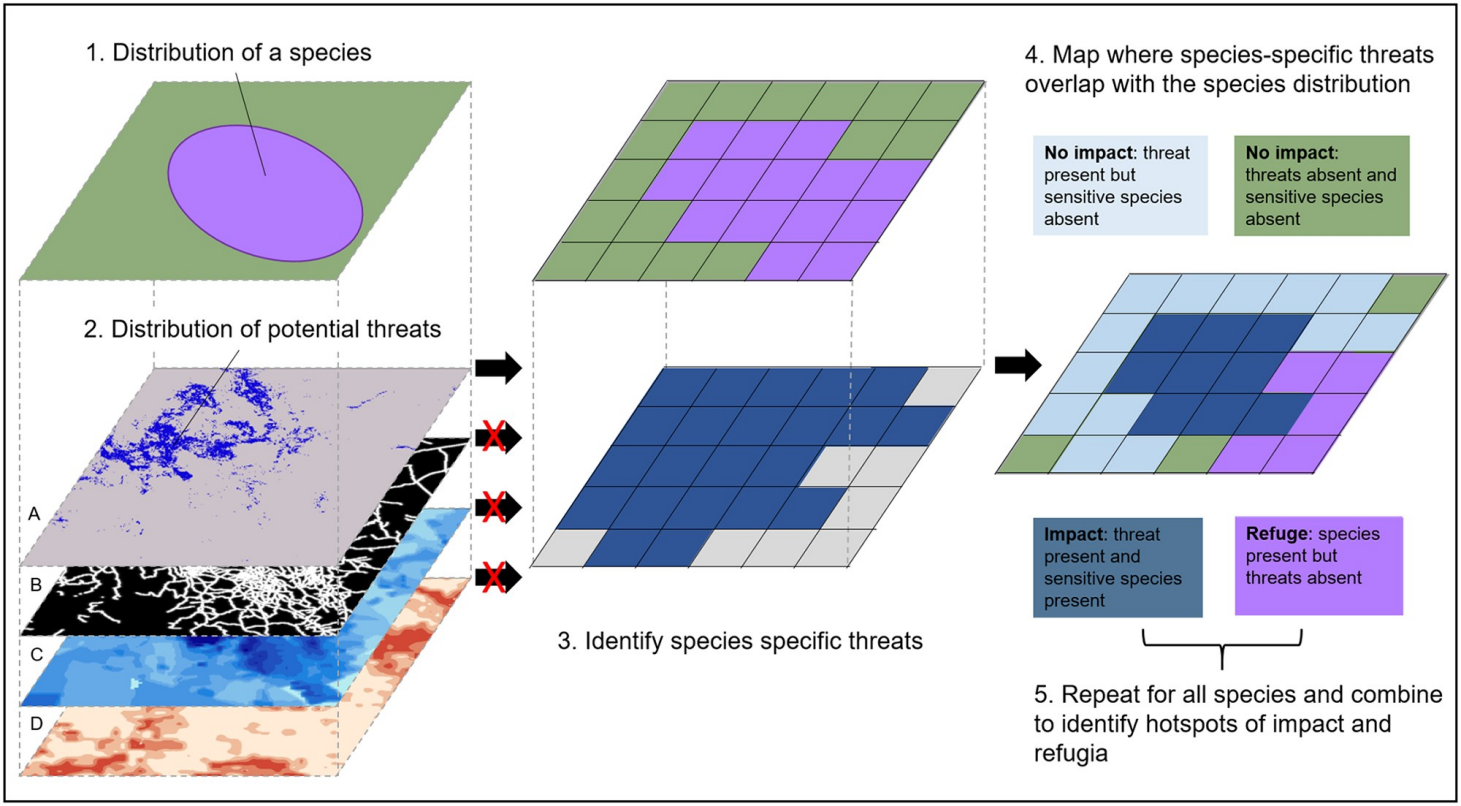
Methodological framework for mapping cumulative human impacts on threatened vertebrate species.

Here, we present the first global assessment of the spatial distribution of human impacts on globally threatened and near-threatened terrestrial birds, mammals, and amphibians. We developed a novel, to our knowledge, method for quantifying and mapping human impacts that jointly considers the distributions of 5,457 threatened and near-threatened species (1,277 mammals, 2,120 birds, and 2,060 amphibians), the distribution of species-specific threats, and the extent to which the distribution of each species is impacted by relevant threats (Fig 1).

Spatial data on threats were obtained from the recently updated Human Footprint [1], which is unique for considering eight human pressures globally at a 1 km^2^ resolution, including built environments, crop lands, pasture lands, human population density, night lights, railways, major roadways, and navigable waterways. This makes the Human Footprint the most complete and highest resolution globally consistent dataset of anthropogenic threats [29]. Each individual pressure was linked to a species if they directly or indirectly correspond to threats identified by the International Union for Conservation of Nature (IUCN) Red List [30] as driving the endangerment of that species. The Human Footprint data correspond with seven major classes and 15 subclasses of IUCN threats (Table 1; S1 Table). Although these do not include all threats to species, they do include all of the most prevalent drivers of global biodiversity decline [4]. We calculated the proportion of each species range that is currently impacted by a threat and then mapped cumulative human impacts in a 30 km × 30 km grid globally (see Materials and methods). We also examined patterns of human impacts across individual species distributions, taxonomic groups, and threat status categories. Finally, we used the inverse of our cumulative impact maps to identify threat refugia, the places where high numbers of threatened (and near-threatened) species persist unimpacted by human activity.

**Table 1:** Major classes and subclasses of threats to biodiversity, as classified in the IUCN Red List of Threatened Species, and the corresponding spatially explicit pressure variable from the updated Human Footprint dataset.

Major threat class (IUCN)	Subclass threats (IUCN)	Pressure (Human Footprint)	Species Impacted
1. Residential and commercial development	1.1 Housing and urban areas	Electric infrastructure (nightlights)	1,748
		Built environments	
	1.2 Commercial and industrial areas	Electric infrastructure (nightlights)	349
		Built environments	
2. Agriculture and aquaculture	2.1 Annual and perennial nontimber crops	Crop lands	4,017
	2.3 Livestock farming and ranching	Pasture lands	1,850
4. Transportation and service corridors	4.1 Roads and railroads	Railways	563
		Roads	
	4.2 Utility and service lines	Roads	88
5. Biological resource use	5.1 Hunting and collecting terrestrial animals	Navigable waterways	1,594
		Population density	
		Roads	
	5.2 Gathering terrestrial plants	Navigable waterways	149
		Population density	
		Roads	
6. Human intrusions and disturbance	6.1 Recreational activities*	Electric infrastructure (nightlights)	
		Population density	373
	6.3 Work and other activities	Electric infrastructure (nightlights)	196
		Population density	
8. Invasive and other problematic species, genes, and diseases	8.1 Invasive non-native/alien species/diseases	Population density	1,319
		Roads	
9. Pollution	9.1 Domestic and urban waste water	Population density	205
		Built environments	
	9.3 Agriculture and forestry effluents	Crop lands	805
	9.4 Garbage and solid waste	Built environments	27
	9.6 Excess energy	Electric infrastructure (nightlights)	24
		Built environments	

*We excluded navigable waterways because these pressures are generally limited to aquatic-dwelling species by the IUCN, and our analysis focuses on terrestrial species. Additionally, we excluded roads because the pressures described by the IUCN for this category are generally limited to dirt roads, which are not represented in the Human Footprint. **Abbreviation**: IUCN, International Union for Conservation of Nature.

## Results

### Human impacts on threatened vertebrate species

**Fig 2:**
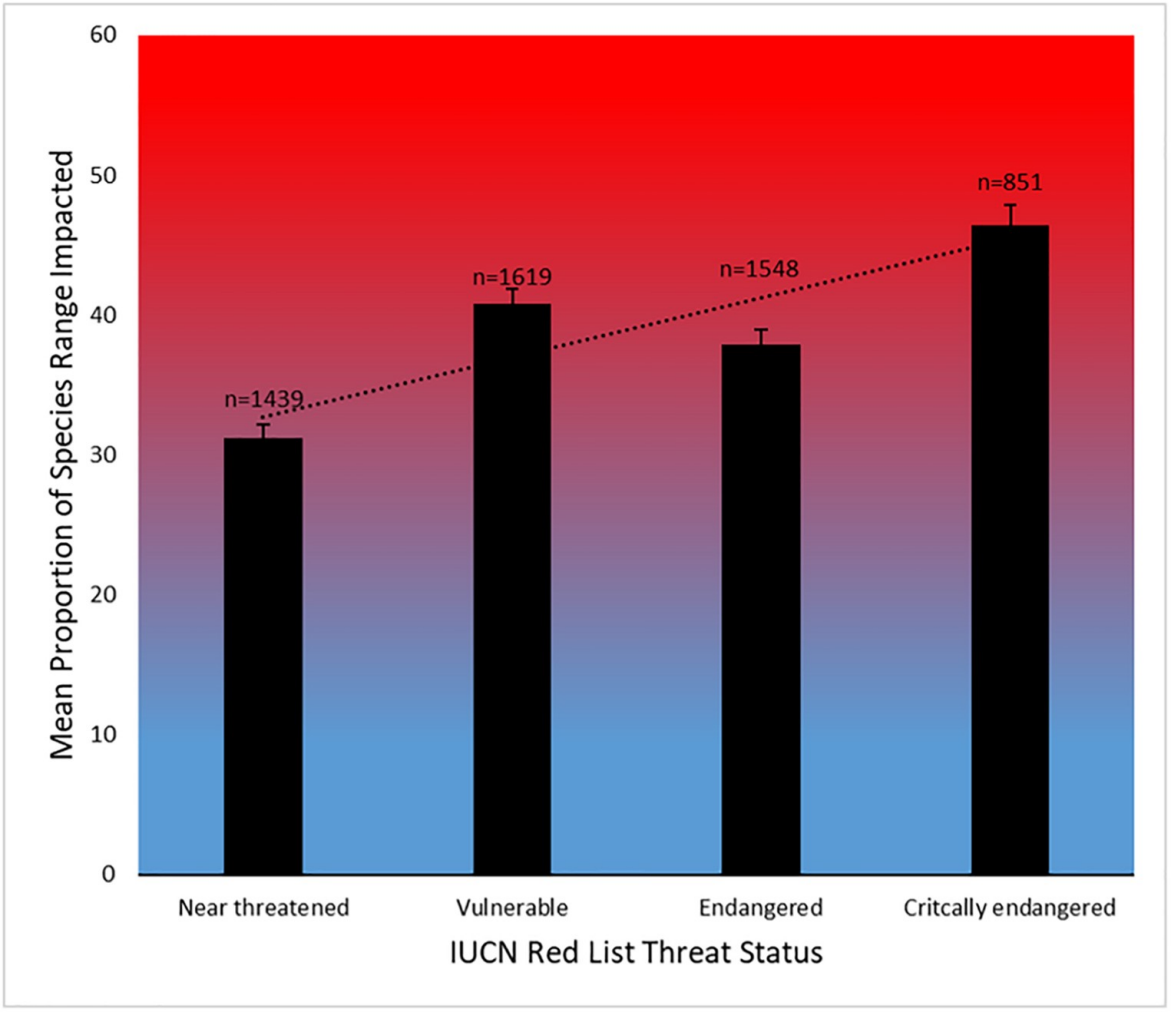
Mean proportion of species distributions impacted by threats across extinction risk categories of threatened and near-threatened terrestrial vertebrates. Bars represent means with standard errors. The data underlying this figure are freely available [31] (doi:10.1594/PANGAEA.897391). Species extinction risk assessed by the IUCN (2015). IUCN, International Union for Conservation of Nature.

We found that on average, 38% of a species’ distribution range is impacted by one or more relevant threats (Table 2, S1 Data), including an average 21% of the distribution impacted by multiple co-occurring threats. Mammals are the most impacted of all taxa, with on average 52% of a species’ distribution impacted by relevant threats. Concerningly, almost one-quarter of all species (23%, *n* = 1,237) are impacted by threats across >90% of their distribution, with 395 (7%) impacted by at least one relevant threat across their entire distribution. Conversely, we found that one-third of all species (34%, *n* = 1,863) are not exposed to the threats we mapped across any portion of their distribution; however, this result should be interpreted within the context of threats we consider. We also found that the proportion of a species distribution impacted by threats correlates with its threat status (IUCN Red List categories; Fig 2) (analysis of variance *P* < 0.001, F = 7.5). Species classified as critically endangered on the IUCN Red List had almost half their distribution impacted by threats on average (46%, *n* = 851), whilst near-threatened species had one-third of their distribution impacted by threats on average (31%, *n* = 1,439).

**Table 2:** The number and percentage of species and the proportion of their distribution impacted by threats.

		Proportion of range impacted by threats					
	Total number of species	0%	1%–50%	50%–90%	90%–99%	100%	Mean (%)
**Amphibians**	2,060	**1,082 (52.5%)**	293 (14.2%)	301 (14.6%)	213 (10.3%)	171 (8.3%)	31.5
**Birds**	2,120	387 (18.3%)	**911 (43%)**	442 (20.8%)	292 (13.8%)	88 (4.2%)	37.2
**Mammals**	1,277	337 (26.4%)	259 (20.3%)	216 (16.9%)	**354 (27.7)**	111 (8.7%)	51.5
**Total**	5,457	**1,806 (33.1%)**	1,463 (26.8)	959 (17.6%)	859 (15.7%)	370 (6.8%)	38.4

The most common category for each taxon is shown in bold.

### Global hotspots of human impact

**Fig 3:**
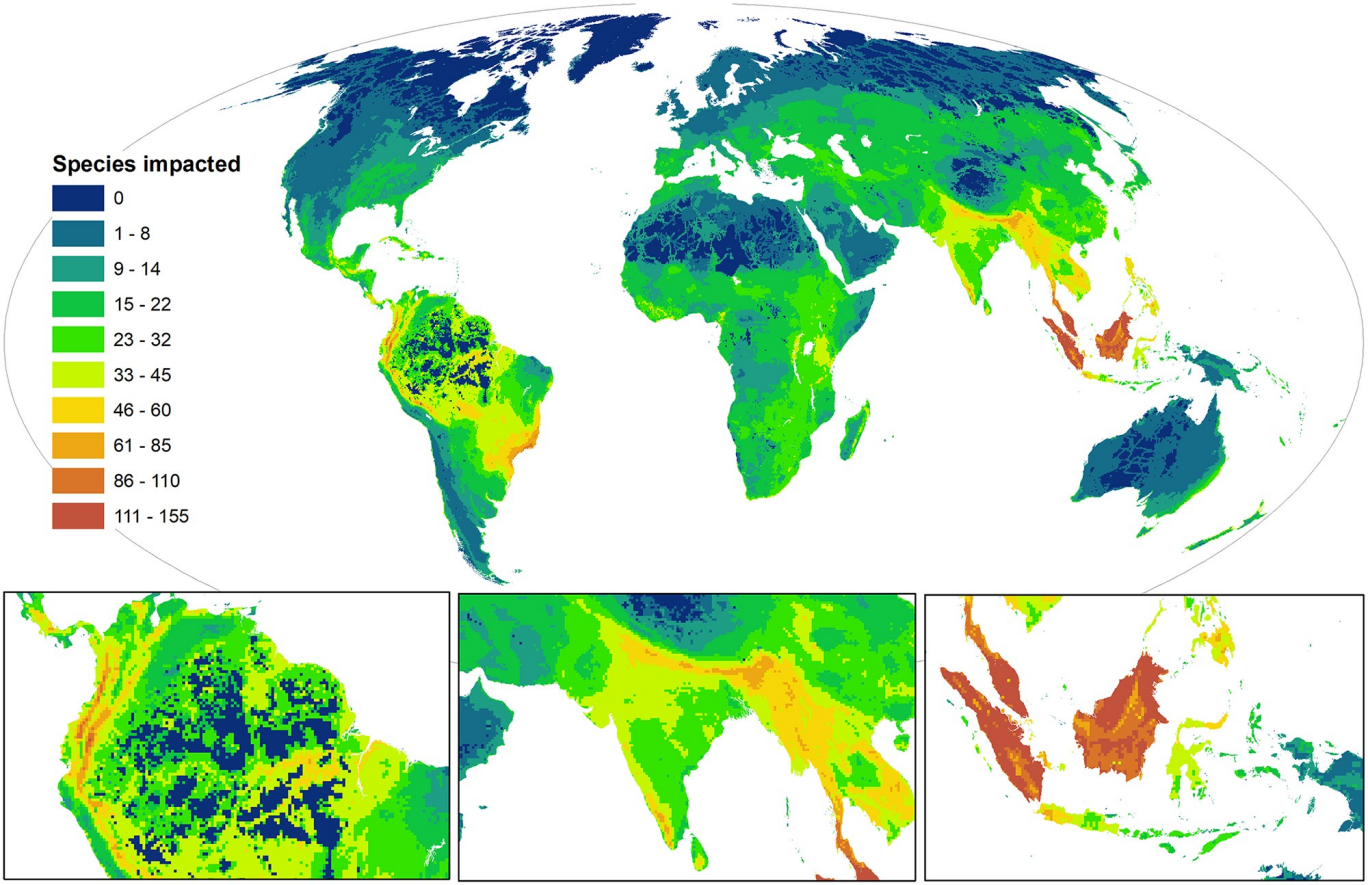
Cumulative human impacts on threatened and near-threatened terrestrial vertebrates (*n* = 5,457). Legend indicates the number of species in a grid cell impacted by at least one threat. Maps use a 30 km × 30 km grid and a Mollweide equal area projection. The data underlying this figure are freely available [31] (doi:10.1594/PANGAEA.897391).

Human impacts on threatened vertebrates are widespread, extending across 84% of Earth’s terrestrial surface (S2 Table; S1 and S2 Figs). There is strong spatial variation in the intensity of human impacts, with alarming peaks in Southeast Asia (Fig 3). Hotspots of human impact differ spatially between taxa (S3 Fig) and, as expected, are largely driven by patterns of threatened species richness (S4 Fig) and human pressure, although they are not congruent.

The top five countries most impacted by anthropogenic threats to species are all found in Southeast Asia (S3 Table), which we confirm is overwhelmingly the dominant global hotspot of impacts to species [32]. Malaysia has the highest average human impact score (125 species impacted per grid cell), followed by Brunei and Singapore (124 and 112 species, respectively). These scores are substantially higher than the global average of 15.6 species impacted per grid cell. Concerningly, there are 13 grid cells (11,700 km^2^) in Southeast Asia where >150 species are impacted by threats.

When aggregated across biomes and ecoregions, which represent distinct biogeographic spatial units at the global scale [33] (S4 and S5 Tables), the highest human impacts are in mangroves, where on average 35 species are impacted per grid cell. Human impacts are also high throughout the tropical forests that harbour Earth’s richest biota and are critically important for biodiversity conservation [34]. The tropical and subtropical moist broadleaf forests in Southeast Brazil, Malaysia, and Indonesia are the second most impacted biome, followed by the tropical and subtropical dry broadleaf forests in India, Myanmar, and Thailand (35 and 34 species impacted per 900 km^2^ grid cell).

### Global coolspots of threat refugia

**Fig 4:**
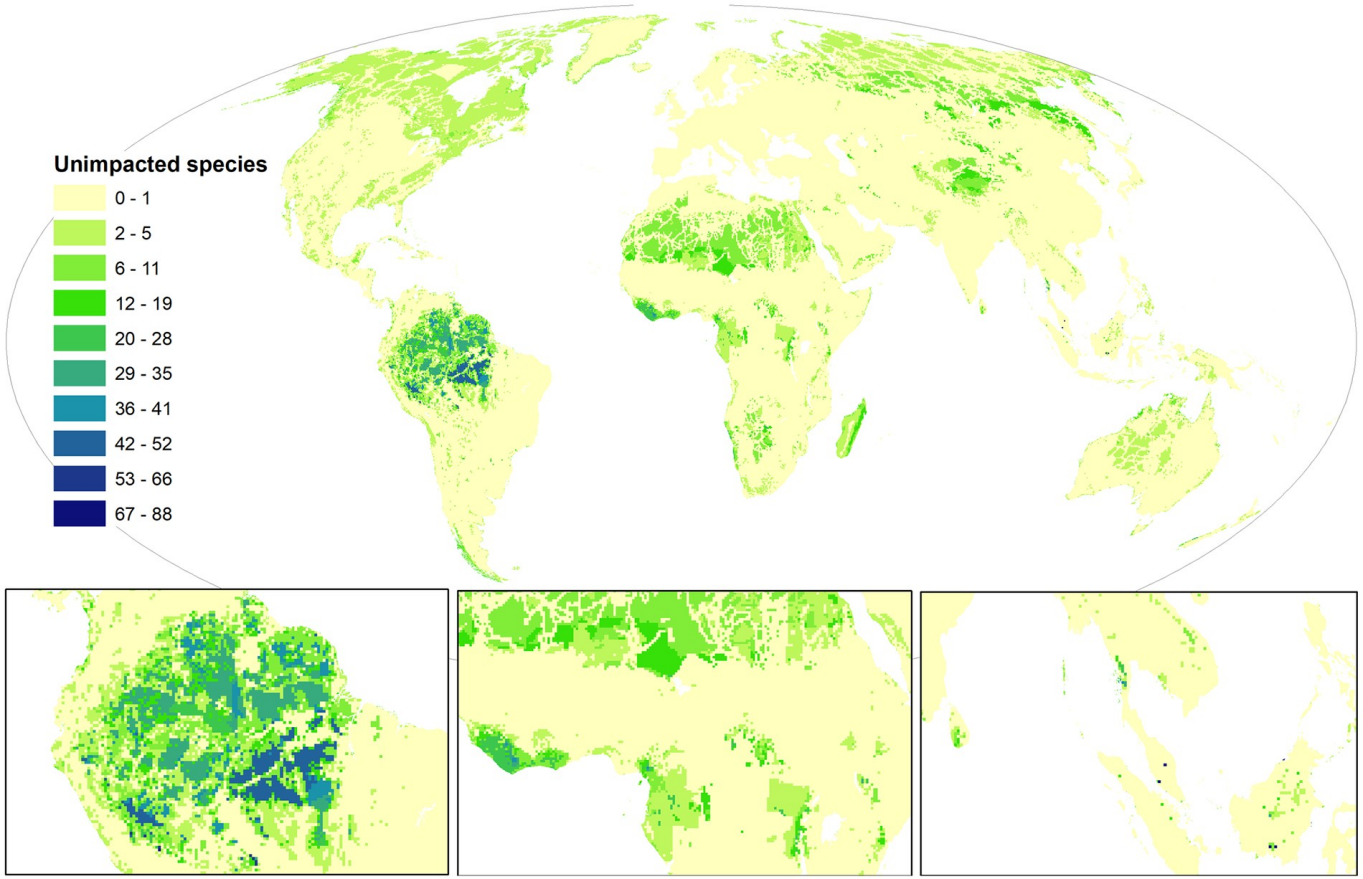
Coolspots of refugia for threatened and near-threatened terrestrial vertebrates (*n* = 5,457). Legend indicates the number of species that are not impacted by any threats in a grid cell. Maps use a 30 km × 30 km grid and a Mollweide equal area projection. The data underlying this figure are freely available [31] (doi:10.1594/PANGAEA.897391).

We mapped threat refugia for threatened vertebrates by combining the unimpacted parts of each species’ distribution. Less than half of Earth’s surface (43%) hosts at least one unimpacted threatened species, acting as a potential refugium for that species (Fig 4); however, impacted and unimpacted species co-occur across 28% of Earth’s surface, identifying places where species with divergent sensitivities to threatening processes are present. There is strong spatial variation in the intensity of threat refugia for threatened species and between coolspots for different taxa (S5 Fig). The Amazon rainforest is the overwhelmingly dominant global coolspot. Interestingly, threat refugia follow similar patterns to hotspots of impact in many places, including parts of East Africa, Southeast Asia, and the Amazon. Although counterintuitive, our results are driven by species richness and individual species’ different sensitivities to threats. Therefore, in species-rich areas, it is logical that many species will be impacted, whilst many others remain unimpacted. The highest average threat refugia score is in Liberia (23 species unimpacted per grid cell), but the highest score for an individual grid cell occurs in Malaysia, where 144 species are unimpacted. Encouragingly, there are 12 grid cells (10,800 km^2^) in Southeast Asia with >60 unimpacted species, although this is primarily due to the large number of threatened species in the region.

Other coolspots of threat refugia include Liberia in West Africa, the Albertine Rift Valley in East Africa, and Southern Myanmar. When aggregated across biomes and ecoregions (S4 and S5 Tables), the tropical and subtropical moist broadleaf forests and tundra act as the greatest threat refugia, supporting on average 5.2 and 2.5 unimpacted species per grid cell, respectively. The tropical and sub-tropical moist broadleaf forests are also one of the most impacted biomes, demonstrating that despite this, there is still considerable conservation opportunity here. The tundra is the only biome where more species are unimpacted than impacted on average.

### Proportion of species impacted

**Fig 5:**
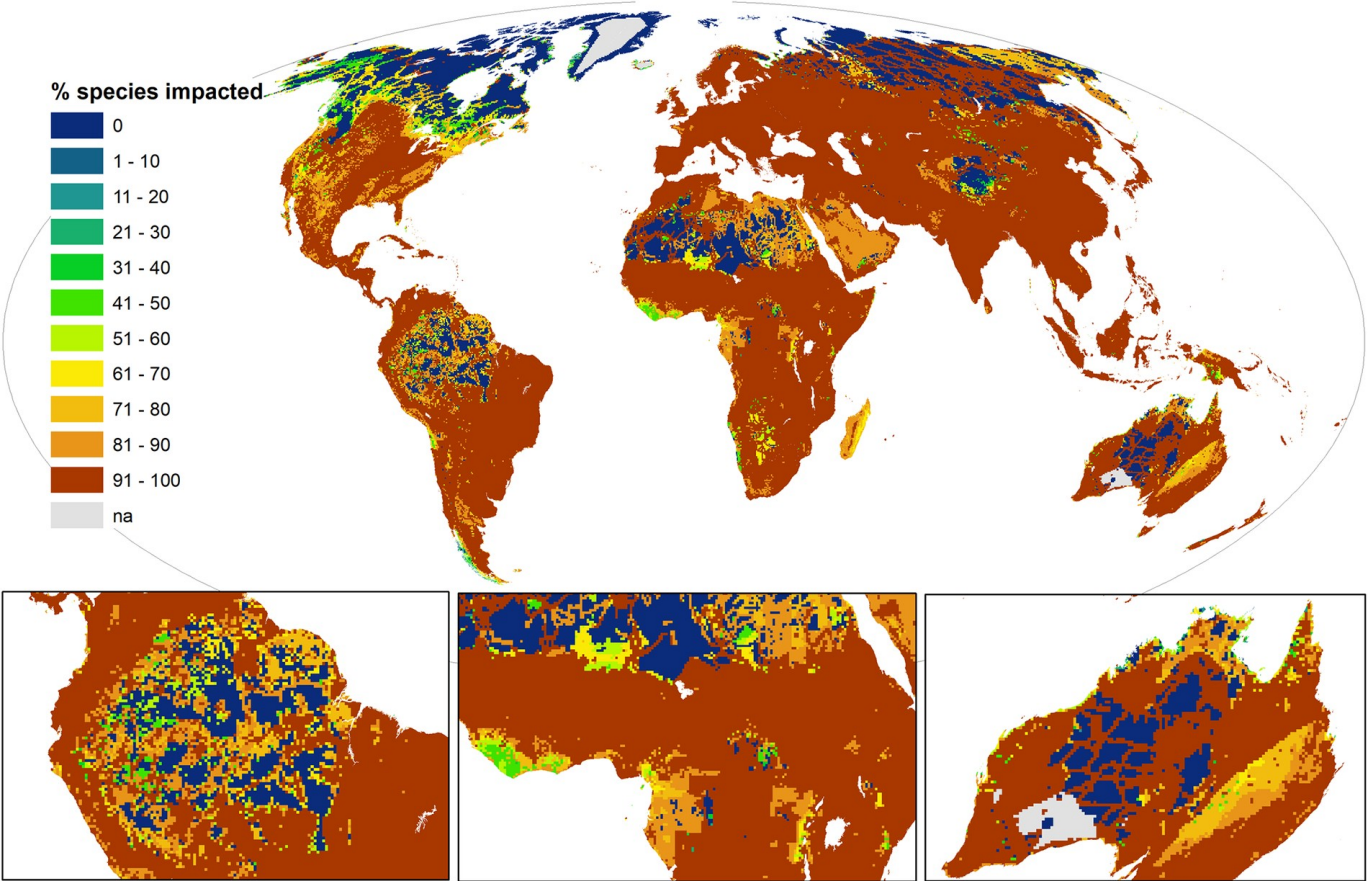
The percentage of species in a grid cell impacted by a threat (and inversely the number of unimpacted species for whom it is a refuge) for all taxa (*n* = 5,457). Maps use a 30 km × 30 km grid and a Mollweide equal area projection. The data underlying this figure are freely available [31] (doi:10.1594/PANGAEA.897391).

Some areas of the planet contain low numbers of threatened species (for example, the high latitudes or arid and desert regions). Therefore, it is instructive to examine the corresponding proportions of impacted versus unimpacted species. On average, there are more impacted than unimpacted species in a grid cell globally (15.6 versus 1.9; ratio 8) (Fig 5; S6 Fig). The proportion varies for taxonomic groups, with amphibians having the highest ratio of impacted versus unimpacted species (2.3 versus 0.2; ratio 12.2) compared to birds and mammals (birds 10.5 versus 1.3, ratio 8.1 and mammals 5.4 versus 0.7, ratio 7.8).

In our 30 km^2^ grid cells, the proportion of species impacted extends across the full range from 0%–100%. We found that >90% of species were impacted in 107,102 grid cells globally, amounting to a staggering 96 million km^2^ (66.7% of Earth’s terrestrial area). Encouragingly, species are present, but none are impacted in 23,865 grid cells (21.5 million km^2^; 14.8% of Earth’s terrestrial area). The majority of this is wilderness where no human pressures occur. However, we found 426 grid cells (383,400 km^2^; 0.27% of terrestrial area) where a species and a human pressure co-occur, but there is no impact (i.e., none of the species present are sensitive to the human activity or land use occurring in that area).

The distribution of areas with high proportions of impacted species is extensive and differs substantially from hotspots of human impact. Europe, North and Central America, and Africa now emerge as hotspots, particularly for mammals and amphibians. The proportion of birds impacted presents a more spatially homogenous pattern, with hotspots in Southeast Asia and the Southeast South America. When aggregated across biomes, tropical and subtropical dry broadleaf forests have the highest mean proportion of impacted species (98.3%), followed by temperate grasslands, savannas and shrublands (97.8%) (S4 Table). The tundra and boreal/taiga forests have the lowest mean proportions of impacted species (48.6% and 60.3%, respectively).

## Discussion

### Implications for biodiversity conservation

Our results represent the current best estimate of the spatial distribution of human impacts on terrestrial vertebrates. Continued extirpations, the precursors of extinction, will continue to occur in the impacted portions of species ranges, which our results demonstrate are substantial. Consequently, completely impacted species or those persisting in threat refugia that are too small to support viable populations in the long term [35] likely face imminent extinction. These findings complement recent work showing that hundreds of mammals have lost considerable portions of their historic distributions [36] and that habitat fragmentation has greatly reduced the proportion of highly suitable habitat within species distributions, reducing their movements [37] and increasing their extinction risk [38].

Although our results are concerning, there is room for hope. The threats we map can be mitigated by in situ conservation actions, but diverse approaches are required. To ensure the survival of highly impacted species with little or no threat refugia, active threat management, restoration, and rewilding efforts [39] are needed to open up enough viable habitat for species to persist. Conservation action in the hotspots of human impact we identify will have high benefits since they are areas with exceptionally high threatened species richness and species-specific threats [40]. Our results therefore extend previous efforts to identify biodiversity hotspots [40], which were developed following somewhat similar logic and have helped guide conservation action and millions of dollars of funding. The hotspots of human impact we identify are priorities for actions that mitigate the specific threats [41].

Rather than being purely reactive and focusing solely on securing a future for imperilled species in the short term, conservation efforts would also benefit from proactively securing coolspots of species refugia and avoiding any initial human impacts in these places [42]. This will help ensure many species’ long-term persistence, especially in a time of rapid climate change, where areas free of threatening processes will be critical for species adaptation [43,44]. Securing refugia will be particularly effective if protection is targeted at the most species-rich places that currently remain threat free but may soon be jeopardised [45,46]. Additionally, conservation action is also likely to have a high chance of success in threat refugia and be more cost effective [47,48]. Proactive and reactive approaches to conservation have historically been pitted against each other [49], with reactive approaches deemed more urgent and taking precedence [49–51]. However, our discovery of the spatial overlap existing between hotspots of impacted species richness and coolspots of unimpacted species richness provides opportunities for multifaceted conservation action that is reactive for some species while simultaneously being proactive for others.

The utility of our work extends beyond conservation and can inform sustainable development planning. Conservation action within some of the hotspots of impact we identified (especially in Southeast Asia) are likely to deliver synergistic benefits to other environmental goals, such as carbon conservation and global reduction of deforestation rates [52]. Additionally, according to our definition, species threat refugia do not necessarily have to be off limits to human development, just free of the actions and land usage that directly threaten species found in that area. This provides a unique framework for quantifying the tradeoffs associated with the development of alternate human activities and land usage and for identifying locations and strategies to minimise their impacts on biodiversity. This has implications for nations striving to meet ambitious development targets such as the United Nations Sustainable Development Goals (SDGs), especially where achieving development goals involves tradeoffs with biodiversity goals [53,54]. The framework presented here could be adapted to inform conservation and development planning from local to regional scales and could be particularly useful in Southeast Asia, Latin America, and sub-Saharan Africa, regions that are undergoing rapid economic development but are also hotspots of human impact and coolspots of threat refugia [55,56].

It is important to note that our data are not comprehensive of all threats to all species. For example, our analysis does not take into account infectious diseases, a driver of global declines in amphibians [57], or climate change, a threat already impacting many species across all taxa [43]. The results are therefore conservative, and many species will be more impacted than our maps indicate. Notably, one of the fundamental ways to manage global-scale threats such as climate change is to stop more easily abatable threats such as those considered in this analysis [58] to avoid antagonistic or synergistic interactions between multiple threats [59,60]. Other caveats worthy of discussion are that we assume the intensity of threats (for example, agricultural land use or roads) are equal across their distribution and that species are equally sensitive to each threat known to affect them. This assumption could mean we are overestimating impacts in cases in which species are sensitive to several threats where only the secondary threat is present. The IUCN has collected data on the severity of threats to species, but a comprehensive database is still lacking because this information is often unknown. The further development of these data would allow important nuances to be included in future extensions of this work.

A species and threat overlap does not necessarily mean that the threat is acting in that location. However, our analysis extends beyond a species threat overlay by incorporating three co-occurring and connected forms of data: a species distribution, a threat distribution, and that species’ vulnerability to that threat. To the best of our knowledge, this is the first time species-specific sensitivity to threats has been incorporated into an impact mapping exercise at this scale. By mapping species-specific threats, it is much more likely that a threat is acting in a given location and impacting a species. This approach does rely on the current knowledge of threats to species and cannot account for the possibility that undocumented threats could be impacting a species. We sourced information on threats to species from the IUCN, who are the main authority on assessing species extinction risk, and limited our analyses to threatened terrestrial vertebrates, which include the most studied taxa globally [61]. Yet, it is important to note that there is still variation between species assessments because of taxonomic and geographical biases that could influence our findings [62]. For example, our understanding of threats to mammals is greater than for amphibians, which could partly explain why our results show mammals as the most impacted taxon, whilst amphibians are generally regarded as the more threatened taxon.

This analysis provides a framework for mapping human impacts that represents a conceptual advance over cumulative pressure mapping or threatened-species-richness mapping that can be applied to any scale, taxa, or realm. Furthermore, the framework and baseline can be continually updated and enhanced as additional data on species distributions, their sensitivity to threats, and the spatial distribution of threats become available and our understanding of threat interactions improves. Improvements in our understanding of species sensitivity to threats will also allow this analysis to be extended to other forms of life such as plant and invertebrate species. We have shown that human impacts on species are almost ubiquitous across Earth and that hundreds of species have no refuge from these impacts, including many of the most charismatic large mammals. The survival of these species, and many more, hinges on humanity’s ability and willingness to compromise and share space.

## Materials and methods

### Spatial data on threatened species ranges

We focused our analysis on terrestrial vertebrate groups (amphibians, birds, mammals) with distribution maps and assessment of identified threat available for all species. Spatial data on mammal and amphibian distributions were obtained from the IUCN Red List of Threatened Species [3] and bird distributions from Birdlife International and NatureServe [63]. We focused on species which are listed as near threatened, vulnerable, endangered, or critically endangered since their major threats have been identified and comprehensively assessed for the IUCN Red List of Threatened Species [3,4,64,65]. Following established practice, we only considered native and reintroduced parts of each species distribution range in our analysis, which are listed as extant, possibly extant, or possibly extinct within their range [66]. We excluded introduced, vagrant, and extinct species as well as species whose origin or presence is uncertain. Although reintroduced species ranges may be theoretically subject to fewer threats, they may still be under threats not realised during the reintroduction process [67]. As such, incorporating all portions of a species range, including reintroduced areas, can provide a robust picture of the threats for a given species. Finally, we only included species whose distribution overlapped (even just partially) with the extent of the Human Footprint threat dataset, which does not include Antarctica. A total of 2,060 amphibian species, 2,120 bird species, and 1,277 mammal species qualified for our analysis based on these criteria.

### Spatial data on threats to species

Spatially explicit data on the distribution of threats to species were obtained from the recently updated Human Footprint maps [1,68]. These are globally standardised maps of cumulative human pressures on the natural environment at 1 km^2^ resolution globally for eight of the most harmful pressures humans exert on nature, including 1) built environments, 2) population density, 3) electric infrastructure, 4) crop lands, 5) pasture lands, 6) roads, 7) railways, and 8) navigable waterways. This makes the Human Footprint the most up-to-date and comprehensive global cumulative pressure/threat map available [29]. The Human Footprint is also the first global-scale threat dataset to have been validated for accuracy. This was done by visually confirming if human pressures were present or absent across thousands of randomly selected 1 km × 1 km plots globally [68]. The data were found to exhibit an excellent degree of accuracy (88.5% agreement between visual plots and Human Footprint data), especially at identifying threat-free areas (98.9% agreement between visual plots and wilderness) [69].

In the Human Footprint, each pressure layer is scaled between 1 and 10 based on its estimated impact on the environment. These scores are then cumulated in each pixel to give a total score out of 50. We converted these scores to binary (present or absent in any 1 km^2^ pixel) for our analyses since there are no data on the relative severity of individual threats to species. To convert pressure layers from continuous scales to binary (present/absent), we set cutoffs at which the pressure was considered absent. For example, roads have a direct pressure score of 8 up to 500 meters either side; beyond this, the pressure score decays exponentially from a score of 4 out to 0 at 15 km. When converting this to a binary score, we set a threshold that considered the pressure present up to 3 km either side of the road, and absent beyond this (see S6 Table for comprehensive details on how each layer was handled).

### Mapping species-specific threats

We identified cases where the eight pressures in the updated Human Footprint dataset directly or indirectly correspond with threats to biodiversity as listed in the IUCN Red List [30] (Table 1, S1 Table). This allowed us to globally map seven major classes and 15 subclasses of threats. Although this is not comprehensive of all the threats to species, it importantly includes the biggest drivers of biodiversity declines globally [4]. For example, multiple forms of agriculture, urban development, and transportation corridors are directly accounted for by our pressure data, whilst biological resource use and overexploitation through hunting, pollution, human disturbance, and invasive species are indirectly accounted for by human population density, roads and navigable river networks that act as proxies [56,58,70–72].

### Analysing the extent of human impacts on individual species

For a pressure to impact a species, it must spatially overlap with that species’ distribution and have been identified in the IUCN Red List as a threat to that species [19]. Therefore, we calculated the extent of overlap between each species distribution and each pressure layer that that species is sensitive to at a 1 km^2^ resolution globally. We accounted for the overlap between threats, identifying where multiple threats are present. All spatial data were analysed in a Mollweide equal area projection in ESRI ArcGIS and PostGIS, and statistics were calculated in R statistical software. We used a one-way analysis of variance to test for correlation between a species extinction risk category and the proportion of that species range impacted by threats.

### Mapping hotspots of cumulative human impacts

We estimated cumulative human impacts on threatened species using a global 30 km × 30 km planning unit grid, since this has been identified as the ideal resolution for reducing the effects of commission errors (where species are thought to be present but are not) when working with species range maps [73]. An impact was scored in a grid cell if a species and at least one threat it is sensitive to were both present. This means that the presence of a threat and a species in the same grid cell is not considered an impact unless the species is known to be sensitive to that threat. We then calculated the sum of all impacted species in a grid cell to give a total estimate of cumulative human impact.

As a sensitivity analysis, we calculated the area of each species distribution within each planning unit and the area of each pressure in each planning unit, converting both to proportions of planning unit area. To estimate how impacted each species is within each planning unit, we multiplied the proportion of the species distribution by the proportion of each pressure that threatens the species and then summed the scores. By using the proportion of planning unit area, we scale for the likelihood of a species and a pressure overlapping within a grid cell. Finally, we calculated the sum of all the individual species impact scores within each grid cell to give a total estimate of cumulative human impact. Spatial patterns of impact were strongly coherent between the two approaches, so we report on the more intuitive binary metric in the manuscript. We also ran a multiple linear regression on 10,000 randomly selected grid cells comparing the binary impact metric reported in the paper (a species and ≥1 threat = 1 impact in a grid cell) (response variable) and species richness and the mean human footprint in a grid cell as predictor variables. We obtained an R^2^ value of 0.9, which shows that the human footprint and richness explain 90% of the variation in the model but also suggests that including species sensitivities to threats explains the other 10% of the variation. When we incorporate cumulative impacts (1 species + 3 threats = 3 impacts in a grid cell) and rerun the multiple linear regression, the R^2^ drops to 0.77, suggesting that in areas where multiple threats are present, including species-specific threats is particularly important.

### Mapping coolspots of threatened vertebrate anthropogenic refugia

We followed similar methods to mapping human impacts, where a cell was scored as an anthropogenic refuge if a species was present in the cell but no pressures that threaten it were present. These were then summed to give a cumulative score of the number of unimpacted species in a cell.

## Supporting information

S1 Fig Impact hotspots of individual human pressures on all threatened terrestrial vertebrates (*n* = 5,457), mammals (*n* = 1,277), birds (*n* = 2,120), and amphibians (*n* = 2,060). Scale represents the number of species impacted by the threat in a grid cell. Hotspots of impact are in dark red. Maps use a 30 km × 30 km grid and a Mollweide equal area projection. The data underlying this figure are freely available [31] (doi:10.1594/PANGAEA.897391).
(TIF)

S2 Fig Impact hotspots of individual human pressures on all threatened terrestrial vertebrates (*n* = 5,457), mammals (*n* = 1,277), birds (*n* = 2,120), and amphibians (*n* = 2,060). Scale indicates the number of species impacted by the threat in a grid cell. Hotspots of impact are dark red. Maps use a 30 km × 30 km grid and a Mollweide equal area projection. The data underlying this figure are freely available [31] (doi:10.1594/PANGAEA.897391).
(TIF)

S3 Fig Cumulative human impacts on all threatened terrestrial birds (*n* = 2,120), mammals (*n* = 1,277), and amphibians (*n* = 2,060). Scale indicates the number of species in a grid cell impacted by at least one threat. Areas of high human impact (hotspots) are red. Maps use a 30 km × 30 km grid and a Mollweide equal area projection. The data underlying this figure are freely available [31] (doi:10.1594/PANGAEA.897391).
(TIF)

S4 Fig Threatened species richness for all taxa (*n* = 5,457), mammals (*n* = 1,277), birds (*n* = 2,120), and amphibians (*n* = 2,060). Areas of high human richness are red. Maps use a 30 km × 30 km grid and a Mollweide equal area projection. The data underlying this figure are freely available [31] (doi:10.1594/PANGAEA.897391).
(TIF)

S5 Fig Coolspots of refugia for all threatened terrestrial mammals (*n* = 1,277), birds (*n* = 2,120), and amphibians (*n* = 2,060). Scale indicates the number of species not impacted by any threats in a grid cell. Coolspots of refugia are blue. Maps use a 30 km × 30 km grid and a Mollweide equal area projection. The data underlying this figure are freely available [31] (doi:10.1594/PANGAEA.897391).
(TIF)

S6 Fig The percentage of species in a grid cell impacted by a threat (and inversely, the number of unimpacted species for whom it is a refuge) for (A) birds (*n* = 2,120), (B) mammals (*n* = 1,277), and (C) amphibians (*n* = 2,060). Maps use a 30 km × 30 km grid and a Mollweide equal area projection. The data underlying this figure are freely available [31] (doi:10.1594/PANGAEA.897391).
(TIF)

S1 Table Major classes and subclasses of threats to biodiversity, as classified in the IUCN Red List of Threatened Species, the corresponding spatially explicit human pressure variable from the updated Human Footprint dataset and a brief description of how it was created, along with justifications for linking spatially explicit pressures to threats. IUCN, International Union for Conservation of Nature.
(DOCX)

S2 Table The eight mapped human pressures, the number of sensitive species they impact, the area in which these impacts are occurring, and the proportion of Earth’s terrestrial area where these impacts are occurring. (DOCX)

S3 Table The top ten countries with the most impacted and unimpacted species on average. (DOCX)

S4 Table The average number of species impacted and unimpacted by threats per grid cell, and the proportion of species impacted by threats, in each of Earth’s biomes. (DOCX)

S5 Table The average number of species impacted by threats per grid cell, and unimpacted by threats per grid cell, in each of Earth’s ecoregions. (DOCX)

S6 Table Weights assigned to individual pressures in the Human Footprint and threshold scheme used to convert pressures into binary scores (present or absent) for impact analyses. (DOCX)

S1 Data Database containing information on the area and proportion of a threatened vertebrate species’ range that is impacted by threats, including the data underpinning Fig 2. (XLSX)

## Abbreviations

IUCN: International Union for Conservation of Nature
SDG: United Nations Sustainable Development Goal
