# Correction: Hotspots of human impact on threatened terrestrial vertebrates

**DOI:** 10.1371/journal.pbio.3000598

**Published:** 2019-12-16

**Authors:** James R. Allan, James E. M. Watson, Moreno Di Marco, Christopher J. O’Bryan, Hugh P. Possingham, Scott C. Atkinson, Oscar Venter

## Notice of republication

This article was republished on 2^nd^ December 2019, to correct errors in analysis that led to each pixel in Fig 4 displaying the number of species not impacted by *at least one* threat, rather than the number of species that were not impacted by *any* threats.

This error subsequently affected the section entitled “Proportion of species impacted” and so species richness was overestimated, resulting in the values in Fig 5 also being incorrect.

The following files have been reproduced, in light of the errors above:

Fig 4, Fig 5, S4-S6 Figs, S3-S5 Tables.

Please download this article again to view the correct version. The originally published, uncorrected article is provided here for reference.

## Supporting information

S1 File Originally published, uncorrected article. (PDF)
