# Supplementary figures and images for: Hotspots of human impact on threatened terrestrial vertebrates
Source: PLoS Biol. 2019 Mar 12;17(3):e3000158. doi: 10.1371/journal.pbio.3000158 (PMC6413901; doi:10.1371/journal.pbio.3000158)

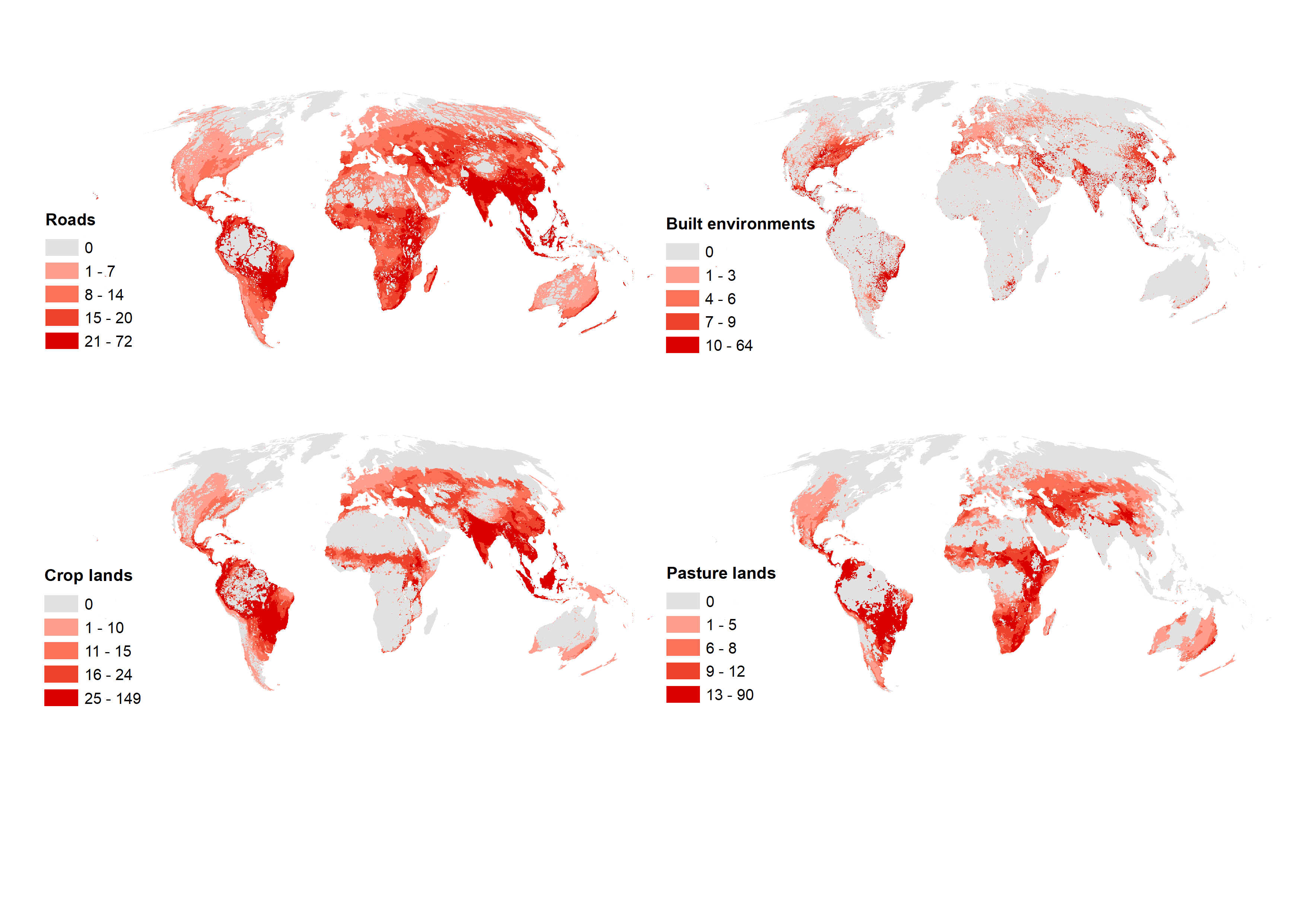

Supplement: S1 Fig — Scale represents the number of species impacted by the threat in a grid cell. Hotspots of impact are in dark red. Maps use a 30 km × 30 km grid and a Mollweide equal area projection. The data underlying this figure are freely available [31] (doi:10.1594/PANGAEA.897391). (TIF) [file pbio.3000158.s001.tif]

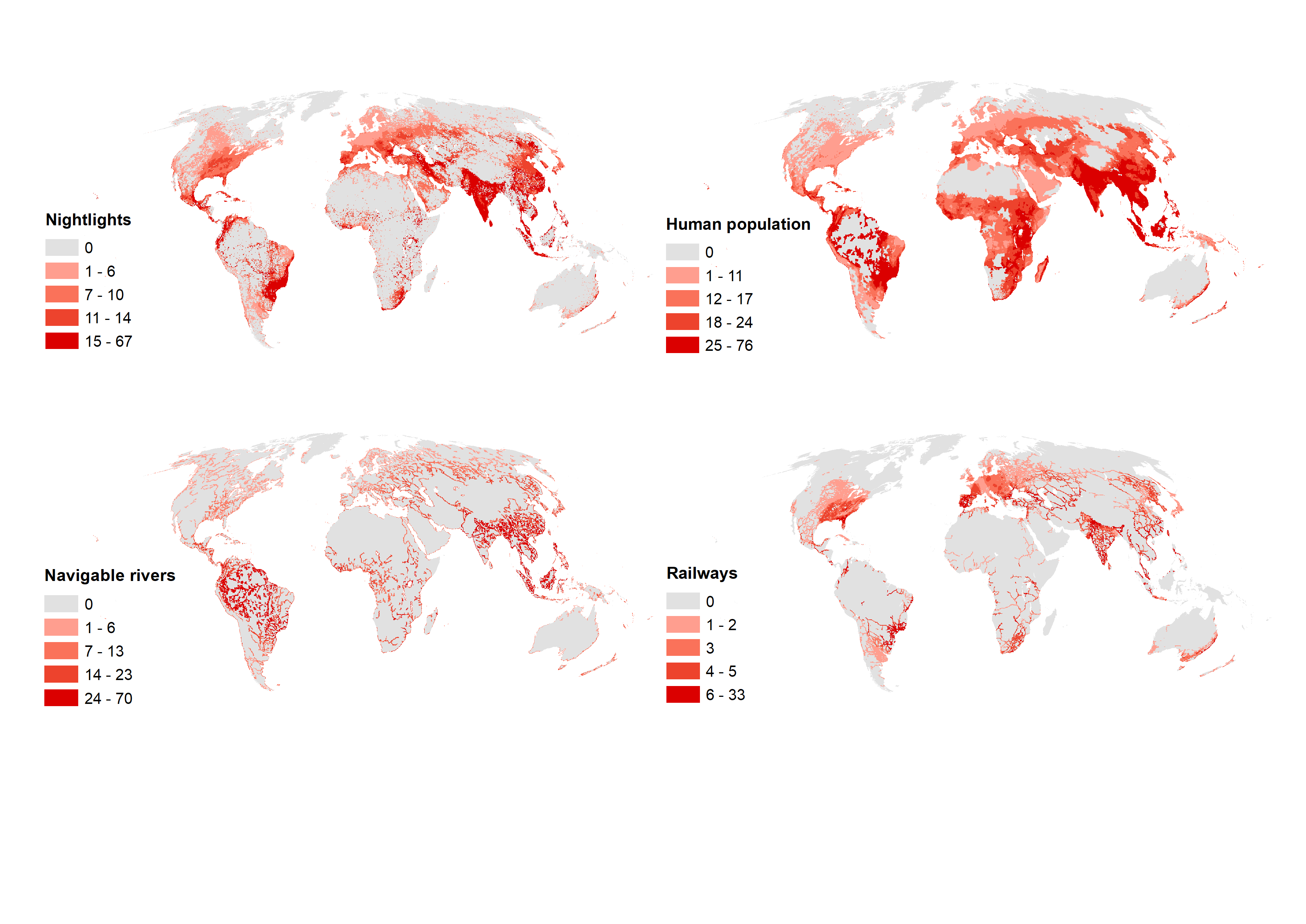

Supplement: S2 Fig — Scale indicates the number of species impacted by the threat in a grid cell. Hotspots of impact are dark red. Maps use a 30 km × 30 km grid and a Mollweide equal area projection. The data underlying this figure are freely available [31] (doi:10.1594/PANGAEA.897391). (TIF) [file pbio.3000158.s002.tif]

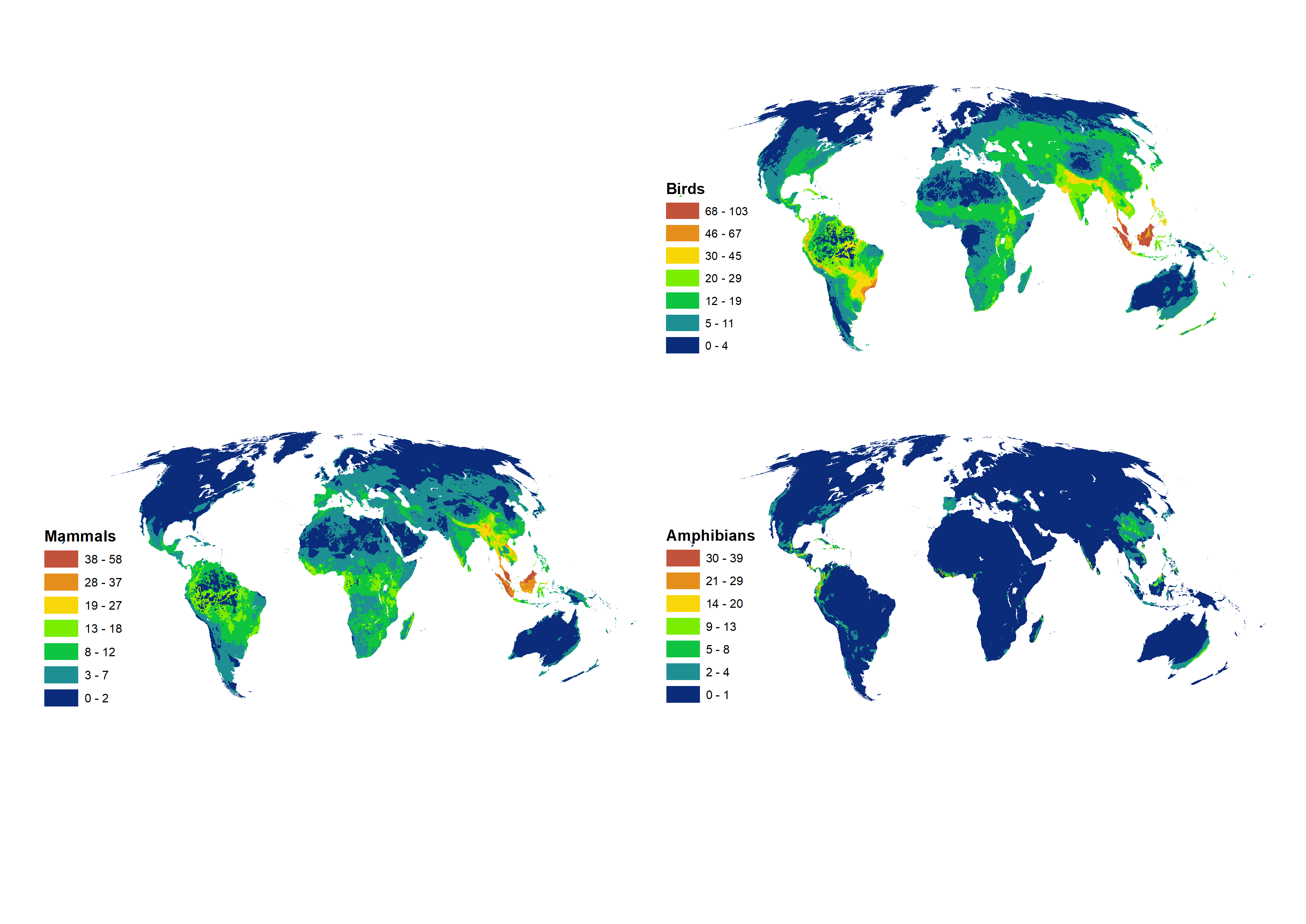

Supplement: S3 Fig — Scale indicates the number of species in a grid cell impacted by at least one threat. Areas of high human impact (hotspots) are red. Maps use a 30 km × 30 km grid and a Mollweide equal area projection. The data underlying this figure are freely available [31] (doi:10.1594/PANGAEA.897391). (TIF) [file pbio.3000158.s003.tif]

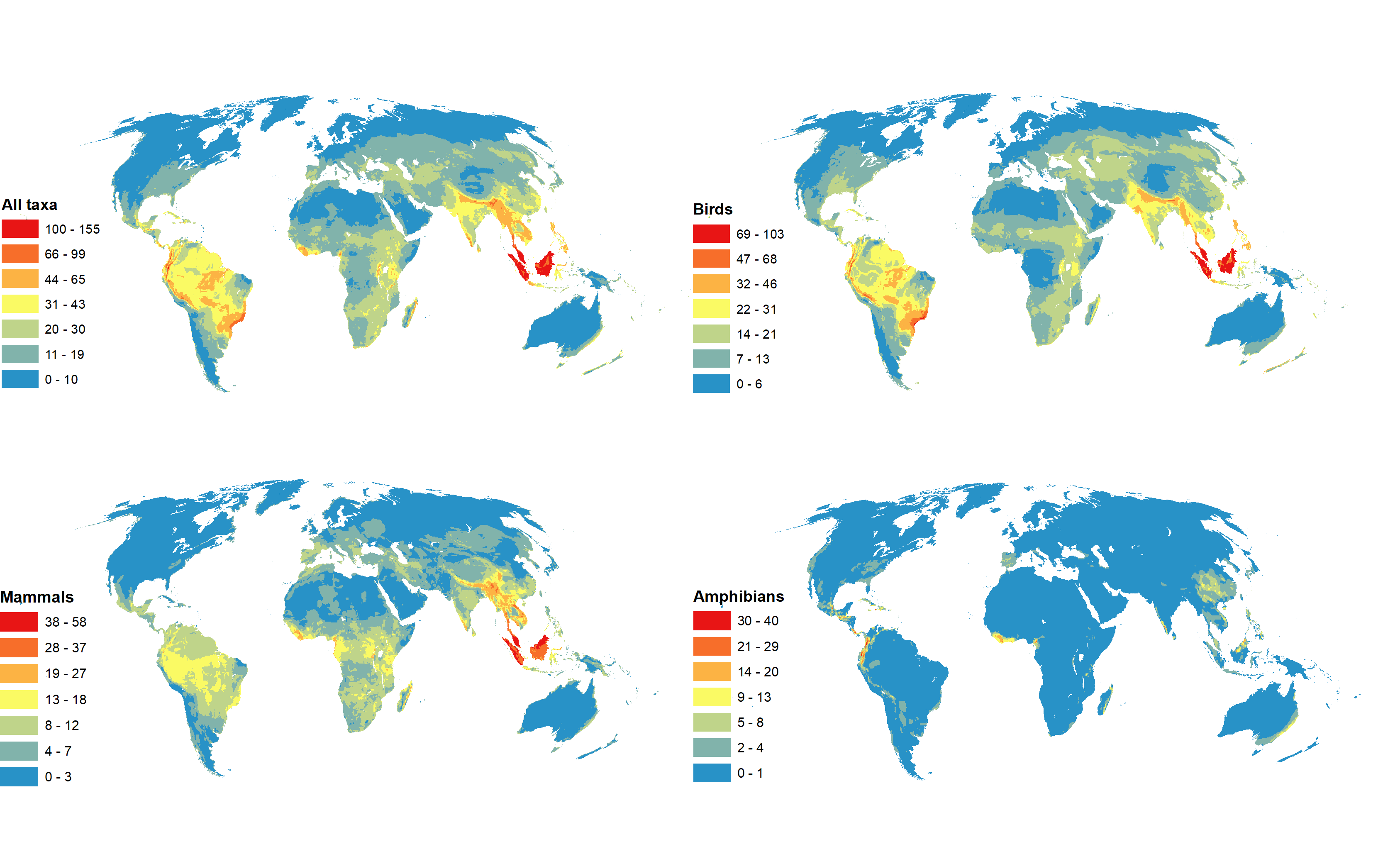

Supplement: S4 Fig — Areas of high human richness are red. Maps use a 30 km × 30 km grid and a Mollweide equal area projection. The data underlying this figure are freely available [31] (doi:10.1594/PANGAEA.897391). (TIF) [file pbio.3000158.s004.tif]

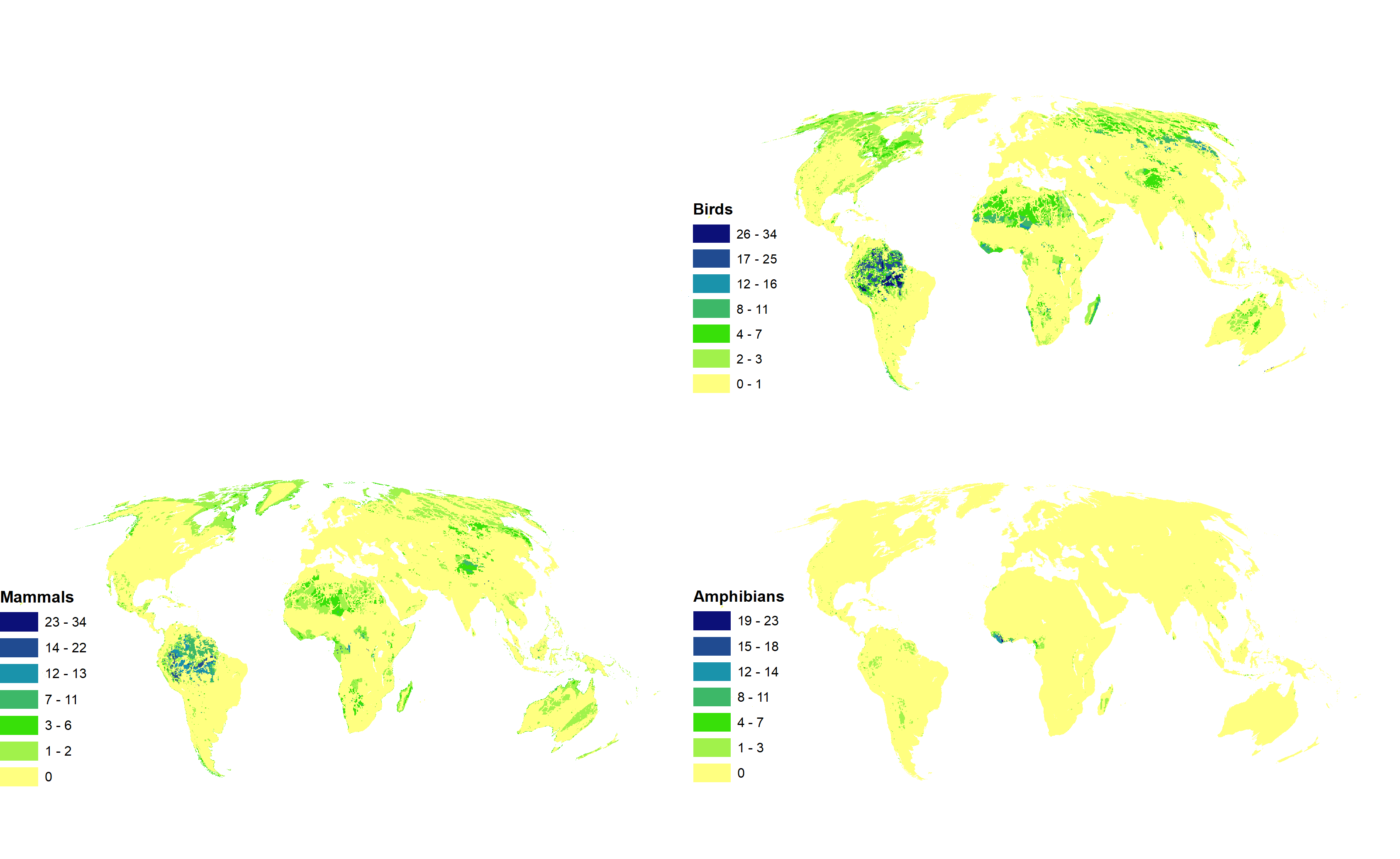

Supplement: S5 Fig — Scale indicates the number of species not impacted by any threats in a grid cell. Coolspots of refugia are blue. Maps use a 30 km × 30 km grid and a Mollweide equal area projection. The data underlying this figure are freely available [31] (doi:10.1594/PANGAEA.897391). (TIF) [file pbio.3000158.s005.tif]

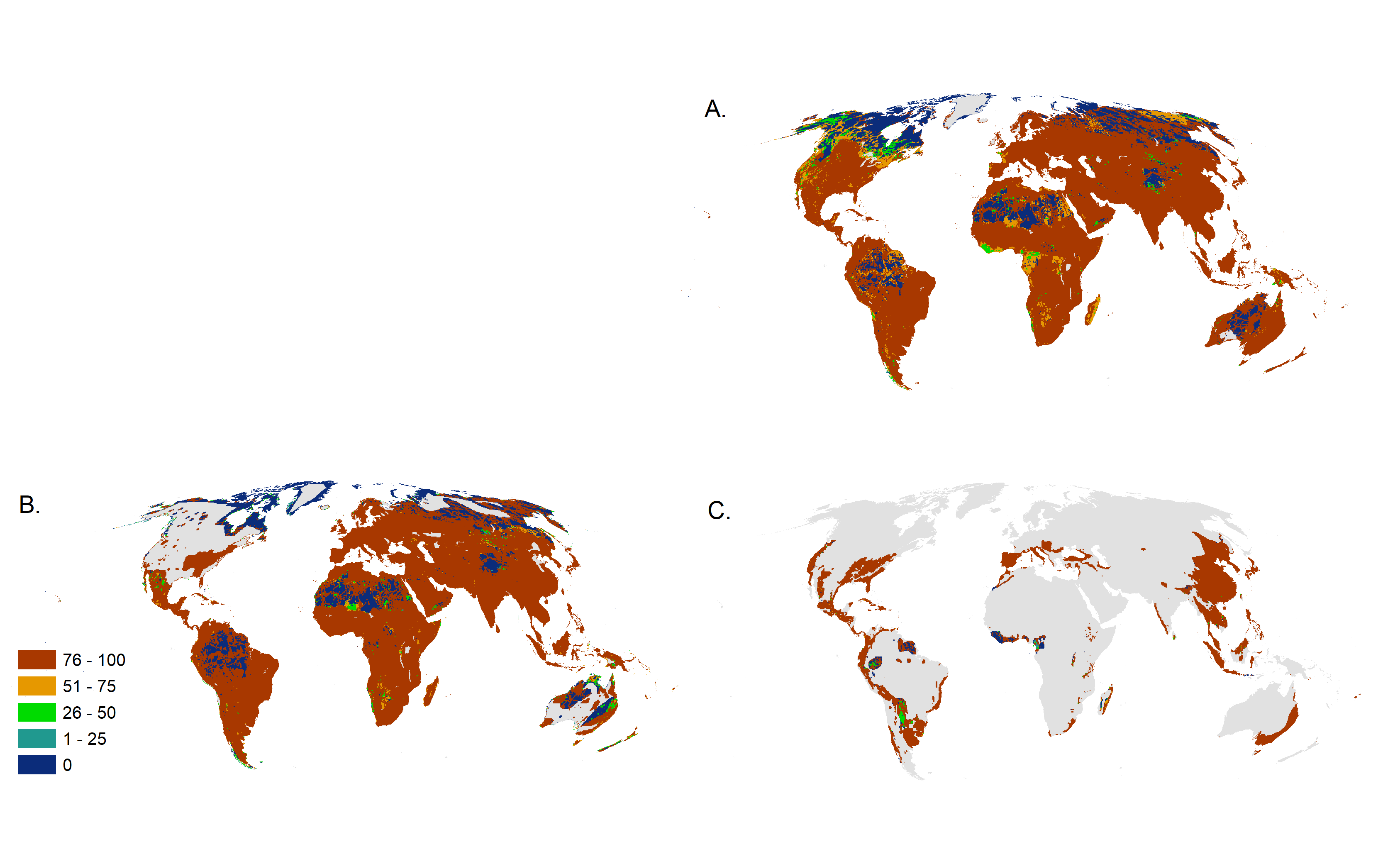

Supplement: S6 Fig — Maps use a 30 km × 30 km grid and a Mollweide equal area projection. The data underlying this figure are freely available [31] (doi:10.1594/PANGAEA.897391). (TIF) [file pbio.3000158.s006.tif]
