# Supplementary material for: Hotspots of human impact on threatened terrestrial vertebrates
Source: PLoS Biol. 2019 Mar 12;17(3):e3000158. doi: 10.1371/journal.pbio.3000158 (PMC6413901; doi:10.1371/journal.pbio.3000158)
Supplement: S1 Table — IUCN, International Union for Conservation of Nature. (DOCX) [file pbio.3000158.s007.docx]

**Table S1**. Major classes and sub-classes of threats to biodiversity, as classified in the IUCN Red List of Threatened Species, the corresponding spatially explicit human pressure variable from the updated Human Footprint dataset and a brief description of how it was created, along with justifications for linking spatially explicit pressures to threats.

| **Major threat class (IUCN)** | **Sub-class threats (IUCN)** | **Pressure (Human Footprint)** | **Description of Human Footprint Data (see Venter et al. 2016 for more detail)** | **Threat link** | **Examples as given by IUCN and justification based on references and prior knowledge** |
| --- | --- | --- | --- | --- | --- |
| 1. Residential & commercial development (definition: threats from human settlements or other non-agricultural land uses with a substantial footprint) | 1.1 Housing & urban areas (definition: human cities, towns, and settlements including non-housing development typically integrated with housing) | Electric infrastructure (nightlights) | Nightlights data [1] was used to map rural housing and suburban areas. This was done by setting brightness thresholds as described in Venter 2016. | Directly mapped | "*Urban areas, suburbs, villages, ranchettes, vacation homes, shopping areas, offices, schools, hospitals, birds flying into windows, land reclamation or expanding human habitation that causes habitat degradation in riverine, estuary and coastal areas, etc*." These examples are directly captured by the electric infrastructure and built environments layers in the human footprint. |
|  |  | Built environments | Built environments were mapped in the human footprint using data on nightlights (Elvidge et al. 2001). The data was thresholded at a brightness consistent with a city based on global analyses [2] and visual validation with Landsat imagery of 10 cities globally. | Directly mapped |  |
|  | 1.2 Commercial & industrial areas (def: factories and other commercial centres) | Electric infrastructure (nightlights) | Nightlights data (Elvidge et al 2001) was used to map working landscapes in the human footprint. | Directly mapped | "*military bases, factories, stand-alone shopping centres, office parks, power plants, train yards, ship yards, airports, landfills, etc.*" These examples are directly captured by the electric infrastructure and built environments layers in the human footprint. |
|  |  | Built environments | Bulit environments were mapped in the human footprint using data on nightlights (Elvidge et al. 2001). The data was thresholded at a brightness consistent with a city based on global analyses (Small et al. 2011) and visual validation with Landsat imagery of 10 cities globally. | Directly mapped |  |
| 2. Agriculture & aquaculture (definition: threats from farming and ranching as a result of agricultural expansion and intensification, including silviculture, mariculture and aquaculture (includes the impacts of any fencing around farmed areas)) | 2.1 Annual and perennial non-timber crops (definition: crops planted for food, fodder, fibre, fuel, or other uses) | Crop lands | Intensive agriculture was mapped in the human footprint using data obtained from GlobCover. The human footprint does not capture small scale shifting agriculture due to limitiations in the ability of remote sensing to corrrectly identify this land use. | Directly mapped | No examples given. The human footprint directly maps intensive agriculture. |
|  | 2.3 Livestock farming & ranching (definition: domestic terrestrial animals raised in one location on farmed or nonlocal resources (farming); also domestic or semi-domesticated animals allowed to roam in the wild and supported by natural habitats (ranching)) | Pasture lands | Land grazed by domestic herbivores was directly mapped in the human footprint using data from [3] | Directly mapped | No examples given. The human footprint directly maps grazing land. |
| 4. Transportation & service corridors (definition: threats from long narrow transport corridors and the vehicles that use them including associated wildlife mortality) | 4.1 Roads & railroads (definition: surface transport on roadways and dedicated tracks) | Railways | A map of railways was obtained from the National Imagery and Mapping Agency, Washington, DC. [4]. | Directly mapped | "*highways, secondary roads, primitive roads, logging roads, bridges & causeways, road kill, fencing associated with roads, freight/passenger/mining railroads, etc*." Highways and secondary roads are directly captured by the data (and associated fences and bridges indirectly captured). Primitive roads and logging roads are not captured. Railways are directly captured and associated fences indirectly captured. |
|  |  | Roads | gROADS data was used to map major roadways and contains many but not all minor roadways [5]. | Directly mapped |  |
|  | 4.2 Utility &service lines (definition: transport of energy & resources) | Roads | gROADS data was used to map major roadways and contains many but not all minor roadways (CIESEN 2013) | Indirect | "*electrical & phone wires, aqueducts, oil & gas pipelines, electrocution of wildlife, etc*." Electrical and phone wires closely follow roads, often directly parallelling them, therefore roads act as a good proxy in the absence of exact data. |
| 5. Biological resource use (definition: threats from consumptive use of "wild" biological resources including both deliberate and unintentional harvesting effects; also persecution or control of specific species) | 5.1 Hunting and collecting terrestrial animals (definition: killing or trapping terrestrial wild animals or animal products for commercial, recreation, subsistence, research or cultural purposes, or for control/persecution reasons; includes accidental mortality/bycatch) | Navigable waterways | Waterways (oceans, large lakes, rivers) are considered navigable 80km either side of a human settlement within 4 km of the coast. (80 km is the approximate distance a vessel can travel and return in daylight hours). Rivers also had to be > 2m deep. | Indirect | "*bushmeat hunting, trophy hunting, beaver trapping, butterfly collecting, honey or bird nest hunting, etc. pest control often impacts non-targeted species, hunter's dogs may chase after and kill other non-target species during a hunt, loss of a species' prey base due to over-harvesting by humans of their prey, etc wolf control, pest control, persecution of snakes because of superstition, etc*." Hunting and collecting animals is strongly influenced by accessibility [6, 7]. Therefore, we used roads and navigable waterways as proxies, along with human presence in an area (the population density layer in the human footprint was thresholded into a present/absent binary score; see methods). The relationship between roads and hunting is well founded globally, including in licensed or regulated [8-11]. |
|  |  | Population density | Human population density was mapped using the Gridded population of the world dataset, which provides a 4x4 km dataset globally [12]. | Indirect |  |
|  |  | Roads | gROADS data was used to map major roadways and contains many but not all minor roadways (CIESEN 2013) | Indirect |  |
|  | 5.2 Gathering terrestrial plants (definition: harvesting plants, fungi, and other non-timber/non-animal products for commercial, recreation, subsistence, research or cultural purposes, or for control reasons) | Navigable waterways | Waterways (oceans, large lakes, rivers) are considered navigable 80km either side of a human settlement within 4 km of the coast. (80 km is the approximate distance a vessel can travel and return in daylight hours). Rivers also had to be > 2m deep. | Indirect | "*wild mushroom collection, forage for stall fed animals, orchid collection, rattan harvesting, etc. other plants accidientally removed/killed as a result of methods/approach used to harvest a target species, etc*." Similar to hunting, the gathering of terrestrial plants is strongly governed by human presence and accessibility of an areas [11, 13]. Therefore, we used roads and navigable waterways as proxies, along with human presence in an area (the population density layer in the human footprint was thresholded into a present/absent binary score; see methods). |
|  |  | Population density | Human population density was mapped using the Gridded population of the world dataset, which provides a 4x4 km dataset globally. (CIESEN 2005) | Indirect |  |
|  |  | Roads | gROADS data was used to map major roadways and contains many but not all minor roadways (CIESEN 2013) | Indirect |  |
| 6. Human intrusions & disturbance (definition: threats from human activities that alter, destroy and disturb habitats and species associated with non-consumptive uses of biological resources) | 6.1 Recreational activities (definition: people spending time in nature or traveling in vehicles outside of established transport corridors, usually for recreational reasons) | Electric infrastructure (nightlights) | Nightlights data (Elvidge et al 2001) was used to map rural housing and suburban areas. This was done by setting brightness thresholds as described in [14]. | Indirect | "*off-road vehicles, motorboats, motorcycles, jet-skis, snowmobiles, ultralight planes, dive boats, whale watching, mountain bikes, hikers, cross-country skiers, hangliders, birdwatchers, scuba divers, pets brought into recreation areas, temporary campsites, caving, rock-climbing, etc*." Monitoring human recreational activity and impacts at a global scale is incredibly challenging. However, Levin, Kark (15) have demonstrated the utility of nightlights for monitoring recreational activity within protected areas globally, and found a correlation between visitation and human population density. Therefore, we decided to use nightlights and population density as a proxies for recreational impacts on species. |
|  |  | Population density | Human population density was mapped using the Gridded population of the world dataset, which provides a 4x4 km dataset globally. (CIESEN 2005) | Indirect |  |
|  | 6.3 Work & other activities (definition: people spending time in or traveling in natural environments for reasons other than recreation or military activities) | Electric infrastructure (nightlights) | Nightlights data (Elvidge et al 2001) was used to map rural housing and suburban areas. This was done by setting brightness thresholds as described in (Venter 2016). | Indirect | "*law enforcement, drug smugglers, illegal immigrants, species research, vandalism, etc*." No globally standardised data exists for these examples of pollution. We assume that nightlights (beyond urban areas) and population density proxy to an extent for these. |
|  |  | Population density | Human population density was mapped using the Gridded population of the world dataset, which provides a 4x4 km dataset globally. (CIESEN 2005) | Indirect |  |
| 8. Invasive & other problematic species, genes & diseases (definition: threats from non-native and native plants, animals, pathogens/microbes, or genetic materials that have or are predicted to have harmful effects on biodiversity following their introduction, spread and/or increase in abundance) | 8.1 Invasive non-native / alien species / diseases (definition: harmful plants, animals, pathogens and other microbes not originally found within the ecosystem(s) in question and directly or indirectly introduced and spread into it by human activities) | Population density | Human population density was mapped using the Gridded population of the world dataset, which provides a 4x4 km dataset globally. (CIESEN 2005) | Indirect | "*feral domesticated cattle, household pets, zebra mussels, Dutch elm disease or chestnut blight, Miconia tree, introduction of species for biocontrol, chytrid fungus affecting amphibians, etc*." There is good evidence suggesting the spread of invasive species is closely linked to human pressure, access to wild areas and human transportation corridors, especially paved roads [16-18], hence the choice of population density and roads as proxies. |
|  |  | Roads | gROADS data was used to map major roadways and contains many but not all minor roadways (CIESEN 2013) | Indirect |  |
| 9. Pollution (definition: threats from introduction of exotic and/or excess materials or energy from point and nonpoint sources) | 9.1 Domestic and urban waste water (definition: Water-borne sewage and non-point runoff from housing and urban areas that include nutrients, toxic chemicals and/or sediments) | Population density | Human population density was mapped using the Gridded population of the world dataset, which provides a 4x4 km dataset globally. (CIESEN 2005) | Indirect | "*discharge from municipal waste treatment plants, leaking septic systems, untreated sewage, outhouses, etc*." To our knowledge no spatial globally standardised data exists for these threats, but we beieve it is safe to assume that population density is a good proxy for waste, sewage and chemical runnoff. |
|  |  | Built environments | Built environments were mapped in the human footprint using data on nightlights (Elvidge et al. 2001). The data was thresholded at a brightness consistent with a city based on global analyses (Small et al. 2011) and visual validation with Landsat imagery of 10 cities globally. | Indirect | "*oil or sediment from roads, fertilizers and pesticides from lawns and golf-courses, road salt, etc*." The human footprint directly maps urban areas, where urban water run off originates. |
|  | 9.3 Agriculture & forestry effluents (definition: water-borne pollutants from agricultural, silivicultural, and aquaculture systems that include nutrients, toxic chemicals and/or sediments including the effects of these pollutants on the site where they are applied) | Crop lands | Intensive agriculture was mapped in the human footprint using data obtained from GlobCover. The human footprint does not capture small scale shifting agriculture due to limitiations in the ability of remote sensing to corrrectly identify this land use. | Indirect | "*nutrient loading from fertilizer run-off, manure from feedlots, nutrients from aquaculture, etc*." The human footprint directly maps croplands where fertilizer etc is applied. |
|  | 9.4 Garbage & solid waste (definition: rubbish and other solid materials including those that entangle wildlife) | Built environments | Built environments were mapped in the human footprint using data on nightlights (Elvidge et al. 2001). The data was thresholded at a brightness consistent with a city based on global analyses (Small et al. 2011) and visual validation with Landsat imagery of 10 cities globally. | Indirect | "*municipal waste, litter from cars, flotsam & jetsam from recreational boats, waste that entangles wildlife, construction debris, etc*." Garbage and solid waste primarily originates in urban areas and is often located on their periphery. The human footprint does not directly map waste, but it maps the urban areas it comes from. |
|  | 9.6 Excess energy (definition: inputs of heat, sound, or light that disturb wildlife or ecosystems) | Electric infrastructure (nightlights) | Nightlights data (Elvidge et al 2001) was used to map rural housing and suburban areas. This was done by setting brightness thresholds as described in Venter 2016. | Directly mapped | "*lamps attracting insects, beach lights disorienting turtles, etc*" The human footprint directly maps light pollution (note the urban areas layer was developed from nightlights data. Sound pollution will likely be highly correlated with urban areas. |
|  |  | Built environments | Built environments were mapped in the human footprint using data on nightlights (Elvidge et al. 2001). The data was thresholded at a brightness consistent with a city based on global analyses (Small et al. 2011) and visual validation with Landsat imagery of 10 cities globally. | directly mapped |  |

1. Elvidge CD, Imhoff ML, Baugh KE, Hobson VR, Nelson I, Safran J, et al. Night-time lights of the world: 1994–1995. ISPRS Journal of Photogrammetry and Remote Sensing. 2001;56(2):81-99. doi: <http://dx.doi.org/10.1016/S0924-2716(01)00040-5>.

2. Small C, Elvidge CD, Balk D, Montgomery M. Spatial scaling of stable night lights. Remote Sensing of Environment. 2011;115(2):269-80. doi: <http://dx.doi.org/10.1016/j.rse.2010.08.021>.

3. Ramankutty N, Evan AT, Monfreda C, Foley JA. Farming the planet: 1. Geographic distribution of global agricultural lands in the year 2000. Global Biogeochemical Cycles. 2008;22(1):n/a-n/a. doi: 10.1029/2007gb002952.

4. NIMA. National Imagery and Mapping Agency. Vector Map Level 0 (VMAP0). (Washington DC). 1997.

5. CIESEN. Global roads open access dataset, v1 (gROADSv1) (NASA Socio-economic Data and Applications Center SEDAC). 2013.

6. Benítez-López A, Alkemade R, Schipper AM, Ingram DJ, Verweij PA, Eikelboom JAJ, et al. The impact of hunting on tropical mammal and bird populations. Science. 2017;356(6334):180-3. doi: 10.1126/science.aaj1891.

7. Symes WS, Edwards DP, Miettinen J, Rheindt FE, Carrasco R. Combined impacts of deforestation and wildlife trade on tropical biodiversity are severely underestimated. Nature Communications. 2018;9:4052.

8. Kilgo JC, Labisky RF, Fritzen DE. Influences of Hunting on the Behavior of White-Tailed Deer: Implications for Conservation of the Florida Panther. Conservation Biology. 1998;12(6):1359-64. doi: doi:10.1111/j.1523-1739.1998.97223.x.

9. Laurance WF, CROES BM, TCHIGNOUMBA L, LAHM SA, ALONSO A, LEE ME, et al. Impacts of Roads and Hunting on Central African Rainforest Mammals. Conservation Biology. 2006;20(4):1251-61. doi: doi:10.1111/j.1523-1739.2006.00420.x.

10. Stillfried M, Belant JL, Svoboda NJ, Beyer DE, Kramer-Schadt S. When top predators become prey: Black bears alter movement behaviour in response to hunting pressure. Behavioural Processes. 2015;120:30-9. doi: <https://doi.org/10.1016/j.beproc.2015.08.003>.

11. Trombulak SC, Frissell CA. Review of Ecological Effects of Roads on Terrestrial and Aquatic Communities. Conservation Biology. 2000;14(1):18-30. doi: doi:10.1046/j.1523-1739.2000.99084.x.

12. CIESEN. Gridded Population of the world, v3 Centre for International Earth Science Network, Columbia University. 2005.

13. Alexander SJ, Fight RD. Managing Access to Nontimber Forest Products. In: Monserud RA, Haynes RW, Johnson AC, editors. Compatible Forest Management. Dordrecht: Springer Netherlands; 2003. p. 383-400.

14. Venter O, Sanderson EW, Magrach A, Allan JR, Beher J, Jones KR, et al. Global terrestrial Human Footprint maps for 1993 and 2009. Scientific Data. 2016;3:160067. doi: 10.1038/sdata.2016.67.

15. Levin N, Kark S, Crandall D. Where have all the people gone? Enhancing global conservation using night lights and social media. Ecological Applications. 2015;25(8):2153-67. doi: doi:10.1890/15-0113.1.

16. Hulme PE. Trade, transport and trouble: managing invasive species pathways in an era of globalization. Journal of Applied Ecology. 2009;46(1):10-8. doi: 10.1111/j.1365-2664.2008.01600.x.

17. Meunier G, Lavoie C. Roads as Corridors for Invasive Plant Species: New Evidence from Smooth Bedstraw (Galium mollugo). Invasive Plant Science and Management. 2012;5(1):92-100. doi: 10.1614/IPSM-D-11-00049.1.

18. Pyšek P, Jarošík V, Hulme PE, Kühn I, Wild J, Arianoutsou M, et al. Disentangling the role of environmental and human pressures on biological invasions across Europe. Proceedings of the National Academy of Sciences. 2010;107(27):12157-62. doi: 10.1073/pnas.1002314107.
