# Supplementary material for: Hotspots of human impact on threatened terrestrial vertebrates
Source: PLoS Biol. 2019 Mar 12;17(3):e3000158. doi: 10.1371/journal.pbio.3000158 (PMC6413901; doi:10.1371/journal.pbio.3000158)
Supplement: S2 Table — (DOCX) [file pbio.3000158.s008.docx]

Table S2. The eight mapped human pressures, the number of sensitive species they impact, the area in which these impacts are occurring, and the proportion of Earth's terrestrial area where these impacts are occurring.

|  | | | |
| --- | --- | --- | --- |
| Human Pressures (threats) | Number of sensitive species Impacted | Area where sensitive species are impacted (km2) | Proportion of Earth's terrestrial area where impacts are occurring (%) |
| Roads | 2832 | 103,873,500 | 72 |
| Crop lands | 3834 | 65,234,800 | 45 |
| Pasture lands | 1642 | 58,348,800 | 40 |
| Built Environments | 1565 | 27,027,900 | 18 |
| Nightlights | 2049 | 50,246,100 | 35 |
| Navigable Waterways | 1531 | 35,118,000 | 24 |
| Railways | 405 | 27,903,600 | 19 |
| Population Density | 2856 | 82,519,200 | 57 |
