# Supplementary material for: Hotspots of human impact on threatened terrestrial vertebrates
Source: PLoS Biol. 2019 Mar 12;17(3):e3000158. doi: 10.1371/journal.pbio.3000158 (PMC6413901; doi:10.1371/journal.pbio.3000158)
Supplement: S3 Table — (DOCX) [file pbio.3000158.s009.docx]

| **Highly impacted Countries** | **Mean number of impacted species per grid cell** | **Mean number of unimpacted species per grid cell** |
| --- | --- | --- |
| Malaysia | 125.2 | 0.6 |
| Brunei | 124.3 | 11.8 |
| Singapore | 112.0 | 10.0 |
| Indonesia | 68.6 | 0.5 |
| Myanmar | 51.6 | 0.8 |
| Ecuador | 49.9 | 0.9 |
| Cambodia | 47.2 | 0.5 |
| Thailand | 46.6 | 0.1 |
| Laos | 45.7 | 0.5 |
| Bhutan | 45.0 | 0.1 |
| **Highly unimpacted Countries** |  |  |
| Liberia | 25.6 | 23.1 |
| Suriname | 19.4 | 13.2 |
| Brunei | 124.3 | 11.8 |
| Singapore | 112.0 | 10.0 |
| Sierra Leone | 25.4 | 9.3 |
| Ivory coast | 25.3 | 5.8 |
| Guyana | 30.1 | 5.7 |
| Madagascar | 19.3 | 5.6 |
| The Bahamas | 10.0 | 5.1 |
| Peru | 27.5 | 4.9 |
