# Supplementary material for: Hotspots of human impact on threatened terrestrial vertebrates
Source: PLoS Biol. 2019 Mar 12;17(3):e3000158. doi: 10.1371/journal.pbio.3000158 (PMC6413901; doi:10.1371/journal.pbio.3000158)
Supplement: S4 Table — (DOCX) [file pbio.3000158.s010.docx]

| **Biome Name** | **Average number of species impacted per grid cell** | **Average number of species not impacted per grid cell** | **Average proportion of species impacted per grid cell** |
| --- | --- | --- | --- |
| Tropical and subtropical moist broadleaf forests | 34.8 | 5.2 | 89.5 |
| Tundra | 1.2 | 2.5 | 48.6 |
| Boreal forests taiga | 3.5 | 2.3 | 60.3 |
| Deserts and xeric shrublands | 10.1 | 1.8 | 83.1 |
| Mangrove | 35.0 | 1.8 | 93.4 |
| Montane grasslands and savannas | 16.5 | 1.5 | 93.2 |
| Temperate coniferous forests | 11.6 | 1.2 | 78.5 |
| Tropical and subtropical grasslands savannas | 19.6 | 0.7 | 95.9 |
| Tropical and subtropical coniferous forests | 21.7 | 0.7 | 95.9 |
| Temperate broadleaf and mixed forests | 14.7 | 0.6 | 94.7 |
| Flooded grasslands and savannas | 25.0 | 0.6 | 97.5 |
| Tropical and subtropical dry broadleaf forests | 29.8 | 0.5 | 98.3 |
| Mediterranean forests woodlands and scrub | 13.9 | 0.3 | 97.3 |
| Temperate grasslands savannas and shrublands | 13.1 | 0.2 | 97.8 |
