# Supplementary material for: Hotspots of human impact on threatened terrestrial vertebrates
Source: PLoS Biol. 2019 Mar 12;17(3):e3000158. doi: 10.1371/journal.pbio.3000158 (PMC6413901; doi:10.1371/journal.pbio.3000158)
Supplement: S5 Table — (DOCX) [file pbio.3000158.s011.docx]

| **Ecoregion Name** | **Average number of species impacted per grid cell** | **Average number of species not impacted per grid cell** |
| --- | --- | --- |
| Admiralty Islands lowland rain forests | 14.0 | 1.0 |
| Aegean and Western Turkey sclerophyllous and mixed forests | 15.3 | 0.2 |
| Afghan Mountains semi-desert | 15.5 | 0.0 |
| Al Hajar montane woodlands | 11.4 | 0.8 |
| Alai-Western Tian Shan steppe | 21.0 | 0.1 |
| Alashan Plateau semi-desert | 10.0 | 2.4 |
| Alaska-St. Elias Range tundra | 0.9 | 2.9 |
| Alaska Peninsula montane taiga | 3.2 | 5.5 |
| Albany thickets | 24.1 | 1.3 |
| Alberta-British Columbia foothills forests | 2.5 | 1.5 |
| Alberta Mountain forests | 1.4 | 1.6 |
| Albertine Rift montane forests | 34.4 | 4.7 |
| Aleutian Islands tundra | 3.7 | 6.5 |
| Allegheny Highlands forests | 9.7 | 2.1 |
| Alps conifer and mixed forests | 11.2 | 0.0 |
| Altai alpine meadow and tundra | 18.4 | 0.3 |
| Altai montane forest and forest steppe | 17.2 | 1.0 |
| Altai steppe and semi-desert | 17.5 | 0.0 |
| Alto Parana Atlantic forests | 45.9 | 0.1 |
| Amazon-Orinoco-Southern Caribbean mangroves | 31.6 | 1.8 |
| Amur meadow steppe | 21.7 | 0.5 |
| Anatolian conifer and deciduous mixed forests | 17.9 | 0.1 |
| Andaman Islands rain forests | 17.6 | 3.6 |
| Angolan Miombo woodlands | 15.6 | 0.9 |
| Angolan montane forest-grassland mosaic | 14.5 | 0.4 |
| Angolan Mopane woodlands | 22.2 | 0.7 |
| Angolan scarp savanna and woodlands | 14.0 | 0.8 |
| Appalachian-Blue Ridge forests | 13.2 | 1.4 |
| Appalachian mixed mesophytic forests | 13.2 | 1.3 |
| Appenine deciduous montane forests | 13.5 | 0.1 |
| Apure-Villavicencio dry forests | 27.2 | 0.3 |
| Arabian Desert and East Sahero-Arabian xeric shrublands | 8.6 | 1.1 |
| Arabian Peninsula coastal fog desert | 13.4 | 0.9 |
| Araucaria moist forests | 48.6 | 0.1 |
| Araya and Paria xeric scrub | 33.9 | 0.4 |
| Arctic coastal tundra | 1.5 | 3.4 |
| Arctic desert | 0.0 | 3.3 |
| Arctic foothills tundra | 1.0 | 2.3 |
| Arizona Mountains forests | 5.6 | 0.7 |
| Arnhem Land tropical savanna | 6.0 | 3.9 |
| Atacama desert | 5.9 | 1.4 |
| Atlantic Coast restingas | 47.7 | 0.3 |
| Atlantic coastal desert | 9.6 | 2.7 |
| Atlantic coastal pine barrens | 14.1 | 1.0 |
| Atlantic dry forests | 24.4 | 0.0 |
| Atlantic Equatorial coastal forests | 17.9 | 4.1 |
| Atlantic mixed forests | 8.3 | 0.3 |
| Australian Alps montane grasslands | 15.1 | 0.4 |
| Azerbaijan shrub desert and steppe | 21.7 | 0.0 |
| Azores temperate mixed forests | 11.0 | 0.0 |
| Badghyz and Karabil semi-desert | 21.4 | 0.0 |
| Baffin coastal tundra | 1.3 | 2.5 |
| Bahamian-Antillean mangroves | 20.9 | 3.2 |
| Bahamian pine mosaic | 11.8 | 2.8 |
| Bahia coastal forests | 53.7 | 0.1 |
| Bahia interior forests | 47.6 | 0.0 |
| Baja California desert | 8.4 | 1.6 |
| Bajio dry forests | 18.1 | 0.2 |
| Balkan mixed forests | 19.2 | 0.0 |
| Balsas dry forests | 13.4 | 0.9 |
| Baltic mixed forests | 7.0 | 0.0 |
| Baluchistan xeric woodlands | 19.7 | 0.4 |
| Banda Sea Islands moist deciduous forests | 15.5 | 0.6 |
| Belizian pine forests | 29.3 | 1.0 |
| Beni savanna | 38.7 | 1.5 |
| Bering tundra | 2.5 | 3.4 |
| Beringia lowland tundra | 2.2 | 3.6 |
| Beringia upland tundra | 2.0 | 2.5 |
| Biak-Numfoor rain forests | 20.5 | 0.5 |
| Blue Mountains forests | 1.6 | 0.5 |
| Bohai Sea saline meadow | 21.4 | 0.1 |
| Bolivian montane dry forests | 20.0 | 0.0 |
| Bolivian Yungas | 45.6 | 1.3 |
| Borneo lowland rain forests | 112.8 | 0.5 |
| Borneo montane rain forests | 99.7 | 1.1 |
| Borneo peat swamp forests | 113.7 | 1.4 |
| Brahmaputra Valley semi-evergreen forests | 65.9 | 0.0 |
| Brigalow tropical savanna | 8.0 | 0.6 |
| British Columbia mainland coastal forests | 2.9 | 4.0 |
| Brooks-British Range tundra | 0.3 | 2.9 |
| Buru rain forests | 21.2 | 0.5 |
| Caatinga | 16.8 | 0.0 |
| Caatinga Enclaves moist forests | 10.0 | 0.0 |
| Caledon conifer forests | 2.6 | 0.1 |
| California Central Valley grasslands | 8.9 | 0.0 |
| California coastal sage and chaparral | 11.3 | 1.0 |
| California interior chaparral and woodlands | 11.4 | 0.1 |
| California montane chaparral and woodlands | 9.8 | 0.5 |
| Cameroonian Highlands forests | 30.2 | 6.6 |
| Campos Rupestres montane savanna | 50.3 | 0.3 |
| Canadian Aspen forests and parklands | 5.4 | 0.4 |
| Canary Islands dry woodlands and forests | 7.8 | 0.0 |
| Cantabrian mixed forests | 15.2 | 0.4 |
| Cantebury-Otago tussock grasslands | 16.1 | 0.5 |
| Cape Verde Islands dry forests | 9.3 | 0.0 |
| Cape York Peninsula tropical savanna | 5.9 | 1.4 |
| Caqueta moist forests | 16.2 | 15.7 |
| Cardamom Mountains rain forests | 48.1 | 0.8 |
| Caribbean shrublands | 9.8 | 0.3 |
| Carnarvon xeric shrublands | 3.9 | 0.7 |
| Carolines tropical moist forests | 15.0 | 0.0 |
| Carpathian montane forests | 13.2 | 0.0 |
| Carpentaria tropical savanna | 4.4 | 1.2 |
| Cascade Mountains leeward forests | 1.8 | 1.5 |
| Caspian Hyrcanian mixed forests | 26.5 | 0.0 |
| Caspian lowland desert | 16.8 | 0.4 |
| Catatumbo moist forests | 25.8 | 0.5 |
| Cauca Valley dry forests | 41.2 | 0.0 |
| Cauca Valley montane forests | 47.6 | 0.1 |
| Caucasus mixed forests | 25.5 | 0.0 |
| Celtic broadleaf forests | 4.0 | 0.0 |
| Central Afghan Mountains xeric woodlands | 16.2 | 0.0 |
| Central African mangroves | 18.9 | 2.7 |
| Central American Atlantic moist forests | 28.1 | 1.0 |
| Central American dry forests | 18.8 | 0.0 |
| Central American montane forests | 33.1 | 0.6 |
| Central American pine-oak forests | 24.1 | 0.4 |
| Central Anatolian steppe | 18.6 | 0.0 |
| Central Anatolian steppe and woodlands | 19.1 | 0.0 |
| Central and Southern Cascades forests | 4.4 | 1.1 |
| Central and Southern mixed grasslands | 12.1 | 0.1 |
| Central Andean dry puna | 10.3 | 0.8 |
| Central Andean puna | 12.0 | 0.7 |
| Central Andean wet puna | 19.9 | 0.5 |
| Central Asian northern desert | 18.5 | 0.1 |
| Central Asian riparian woodlands | 19.2 | 0.0 |
| Central Asian southern desert | 20.6 | 0.1 |
| Central British Columbia Mountain forests | 0.7 | 2.0 |
| Central Canadian Shield forests | 1.5 | 2.7 |
| Central China loess plateau mixed forests | 17.3 | 0.0 |
| Central Congolian lowland forests | 16.0 | 0.1 |
| Central Deccan Plateau dry deciduous forests | 28.0 | 0.0 |
| Central European mixed forests | 10.6 | 0.0 |
| Central forest-grasslands transition | 14.7 | 0.0 |
| Central Indochina dry forests | 37.5 | 0.1 |
| Central Korean deciduous forests | 18.6 | 1.1 |
| Central Mexican matorral | 14.0 | 0.3 |
| Central Pacific coastal forests | 8.0 | 2.0 |
| Central Persian desert basins | 17.4 | 0.6 |
| Central Range montane rain forests | 11.1 | 0.1 |
| Central Range sub-alpine grasslands | 11.6 | 0.0 |
| Central Ranges xeric scrub | 3.9 | 0.6 |
| Central tall grasslands | 11.4 | 0.0 |
| Central Tibetan Plateau alpine steppe | 7.9 | 3.9 |
| Central U.S. hardwood forests | 14.8 | 0.4 |
| Central Zambezian Miombo woodlands | 23.2 | 0.3 |
| Cerrado | 36.9 | 0.4 |
| Changbai Mountains mixed forests | 17.6 | 0.5 |
| Changjiang Plain evergreen forests | 23.1 | 0.0 |
| Chao Phraya freshwater swamp forests | 33.8 | 0.0 |
| Chao Phraya lowland moist deciduous forests | 47.0 | 0.0 |
| Cherskii-Kolyma mountain tundra | 2.2 | 2.4 |
| Chhota-Nagpur dry deciduous forests | 29.7 | 0.0 |
| Chiapas Depression dry forests | 24.9 | 0.1 |
| Chiapas montane forests | 33.4 | 0.8 |
| Chihuahuan desert | 8.3 | 0.9 |
| Chilean matorral | 10.6 | 0.1 |
| Chimalapas montane forests | 32.5 | 4.0 |
| Chin Hills-Arakan Yoma montane forests | 43.5 | 0.1 |
| Chiquitano dry forests | 36.9 | 1.2 |
| Choco-Darien moist forests | 43.2 | 2.9 |
| Chukchi Peninsula tundra | 1.6 | 1.7 |
| Colorado Plateau shrublands | 4.7 | 0.6 |
| Colorado Rockies forests | 6.0 | 0.3 |
| Comoros forests | 14.0 | 0.0 |
| Cook Inlet taiga | 3.7 | 2.3 |
| Cook Islands tropical moist forests | 18.0 | 2.0 |
| Coolgardie woodlands | 1.5 | 0.1 |
| Copper Plateau taiga | 1.7 | 1.4 |
| Cordillera Central piramo | 30.9 | 1.8 |
| Cordillera de Merida píramo | 46.0 | 1.0 |
| Cordillera La Costa montane forests | 34.1 | 2.7 |
| Cordillera Oriental montane forests | 44.4 | 0.7 |
| Corsican montane broadleaf and mixed forests | 12.0 | 0.0 |
| Costa Rican seasonal moist forests | 31.9 | 0.0 |
| Crete Mediterranean forests | 11.5 | 0.0 |
| Crimean Submediterranean forest complex | 20.9 | 0.0 |
| Cross-Niger transition forests | 15.1 | 0.3 |
| Cross-Sanaga-Bioko coastal forests | 26.5 | 7.4 |
| Cuban cactus scrub | 28.0 | 0.0 |
| Cuban dry forests | 29.3 | 0.3 |
| Cuban moist forests | 34.3 | 0.3 |
| Cuban pine forests | 34.7 | 0.3 |
| Cuban wetlands | 30.4 | 0.9 |
| Cyprus Mediterranean forests | 12.5 | 0.0 |
| Da Hinggan-Dzhagdy Mountains conifer forests | 15.4 | 1.8 |
| Daba Mountains evergreen forests | 25.7 | 0.0 |
| Daurian forest steppe | 18.2 | 0.3 |
| Davis Highlands tundra | 0.5 | 2.8 |
| Deccan thorn scrub forests | 27.7 | 0.0 |
| Dinaric Mountains mixed forests | 16.0 | 0.0 |
| Drakensberg alti-montane grasslands and woodlands | 20.1 | 0.2 |
| Drakensberg montane grasslands, woodlands and forests | 25.9 | 0.2 |
| Dry Chaco | 17.9 | 0.6 |
| East Afghan montane conifer forests | 17.2 | 0.0 |
| East African halophytics | 38.3 | 0.0 |
| East African mangroves | 28.5 | 0.9 |
| East African montane forests | 37.8 | 0.0 |
| East African montane moorlands | 42.8 | 0.6 |
| East Central Texas forests | 16.2 | 0.1 |
| East Deccan dry-evergreen forests | 22.6 | 0.0 |
| East European forest steppe | 15.2 | 0.0 |
| East Saharan montane xeric woodlands | 8.7 | 4.2 |
| East Siberian taiga | 4.0 | 2.5 |
| East Sudanian savanna | 20.5 | 0.8 |
| Eastern Anatolian deciduous forests | 21.2 | 0.1 |
| Eastern Anatolian montane steppe | 24.1 | 0.0 |
| Eastern Arc forests | 39.9 | 1.6 |
| Eastern Australia mulga shrublands | 4.4 | 0.9 |
| Eastern Australian temperate forests | 23.9 | 0.8 |
| Eastern Canadian forests | 2.4 | 2.3 |
| Eastern Canadian Shield taiga | 0.6 | 3.5 |
| Eastern Cascades forests | 4.4 | 0.9 |
| Eastern Congolian swamp forests | 15.9 | 0.3 |
| Eastern Cordillera real montane forests | 60.2 | 1.3 |
| Eastern forest-boreal transition | 3.3 | 3.2 |
| Eastern Gobi desert steppe | 13.1 | 0.2 |
| Eastern Great Lakes lowland forests | 8.2 | 1.2 |
| Eastern Guinean forests | 26.5 | 7.5 |
| Eastern highlands moist deciduous forests | 31.1 | 0.0 |
| Eastern Himalayan alpine shrub and meadows | 26.0 | 0.9 |
| Eastern Himalayan broadleaf forests | 51.2 | 1.3 |
| Eastern Himalayan subalpine conifer forests | 41.0 | 0.7 |
| Eastern Java-Bali montane rain forests | 35.5 | 0.0 |
| Eastern Java-Bali rain forests | 30.7 | 0.0 |
| Eastern Mediterranean conifer-sclerophyllous-broadleaf forests | 20.3 | 0.1 |
| Eastern Miombo woodlands | 20.3 | 0.6 |
| Eastern Panamanian montane forests | 31.0 | 1.5 |
| Eastern Zimbabwe montane forest-grassland mosaic | 27.6 | 0.3 |
| Ecuadorian dry forests | 33.9 | 0.3 |
| Edwards Plateau savanna | 11.4 | 0.3 |
| Einasleigh upland savanna | 6.7 | 0.2 |
| Elburz Range forest steppe | 24.5 | 0.0 |
| Emin Valley steppe | 17.0 | 0.0 |
| English Lowlands beech forests | 5.8 | 0.1 |
| Enriquillo wetlands | 33.0 | 0.0 |
| Eritrean coastal desert | 18.0 | 0.0 |
| Esperance mallee | 7.0 | 0.4 |
| Espinal | 14.4 | 0.3 |
| Ethiopian montane forests | 25.4 | 0.2 |
| Ethiopian montane grasslands and woodlands | 27.2 | 0.4 |
| Ethiopian montane moorlands | 31.1 | 0.6 |
| Ethiopian xeric grasslands and shrublands | 20.9 | 0.4 |
| Etosha Pan halophytics | 26.8 | 0.3 |
| Euxine-Colchic broadleaf forests | 18.5 | 0.0 |
| Everglades | 13.5 | 1.8 |
| Eyre and York mallee | 9.8 | 0.6 |
| Fiji tropical dry forests | 23.3 | 0.3 |
| Fiji tropical moist forests | 18.6 | 0.4 |
| Fiordland temperate forests | 32.2 | 6.1 |
| Flint Hills tall grasslands | 14.7 | 0.1 |
| Florida sand pine scrub | 18.4 | 1.0 |
| Fraser Plateau and Basin complex | 0.6 | 1.4 |
| Ghorat-Hazarajat alpine meadow | 15.9 | 0.0 |
| Gibson desert | 1.6 | 2.4 |
| Gissaro-Alai open woodlands | 21.0 | 0.0 |
| Goadavari-Krishna mangroves | 26.3 | 0.0 |
| Gobi Lakes Valley desert steppe | 16.4 | 0.2 |
| Great Basin montane forests | 2.8 | 0.3 |
| Great Basin shrub steppe | 3.3 | 0.4 |
| Great Lakes Basin desert steppe | 18.7 | 1.2 |
| Great Sandy-Tanami desert | 1.9 | 2.1 |
| Great Victoria desert | 0.7 | 0.9 |
| Greater Negros-Panay rain forests | 33.1 | 0.1 |
| Guajira-Barranquilla xeric scrub | 23.1 | 0.5 |
| Guayaquil flooded grasslands | 34.7 | 0.0 |
| Guianan freshwater swamp forests | 32.4 | 6.3 |
| Guianan Highlands moist forests | 18.8 | 15.8 |
| Guianan moist forests | 23.8 | 13.2 |
| Guianan piedmont and lowland moist forests | 14.5 | 18.6 |
| Guianan savanna | 32.8 | 3.9 |
| Guinean forest-savanna mosaic | 17.2 | 0.5 |
| Guinean mangroves | 23.3 | 6.0 |
| Guinean montane forests | 22.1 | 5.5 |
| Guizhou Plateau broadleaf and mixed forests | 26.1 | 0.0 |
| Gulf of California xeric scrub | 11.9 | 2.6 |
| Gulf of Oman desert and semi-desert | 12.6 | 0.7 |
| Gulf of St. Lawrence lowland forests | 6.1 | 1.3 |
| Gurupa varzea | 35.2 | 16.8 |
| Hainan Island monsoon rain forests | 29.9 | 0.6 |
| Halmahera rain forests | 20.2 | 1.3 |
| Hawaii tropical dry forests | 25.0 | 0.0 |
| Hawaii tropical high shrublands | 13.0 | 0.0 |
| Hawaii tropical low shrublands | 27.5 | 0.0 |
| Hawaii tropical moist forests | 22.7 | 0.3 |
| Helanshan montane conifer forests | 17.1 | 0.9 |
| Hengduan Mountains subalpine conifer forests | 30.3 | 0.0 |
| High Arctic tundra | 0.1 | 2.5 |
| High Monte | 7.4 | 0.3 |
| Highveld grasslands | 23.7 | 0.5 |
| Himalayan subtropical broadleaf forests | 53.7 | 0.3 |
| Himalayan subtropical pine forests | 42.1 | 0.1 |
| Hindu Kush alpine meadow | 18.0 | 0.0 |
| Hispaniolan dry forests | 25.9 | 0.6 |
| Hispaniolan moist forests | 25.2 | 0.4 |
| Hispaniolan pine forests | 33.1 | 0.8 |
| Hobyo grasslands and shrublands | 10.3 | 0.7 |
| Hokkaido deciduous forests | 14.5 | 0.0 |
| Hokkaido montane conifer forests | 12.5 | 0.0 |
| Honshu alpine conifer forests | 17.0 | 0.0 |
| Huang He Plain mixed forests | 18.5 | 0.0 |
| Humid Chaco | 24.7 | 0.2 |
| Humid Pampas | 14.9 | 0.0 |
| Huon Peninsula montane rain forests | 14.9 | 0.1 |
| Iberian conifer forests | 18.3 | 0.6 |
| Iberian sclerophyllous and semi-deciduous forests | 18.4 | 0.5 |
| Iceland boreal birch forests and alpine tundra | 1.0 | 0.6 |
| Illyrian deciduous forests | 17.0 | 0.0 |
| Indochina mangroves | 43.3 | 0.1 |
| Indus River Delta-Arabian Sea mangroves | 34.7 | 1.0 |
| Indus Valley desert | 26.8 | 0.0 |
| Inner Niger Delta flooded savanna | 22.1 | 0.0 |
| Interior Alaska-Yukon lowland taiga | 0.9 | 2.5 |
| Interior Yukon-Alaska alpine tundra | 0.9 | 2.4 |
| Iquitos varzea | 31.7 | 6.1 |
| Irrawaddy dry forests | 51.0 | 0.0 |
| Irrawaddy freshwater swamp forests | 38.8 | 0.1 |
| Irrawaddy moist deciduous forests | 54.3 | 0.0 |
| Isthmian-Atlantic moist forests | 32.6 | 0.7 |
| Isthmian-Pacific moist forests | 30.0 | 0.2 |
| Italian sclerophyllous and semi-deciduous forests | 13.5 | 0.2 |
| Itigi-Sumbu thicket | 28.8 | 0.0 |
| Jalisco dry forests | 22.0 | 1.3 |
| Jamaican dry forests | 28.0 | 0.0 |
| Jamaican moist forests | 27.4 | 0.0 |
| Japura-Solimoes-Negro moist forests | 13.3 | 17.1 |
| Jarrah-Karri forest and shrublands | 20.7 | 1.9 |
| Jian Nan subtropical evergreen forests | 28.4 | 0.0 |
| Jos Plateau forest-grassland mosaic | 18.3 | 0.0 |
| Junggar Basin semi-desert | 14.0 | 0.8 |
| Jurua-Purus moist forests | 15.8 | 18.1 |
| Kalaallit Nunaat high arctic tundra | 0.0 | 2.5 |
| Kalaallit Nunaat low arctic tundra | 1.2 | 3.8 |
| Kalahari Acacia-Baikiaea woodlands | 19.9 | 3.0 |
| Kalahari xeric savanna | 16.9 | 1.2 |
| Kamchatka-Kurile meadows and sparse forests | 6.2 | 2.6 |
| Kamchatka-Kurile taiga | 6.1 | 1.0 |
| Kamchatka Mountain tundra and forest tundra | 4.8 | 3.2 |
| Kaokoveld desert | 14.9 | 5.1 |
| Karakoram-West Tibetan Plateau alpine steppe | 17.2 | 1.8 |
| Kayah-Karen montane rain forests | 53.4 | 0.3 |
| Kazakh forest steppe | 15.7 | 0.1 |
| Kazakh semi-desert | 16.1 | 0.0 |
| Kazakh steppe | 16.4 | 0.0 |
| Kazakh upland | 16.9 | 0.0 |
| Khangai Mountains alpine meadow | 18.5 | 0.3 |
| Khangai Mountains conifer forests | 17.3 | 1.3 |
| Khathiar-Gir dry deciduous forests | 37.0 | 0.0 |
| Kimberly tropical savanna | 6.0 | 1.9 |
| Kinabalu montane alpine meadows | 142.3 | 0.0 |
| Klamath-Siskiyou forests | 4.8 | 1.3 |
| Kola Peninsula tundra | 3.0 | 0.9 |
| Kopet Dag semi-desert | 20.5 | 0.3 |
| Kopet Dag woodlands and forest steppe | 23.7 | 0.2 |
| Kuh Rud and Eastern Iran montane woodlands | 17.2 | 0.2 |
| KwaZulu-Cape coastal forest mosaic | 36.1 | 0.0 |
| La Costa xeric shrublands | 23.9 | 0.3 |
| Lake | 8.2 | 2.5 |
| Lake Chad flooded savanna | 22.9 | 0.0 |
| Lara-Falcon dry forests | 19.8 | 0.3 |
| Leeward Islands moist forests | 14.0 | 0.0 |
| Lesser Antillean dry forests | 13.0 | 0.0 |
| Lesser Sundas deciduous forests | 22.5 | 1.0 |
| Llanos | 22.0 | 1.4 |
| Louisiade Archipelago rain forests | 6.0 | 1.0 |
| Low Arctic tundra | 0.3 | 2.2 |
| Low Monte | 7.6 | 0.5 |
| Lower Gangetic Plains moist deciduous forests | 34.8 | 0.0 |
| Lowland fynbos and renosterveld | 28.8 | 0.4 |
| Luang Prabang montane rain forests | 44.3 | 0.4 |
| Luzon montane rain forests | 52.0 | 0.1 |
| Luzon rain forests | 47.4 | 0.2 |
| Luzon tropical pine forests | 52.6 | 0.0 |
| Madagascar dry deciduous forests | 19.7 | 3.9 |
| Madagascar ericoid thickets | 24.0 | 6.5 |
| Madagascar lowland forests | 27.0 | 11.4 |
| Madagascar mangroves | 25.3 | 6.0 |
| Madagascar spiny thickets | 15.7 | 3.3 |
| Madagascar subhumid forests | 17.3 | 5.2 |
| Madagascar succulent woodlands | 14.7 | 2.8 |
| Madeira-Tapajos moist forests | 28.4 | 14.4 |
| Magdalena-Uraba moist forests | 28.2 | 0.2 |
| Magdalena Valley dry forests | 30.6 | 0.0 |
| Magdalena Valley montane forests | 40.7 | 0.7 |
| Magellanic subpolar forests | 9.1 | 5.5 |
| Malabar Coast moist forests | 38.1 | 0.3 |
| Maldives-Lakshadweep-Chagos Archipelago tropical moist forests | 16.0 | 1.0 |
| Manchurian mixed forests | 18.3 | 1.2 |
| Mandara Plateau mosaic | 21.4 | 0.0 |
| Maputaland-Pondoland bushland and thickets | 28.2 | 0.2 |
| Maputaland coastal forest mosaic | 25.3 | 0.4 |
| Maracaibo dry forests | 22.9 | 0.3 |
| Maraja varzea | 32.3 | 5.0 |
| Maranhao Babacu forests | 20.8 | 0.1 |
| Maranon dry forests | 36.2 | 0.0 |
| Marianas tropical dry forests | 13.0 | 0.0 |
| Marquesas tropical moist forests | 22.0 | 0.0 |
| Masai xeric grasslands and shrublands | 23.8 | 2.5 |
| Mascarene forests | 12.8 | 0.0 |
| Mato Grosso seasonal forests | 26.1 | 9.2 |
| Mediterranean acacia-argania dry woodlands and succulent thickets | 14.1 | 0.3 |
| Mediterranean conifer and mixed forests | 19.7 | 0.1 |
| Mediterranean dry woodlands and steppe | 13.6 | 0.3 |
| Mediterranean High Atlas juniper steppe | 18.4 | 0.0 |
| Mediterranean woodlands and forests | 16.5 | 0.1 |
| Meghalaya subtropical forests | 58.1 | 0.1 |
| Mentawai Islands rain forests | 31.3 | 1.0 |
| Meseta Central matorral | 11.6 | 0.9 |
| Mesoamerican Gulf-Caribbean mangroves | 26.8 | 1.1 |
| Mesopotamian shrub desert | 13.4 | 0.3 |
| Mid-Continental Canadian forests | 2.1 | 1.9 |
| Middle Arctic tundra | 0.3 | 2.7 |
| Middle Atlantic coastal forests | 16.5 | 0.7 |
| Middle East steppe | 16.9 | 0.1 |
| Midwestern Canadian Shield forests | 1.2 | 2.6 |
| Mindanao-Eastern Visayas rain forests | 44.7 | 1.0 |
| Mindanao montane rain forests | 50.9 | 1.4 |
| Mindoro rain forests | 38.1 | 0.4 |
| Miskito pine forests | 21.4 | 1.4 |
| Mississippi lowland forests | 15.8 | 0.2 |
| Mitchell grass downs | 4.1 | 0.2 |
| Mizoram-Manipur-Kachin rain forests | 50.2 | 0.5 |
| Mojave desert | 3.7 | 0.9 |
| Mongolian-Manchurian grassland | 16.5 | 0.0 |
| Montana Valley and Foothill grasslands | 4.3 | 0.2 |
| Montane fynbos and renosterveld | 21.4 | 0.9 |
| Monte Alegre varzea | 33.3 | 9.8 |
| Motagua Valley thornscrub | 38.5 | 0.5 |
| Mount Cameroon and Bioko montane forests | 30.5 | 10.0 |
| Mount Lofty woodlands | 12.2 | 0.5 |
| Murray-Darling woodlands and mallee | 9.5 | 0.1 |
| Muskwa-Slave Lake forests | 1.1 | 2.1 |
| Myanmar Coast mangroves | 53.5 | 1.0 |
| Myanmar coastal rain forests | 49.3 | 0.5 |
| Nama Karoo | 16.4 | 0.9 |
| Namib desert | 11.8 | 7.1 |
| Namibian savanna woodlands | 17.3 | 1.4 |
| Nansei Islands subtropical evergreen forests | 18.0 | 0.0 |
| Napo moist forests | 32.2 | 5.3 |
| Naracoorte woodlands | 14.0 | 0.5 |
| Narmada Valley dry deciduous forests | 35.4 | 0.0 |
| Nebraska Sand Hills mixed grasslands | 7.7 | 0.5 |
| Negro-Branco moist forests | 18.2 | 13.2 |
| Nelson Coast temperate forests | 28.8 | 0.7 |
| Nenjiang River grassland | 18.8 | 0.0 |
| New Britain-New Ireland lowland rain forests | 16.2 | 0.6 |
| New Britain-New Ireland montane rain forests | 15.6 | 0.3 |
| New Caledonia dry forests | 29.3 | 0.3 |
| New Caledonia rain forests | 23.5 | 0.3 |
| New England-Acadian forests | 5.3 | 1.8 |
| New Guinea mangroves | 9.1 | 1.7 |
| Newfoundland Highland forests | 3.1 | 2.4 |
| Nicobar Islands rain forests | 18.0 | 0.5 |
| Niger Delta swamp forests | 15.5 | 1.1 |
| Nigerian lowland forests | 14.7 | 1.3 |
| Nihonkai evergreen forests | 20.1 | 0.0 |
| Nihonkai montane deciduous forests | 16.4 | 0.0 |
| Nile Delta flooded savanna | 11.9 | 0.6 |
| North Atlantic moist mixed forests | 4.6 | 0.4 |
| North Central Rockies forests | 0.6 | 1.1 |
| North Island temperate forests | 17.9 | 0.7 |
| North Saharan steppe and woodlands | 7.5 | 1.8 |
| North Tibetan Plateau-Kunlun Mountains alpine desert | 4.2 | 7.7 |
| North Western Ghats moist deciduous forests | 41.9 | 0.0 |
| North Western Ghats montane rain forests | 45.8 | 0.0 |
| Northeast China Plain deciduous forests | 16.2 | 0.0 |
| Northeast India-Myanmar pine forests | 49.7 | 0.0 |
| Northeast Siberian coastal tundra | 2.1 | 1.7 |
| Northeast Siberian taiga | 2.9 | 1.8 |
| Northeastern Brazil restingas | 19.7 | 0.4 |
| Northeastern coastal forests | 11.0 | 2.0 |
| Northeastern Congolian lowland forests | 18.7 | 2.3 |
| Northeastern Himalayan subalpine conifer forests | 27.1 | 1.0 |
| Northeastern Spain and Southern France Mediterranean forests | 15.5 | 0.6 |
| Northern Acacia-Commiphora bushlands and thickets | 31.2 | 0.9 |
| Northern Anatolian conifer and deciduous forests | 19.6 | 0.0 |
| Northern Andean paramo | 47.2 | 0.6 |
| Northern Annamites rain forests | 53.2 | 1.0 |
| Northern California coastal forests | 14.0 | 2.1 |
| Northern Canadian Shield taiga | 0.3 | 2.1 |
| Northern Congolian forest-savanna mosaic | 16.9 | 1.0 |
| Northern Cordillera forests | 0.8 | 2.2 |
| Northern dry deciduous forests | 30.1 | 0.0 |
| Northern Indochina subtropical forests | 45.5 | 0.3 |
| Northern Khorat Plateau moist deciduous forests | 38.9 | 0.1 |
| Northern Mesoamerican Pacific mangroves | 20.3 | 0.6 |
| Northern mixed grasslands | 7.8 | 0.0 |
| Northern New Guinea lowland rain and freshwater swamp forests | 7.8 | 0.6 |
| Northern New Guinea montane rain forests | 8.1 | 0.5 |
| Northern Pacific coastal forests | 6.3 | 4.9 |
| Northern short grasslands | 7.3 | 0.3 |
| Northern tall grasslands | 8.4 | 0.1 |
| Northern Thailand-Laos moist deciduous forests | 44.1 | 0.2 |
| Northern transitional alpine forests | 0.5 | 1.8 |
| Northern Triangle subtropical forests | 53.9 | 2.0 |
| Northern Triangle temperate forests | 51.4 | 2.6 |
| Northern Vietnam lowland rain forests | 45.8 | 0.0 |
| Northern Zanzibar-Inhambane coastal forest mosaic | 25.6 | 0.2 |
| Northland temperate kauri forests | 26.3 | 0.0 |
| Northwest Iberian montane forests | 17.2 | 0.4 |
| Northwest Russian-Novaya Zemlya tundra | 2.7 | 1.5 |
| Northwest Territories taiga | 0.5 | 2.1 |
| Northwestern Andean montane forests | 63.9 | 0.7 |
| Northwestern Congolian lowland forests | 14.1 | 3.4 |
| Northwestern Himalayan alpine shrub and meadows | 25.1 | 0.8 |
| Northwestern thorn scrub forests | 31.9 | 0.0 |
| Novosibirsk Islands arctic desert | 0.0 | 2.6 |
| Nujiang Langcang Gorge alpine conifer and mixed forests | 32.0 | 0.0 |
| Nullarbor Plains xeric shrublands | 0.4 | 0.1 |
| Oaxacan montane forests | 39.5 | 1.8 |
| Ogilvie-MacKenzie alpine tundra | 0.1 | 2.4 |
| Okanagan dry forests | 0.5 | 1.0 |
| Okhotsk-Manchurian taiga | 6.5 | 8.4 |
| Ordos Plateau steppe | 16.3 | 0.1 |
| Orinoco Delta swamp forests | 29.3 | 3.2 |
| Orinoco wetlands | 27.6 | 0.4 |
| Orissa semi-evergreen forests | 30.3 | 0.0 |
| Ozark Mountain forests | 15.2 | 1.9 |
| Pacific Coastal Mountain icefields and tundra | 2.7 | 3.7 |
| Palau tropical moist forests | 13.0 | 0.0 |
| Palawan rain forests | 40.6 | 0.7 |
| Palouse grasslands | 1.7 | 0.2 |
| Pamir alpine desert and tundra | 16.7 | 1.8 |
| Panamanian dry forests | 16.5 | 0.0 |
| Pannonian mixed forests | 15.7 | 0.0 |
| Pantanal | 35.2 | 0.2 |
| Pantanos de Centla | 23.4 | 0.1 |
| Pantepui | 16.1 | 18.0 |
| Paraguana xeric scrub | 21.3 | 0.6 |
| Parana flooded savanna | 21.8 | 0.1 |
| Paropamisus xeric woodlands | 16.3 | 0.0 |
| Patagonian steppe | 8.3 | 0.3 |
| Patea Valley dry forests | 48.0 | 0.0 |
| Peninsular Malaysian montane rain forests | 135.0 | 0.0 |
| Peninsular Malaysian peat swamp forests | 128.0 | 0.0 |
| Peninsular Malaysian rain forests | 124.4 | 1.4 |
| Pernambuco coastal forests | 33.0 | 0.0 |
| Pernambuco interior forests | 26.4 | 0.0 |
| Persian Gulf desert and semi-desert | 10.4 | 0.8 |
| Peruvian Yungas | 35.3 | 4.6 |
| Peten-Veracruz moist forests | 28.7 | 1.3 |
| Pilbara shrublands | 5.7 | 0.3 |
| Pindus Mountains mixed forests | 14.6 | 0.0 |
| Piney Woods forests | 15.8 | 1.6 |
| Po Basin mixed forests | 12.4 | 0.0 |
| Pontic steppe | 16.9 | 0.0 |
| Puerto Rican moist forests | 22.9 | 0.5 |
| Puget lowland forests | 7.7 | 1.6 |
| Purus-Madeira moist forests | 24.4 | 12.9 |
| Purus varzea | 22.7 | 12.3 |
| Pyrenees conifer and mixed forests | 16.2 | 0.1 |
| Qaidam Basin semi-desert | 8.5 | 3.2 |
| Qilian Mountains conifer forests | 23.3 | 0.0 |
| Qilian Mountains subalpine meadows | 14.8 | 1.4 |
| Qin Ling Mountains deciduous forests | 26.4 | 0.0 |
| Qionglai-Minshan conifer forests | 38.0 | 0.2 |
| Queen Charlotte Islands | 5.4 | 5.4 |
| Queensland tropical rain forests | 18.1 | 1.1 |
| Rakiura Island temperate forests | 32.5 | 3.0 |
| Rann of Kutch seasonal salt marsh | 34.1 | 0.8 |
| Red River freshwater swamp forests | 31.8 | 0.0 |
| Red Sea coastal desert | 11.8 | 2.5 |
| Red Sea Nubo-Sindian tropical desert and semi-desert | 8.4 | 1.2 |
| Registan-North Pakistan sandy desert | 13.9 | 0.7 |
| Richmond temperate forests | 22.3 | 0.7 |
| Rio Negro campinarana | 11.8 | 20.7 |
| Rock and Ice | 1.1 | 0.8 |
| Rodope montane mixed forests | 20.5 | 0.0 |
| Rwenzori-Virunga montane moorlands | 43.0 | 11.5 |
| Sahara desert | 4.3 | 4.5 |
| Saharan flooded grasslands | 27.2 | 0.0 |
| Saharan halophytics | 8.3 | 2.3 |
| Sahelian Acacia savanna | 16.4 | 1.4 |
| Sakhalin Island taiga | 14.1 | 0.8 |
| San Lucan xeric scrub | 11.0 | 0.5 |
| Santa Marta montane forests | 43.8 | 1.2 |
| Santa Marta paramo | 47.5 | 2.0 |
| Sao Tome, Principe and Annobon moist lowland forests | 15.0 | 4.0 |
| Sarmatic mixed forests | 8.7 | 0.0 |
| Sayan Alpine meadows and tundra | 12.5 | 4.0 |
| Sayan Intermontane steppe | 15.2 | 2.8 |
| Sayan montane conifer forests | 11.9 | 3.2 |
| Scandinavian and Russian taiga | 4.8 | 0.3 |
| Scandinavian coastal conifer forests | 5.2 | 0.0 |
| Scandinavian Montane Birch forest and grasslands | 3.2 | 0.2 |
| Sechura desert | 16.0 | 1.5 |
| Selenge-Orkhon forest steppe | 17.2 | 0.4 |
| Seram rain forests | 20.5 | 0.6 |
| Serengeti volcanic grasslands | 40.3 | 0.0 |
| Serra do Mar coastal forests | 74.9 | 0.6 |
| Sichuan Basin evergreen broadleaf forests | 22.7 | 0.0 |
| Sierra de la Laguna dry forests | 16.0 | 0.7 |
| Sierra de la Laguna pine-oak forests | 9.0 | 0.0 |
| Sierra de los Tuxtlas | 41.0 | 0.5 |
| Sierra Juarez and San Pedro Martir pine-oak forests | 9.5 | 1.0 |
| Sierra Madre de Chiapas moist forests | 31.2 | 0.4 |
| Sierra Madre de Oaxaca pine-oak forests | 25.1 | 0.8 |
| Sierra Madre del Sur pine-oak forests | 17.8 | 1.3 |
| Sierra Madre Occidental pine-oak forests | 13.5 | 0.7 |
| Sierra Madre Oriental pine-oak forests | 14.5 | 1.2 |
| Sierra Nevada forests | 6.3 | 1.5 |
| Simpson desert | 3.3 | 1.2 |
| Sinaloan dry forests | 15.9 | 0.4 |
| Sinu Valley dry forests | 27.7 | 0.1 |
| Snake-Columbia shrub steppe | 2.7 | 0.3 |
| Society Islands tropical moist forests | 20.0 | 0.0 |
| Socotra Island xeric shrublands | 9.0 | 0.3 |
| Solimes-Japura moist forests | 18.5 | 15.5 |
| Solomon Islands rain forests | 21.7 | 0.8 |
| Somali Acacia-Commiphora bushlands and thickets | 16.1 | 0.1 |
| Somali montane xeric woodlands | 12.1 | 0.9 |
| Sonoran-Sinaloan transition subtropical dry forest | 13.4 | 0.4 |
| Sonoran desert | 6.8 | 0.8 |
| South American Pacific mangroves | 39.4 | 3.0 |
| South Appenine mixed montane forests | 15.6 | 0.5 |
| South Avalon-Burin oceanic barrens | 8.0 | 1.0 |
| South Central Rockies forests | 3.2 | 0.5 |
| South China-Vietnam subtropical evergreen forests | 26.6 | 0.1 |
| South Deccan Plateau dry deciduous forests | 32.3 | 0.0 |
| South Florida rocklands | 8.0 | 5.0 |
| South Iran Nubo-Sindian desert and semi-desert | 15.6 | 0.4 |
| South Island montane grasslands | 18.8 | 2.2 |
| South Island temperate forests | 21.9 | 0.6 |
| South Malawi montane forest-grassland mosaic | 25.5 | 0.8 |
| South Saharan steppe and woodlands | 5.3 | 6.3 |
| South Sakhalin-Kurile mixed forests | 14.8 | 0.7 |
| South Siberian forest steppe | 11.6 | 0.0 |
| South Taiwan monsoon rain forests | 26.5 | 0.0 |
| South Western Ghats moist deciduous forests | 56.3 | 0.6 |
| South Western Ghats montane rain forests | 60.7 | 0.5 |
| Southeast Australia temperate forests | 15.4 | 0.1 |
| Southeast Australia temperate savanna | 9.4 | 0.0 |
| Southeast Tibet shrublands and meadows | 23.9 | 0.0 |
| Southeastern conifer forests | 17.9 | 0.7 |
| Southeastern Iberian shrubs and woodlands | 20.0 | 1.0 |
| Southeastern Indochina dry evergreen forests | 47.8 | 0.3 |
| Southeastern mixed forests | 12.3 | 1.3 |
| Southeastern Papuan rain forests | 12.9 | 0.2 |
| Southern Acacia-Commiphora bushlands and thickets | 31.3 | 0.3 |
| Southern Africa bushveld | 24.7 | 0.1 |
| Southern Africa mangroves | 34.0 | 0.0 |
| Southern Anatolian montane conifer and deciduous forests | 20.0 | 0.1 |
| Southern Andean steppe | 6.1 | 0.3 |
| Southern Andean Yungas | 17.8 | 0.3 |
| Southern Annamites montane rain forests | 51.4 | 1.7 |
| Southern Atlantic mangroves | 49.6 | 0.3 |
| Southern Cone Mesopotamian savanna | 28.2 | 0.0 |
| Southern Congolian forest-savanna mosaic | 12.6 | 0.2 |
| Southern Great Lakes forests | 12.1 | 0.2 |
| Southern Hudson Bay taiga | 1.0 | 3.5 |
| Southern Korea evergreen forests | 21.7 | 0.8 |
| Southern Mesoamerican Pacific mangroves | 25.3 | 0.0 |
| Southern Miombo woodlands | 24.9 | 0.3 |
| Southern New Guinea freshwater swamp forests | 6.6 | 1.1 |
| Southern New Guinea lowland rain forests | 5.9 | 0.9 |
| Southern Pacific dry forests | 21.6 | 0.3 |
| Southern Rift montane forest-grassland mosaic | 28.4 | 0.6 |
| Southern Vietnam lowland dry forests | 42.9 | 0.1 |
| Southern Zanzibar-Inhambane coastal forest mosaic | 19.3 | 0.4 |
| Southwest Amazon moist forests | 27.6 | 11.2 |
| Southwest Australia savanna | 4.7 | 0.1 |
| Southwest Australia woodlands | 11.4 | 0.3 |
| Southwest Borneo freshwater swamp forests | 105.9 | 2.5 |
| Southwest Iberian Mediterranean sclerophyllous and mixed forests | 21.0 | 0.7 |
| Southwestern Arabian foothills savanna | 10.9 | 0.6 |
| Southwestern Arabian montane woodlands | 12.6 | 1.1 |
| Sri Lanka dry-zone dry evergreen forests | 26.9 | 0.8 |
| Sri Lanka lowland rain forests | 39.2 | 8.5 |
| Sri Lanka montane rain forests | 54.0 | 1.0 |
| Succulent Karoo | 16.6 | 1.9 |
| Suiphun-Khanka meadows and forest meadows | 24.0 | 0.4 |
| Sulaiman Range alpine meadows | 18.6 | 0.2 |
| Sulawesi lowland rain forests | 35.9 | 0.4 |
| Sulawesi montane rain forests | 38.5 | 0.1 |
| Sulu Archipelago rain forests | 22.0 | 1.3 |
| Sumatran freshwater swamp forests | 116.0 | 0.0 |
| Sumatran lowland rain forests | 111.2 | 0.1 |
| Sumatran montane rain forests | 116.9 | 0.2 |
| Sumatran peat swamp forests | 120.2 | 0.5 |
| Sumatran tropical pine forests | 113.3 | 0.0 |
| Sumba deciduous forests | 21.9 | 0.3 |
| Sunda Shelf mangroves | 113.1 | 0.5 |
| Sundaland heath forests | 103.8 | 0.5 |
| Sundarbans freshwater swamp forests | 25.4 | 0.1 |
| Sundarbans mangroves | 26.3 | 0.9 |
| Swan Coastal Plain Scrub and Woodlands | 12.2 | 0.2 |
| Taiheiyo evergreen forests | 21.3 | 0.1 |
| Taiheiyo montane deciduous forests | 18.2 | 0.0 |
| Taimyr-Central Siberian tundra | 0.7 | 1.8 |
| Taiwan subtropical evergreen forests | 27.5 | 0.1 |
| Taklimakan desert | 8.9 | 2.2 |
| Talamancan montane forests | 45.8 | 1.2 |
| Tamaulipan matorral | 14.7 | 0.2 |
| Tamaulipan mezquital | 9.7 | 0.4 |
| Tapajos-Xingu moist forests | 22.2 | 22.1 |
| Tarim Basin deciduous forests and steppe | 9.7 | 2.1 |
| Tasmanian Central Highland forests | 13.7 | 0.2 |
| Tasmanian temperate forests | 23.6 | 1.9 |
| Tasmanian temperate rain forests | 19.9 | 3.3 |
| Tehuacan Valley matorral | 17.9 | 0.8 |
| Tenasserim-South Thailand semi-evergreen rain forests | 80.6 | 2.9 |
| Terai-Duar savanna and grasslands | 59.2 | 0.0 |
| Texas blackland prairies | 16.1 | 0.0 |
| Thar desert | 25.2 | 1.0 |
| Tian Shan foothill arid steppe | 19.7 | 0.0 |
| Tian Shan montane conifer forests | 18.3 | 0.0 |
| Tian Shan montane steppe and meadows | 17.5 | 0.0 |
| Tibesti-Jebel Uweinat montane xeric woodlands | 6.7 | 2.4 |
| Tibetan Plateau alpine shrublands and meadows | 14.5 | 0.5 |
| Tigris-Euphrates alluvial salt marsh | 19.3 | 0.7 |
| Timor and Wetar deciduous forests | 24.3 | 0.3 |
| Tirari-Sturt stony desert | 4.4 | 0.2 |
| Tocantins/Pindare moist forests | 39.4 | 0.7 |
| Tonle Sap-Mekong peat swamp forests | 36.6 | 0.0 |
| Tonle Sap freshwater swamp forests | 36.8 | 0.0 |
| Torngat Mountain tundra | 0.3 | 4.7 |
| Trans-Baikal Bald Mountain tundra | 2.9 | 7.4 |
| Trans-Baikal conifer forests | 12.6 | 3.8 |
| Trans-Mexican Volcanic Belt pine-oak forests | 19.7 | 0.9 |
| Trans Fly savanna and grasslands | 8.7 | 0.3 |
| Trinidad and Tobago moist forests | 12.4 | 0.6 |
| Trobriand Islands rain forests | 10.0 | 1.0 |
| Tuamotu tropical moist forests | 18.0 | 2.0 |
| Tumbes-Piura dry forests | 25.1 | 1.2 |
| Tyrrhenian-Adriatic Sclerophyllous and mixed forests | 16.3 | 0.6 |
| Uatuma-Trombetas moist forests | 15.4 | 19.3 |
| Ucayali moist forests | 38.7 | 7.5 |
| Upper Gangetic Plains moist deciduous forests | 43.6 | 0.0 |
| Upper Midwest forest-savanna transition | 10.5 | 0.1 |
| Ural montane forests and tundra | 8.7 | 1.3 |
| Uruguayan savanna | 21.1 | 0.1 |
| Ussuri broadleaf and mixed forests | 15.4 | 4.0 |
| Valdivian temperate forests | 12.7 | 2.3 |
| Vanuatu rain forests | 15.5 | 1.2 |
| Venezuelan Andes montane forests | 39.7 | 0.5 |
| Veracruz dry forests | 32.4 | 0.6 |
| Veracruz moist forests | 24.8 | 0.5 |
| Veracruz montane forests | 31.9 | 0.0 |
| Victoria Basin forest-savanna mosaic | 29.8 | 0.3 |
| Victoria Plains tropical savanna | 3.2 | 0.7 |
| Vogelkop-Aru lowland rain forests | 10.5 | 0.7 |
| Vogelkop montane rain forests | 13.6 | 0.3 |
| Wasatch and Uinta montane forests | 3.9 | 0.2 |
| West Saharan montane xeric woodlands | 8.4 | 2.9 |
| West Siberian taiga | 5.7 | 2.4 |
| West Sudanian savanna | 17.2 | 0.0 |
| Western Australian Mulga shrublands | 1.6 | 0.0 |
| Western Congolian forest-savanna mosaic | 11.7 | 0.5 |
| Western Congolian swamp forests | 12.1 | 3.3 |
| Western Ecuador moist forests | 41.2 | 0.4 |
| Western European broadleaf forests | 8.0 | 0.0 |
| Western Great Lakes forests | 5.9 | 1.9 |
| Western Guinean lowland forests | 27.2 | 18.8 |
| Western Gulf coastal grasslands | 17.8 | 0.7 |
| Western Himalayan alpine shrub and Meadows | 20.5 | 0.1 |
| Western Himalayan broadleaf forests | 33.9 | 0.1 |
| Western Himalayan subalpine conifer forests | 31.1 | 0.2 |
| Western Java montane rain forests | 51.4 | 0.0 |
| Western Java rain forests | 47.0 | 0.1 |
| Western short grasslands | 8.4 | 0.4 |
| Western Siberian hemiboreal forests | 14.4 | 0.2 |
| Western Zambezian grasslands | 23.9 | 0.2 |
| Westland temperate forests | 31.3 | 6.8 |
| Willamette Valley forests | 4.6 | 0.4 |
| Windward Islands moist forests | 9.0 | 0.0 |
| Wrangel Island arctic desert | 0.6 | 2.0 |
| Wyoming Basin shrub steppe | 5.4 | 0.3 |
| Xingu-Tocantins-Araguaia moist forests | 32.1 | 9.1 |
| Yamal-Gydan tundra | 1.5 | 1.7 |
| Yapen rain forests | 14.3 | 0.7 |
| Yarlung Tsangpo arid steppe | 17.8 | 0.1 |
| Yellow Sea saline meadow | 27.1 | 0.0 |
| Yucatan dry forests | 16.0 | 0.6 |
| Yucatan moist forests | 19.7 | 1.8 |
| Yukon Interior dry forests | 1.8 | 1.5 |
| Yunnan Plateau subtropical evergreen forests | 26.3 | 0.0 |
| Zagros Mountains forest steppe | 21.7 | 0.1 |
| Zambezian and Mopane woodlands | 25.1 | 0.8 |
| Zambezian Baikiaea woodlands | 23.5 | 1.7 |
| Zambezian coastal flooded savanna | 24.7 | 0.1 |
| Zambezian Cryptosepalum dry forests | 23.0 | 0.2 |
| Zambezian flooded grasslands | 26.1 | 1.6 |
| Zambezian halophytics | 24.7 | 0.6 |
