# Supplementary material for: Hotspots of human impact on threatened terrestrial vertebrates
Source: PLoS Biol. 2019 Mar 12;17(3):e3000158. doi: 10.1371/journal.pbio.3000158 (PMC6413901; doi:10.1371/journal.pbio.3000158)
Supplement: S6 Table — (DOCX) [file pbio.3000158.s012.docx]

**Table S6.** Weights assigned to individual pressures in the Human Footprint, and threshold scheme used to convert pressures into binary scores (present or absent) for impact analyses.

| **Pressure** | **Score** | **Details** | **Threshold for conversion to binary** |
| --- | --- | --- | --- |
| Built environments | 0,10 | All built areas given score of 10 | pressure present or absent |
| Population density | 0-10 Continuous | Score = 3.333xlog(population density+1) | Pressure considered present for scores ≥ 1. |
| Night-time lights | 0-10 Continuous | Equal quintile bins | Pressure considered present for scores ≥ 1. |
| Croplands | 0,7 | All cropland given score of 7 | pressure present or absent |
| Pasture | 0,4 | All pasture given score of 4 | pressure present or absent |
| Roads | 0,8 Direct impacts 0-4 indrect impacts | 500m either side of road given a direct pressure score of 8. Starting 500m out from road, pressure score of 4 exponentially decaying out to 15km | Pressure considered present up to 3km either side of the road (equivalent human footprint score = 1) |
| Railways | 0,8 | 500m either side of railway given a direct pressure score of 8 | pressure present or absent |
| Navigable waterways | 0-4 | pressure score of 4 exponentially decaying out to 15km | Pressure considered present up to 1.5 km either side of the waterway (equivalent human footprint score = 3.5) |
