# Supplementary material for: Correction: Hotspots of human impact on threatened terrestrial vertebrates
Source: PLoS Biol. 2019 Dec 16;17(12):e3000598. doi: 10.1371/journal.pbio.3000598 (PMC6913904; doi:10.1371/journal.pbio.3000598)
Supplement: S1 File — (PDF) [file pbio.3000598.s001.pdf]

PLOS BIOLOGY

RESEARCH ARTICLE

Hotspots of human impact on threatened terrestrial vertebrates

James R. Allan[1,2]*, **James E. M. Watson**[1,2,3], **Moreno Di Marco**[1,4], **Christopher J. O'Bryan**[1,2], **Hugh P. Possingham**[2,5], **Scott C. Atkinson**[2,6], **Oscar Venter**[7]

1 School of Earth and Environmental Sciences, The University of Queensland, Brisbane, Australia, **2** Centre for Biodiversity and Conservation Science, The University of Queensland, Brisbane, Australia, **3** Wildlife Conservation Society, Global Conservation Program, New York, New York, United States of America, **4** CSIRO Land & Water, EcoSciences Precinct, Brisbane, Australia, **5** The Nature Conservancy, Arlington, Virginia, United States of America, **6** United Nations Development Programme (UNDP), New York, New York, United States of America, **7** Natural Resource and Environmental Studies Institute, University of Northern British Columbia, Prince George, Canada

* j.allan2@uq.edu.au

Check for updates

OPEN ACCESS

Citation: Allan JR, Watson JEM, Di Marco M, O'Bryan CJ, Possingham HP, Atkinson SC, et al. (2019) Hotspots of human impact on threatened terrestrial vertebrates. PLoS Biol 17(3): e3000158. https://doi.org/10.1371/journal.pbio.3000158

Academic Editor: Pedro Jordano, Estacion Biologica de Doñana CSIC, SPAIN

Received: August 2, 2018

Accepted: February 5, 2019

Published: March 12, 2019

Copyright: © 2019 Allan et al. This is an open access article distributed under the terms of the Creative Commons Attribution License, which permits unrestricted use, distribution, and reproduction in any medium, provided the original author and source are credited.

Data Availability Statement: The underlying data are all publicly available. Species distribution data available from (https://www.iucnredlist.org/) and (http://datazone.birdlife.org/home). Human pressure data are available from (http://dx.doi.org/10.5061/dryad.052q5). The processed data underlying our analyses and figures are freely available here: (doi:10.1594/PANGAEA.897391).

Funding: The authors received no specific funding for this work.

Competing interests: The authors have declared that no competing interests exist.

Abstract

Conserving threatened species requires identifying where across their range they are being impacted by threats, yet this remains unresolved across most of Earth. Here, we present a global analysis of cumulative human impacts on threatened species by using a spatial framework that jointly considers the co-occurrence of eight threatening processes and the distribution of 5,457 terrestrial vertebrates. We show that impacts to species are widespread, occurring across 84% of Earth's surface, and identify hotspots of impacted species richness and coolspots of unimpacted species richness. Almost one-quarter of assessed species are impacted across >90% of their distribution, and approximately 7% are impacted across their entire range. These results foreshadow localised extirpations and potential extinctions without conservation action. The spatial framework developed here offers a tool for defining strategies to directly mitigate the threats driving species' declines, providing essential information for future national and global conservation agendas.

Author summary

The biggest drivers of global biodiversity loss are hunting, harvesting, and the conversion of natural habitats for agriculture, urbanisation, and other industrial activity. However, our understanding of where these 'threats' actually impact sensitive species is extremely limited across Earth. Here, we map the distribution of threats within the known ranges of 5,457 terrestrial birds, mammals, and amphibians globally. We map only those threats within a species range that are known to specifically endanger that species. We show that threats are extensive across the majority of species' ranges, severely limiting the area within which species can survive. Concerningly, 1,237 species (almost one-quarter of those assessed) are impacted by threats across >90% of their distribution, and 395 species are impacted by threats across their entire range. These species will almost certainly face extinction without conservation intervention to remove threats. We identify global

Abbreviations: IUCN, International Union for Conservation of Nature; SDG, United Nations Sustainable Development Goal.

hotspots of impacted species richness and also ‘coolspots’ that act as refuges from threats, providing essential information for conservation planning and action.

Introduction

Human activities and land usage are exerting unprecedented pressure on natural environments [1,2], threatening to drive tens of thousands of species to extinction [3]. The main drivers of species declines include the conversion of natural habitats for land usage such as crops, pasture, and infrastructure, as well as the overexploitation of species through activities such as hunting [3,4]. The distribution of these activities varies across Earth’s terrestrial surface [1], as do the distributions of the species they threaten [5]. Understanding and quantifying spatial patterns of where human pressures overlap with sensitive species (i.e., mapping human impacts to threatened species) will improve our ability to prioritise actions to manage and mitigate human impacts on biodiversity [6,7]. Importantly, it will allow for the identification of areas across species distributions that are free from those threats that the species is sensitive to, and this information can be used to map global ‘coolspots’ of what we call ‘threat refugia’. Both forms of information are essential for conservation planning and can guide action towards securing these impact-free refugia, which are paramount for the survival of many threatened species [8–11].

Mapping impacts to biodiversity requires linking spatial data on the distributions of threats with the distributions of species known to be sensitive to those threats [12]. To date, no efforts undertaken at either regional [13,14] or global extents [1,15–18] have accounted for the distribution and sensitivity of species and their threats and therefore do not directly map likely human impacts [19]. Past efforts that simply map threats [1] fail to account for the distribution of species that respond to those threats, and even overlapping threats with species ranges [20] does not account for the specific sensitivities of each species to co-occurring threats. Some efforts to map threats to the marine realm estimated their impacts at the coarse ecosystem scale but did not account for individual species sensitivities [12,21]. The few studies that do account for species have either been conducted at fine spatial resolutions [22] or consider a limited number of taxonomic groups [23,24], and many suffer from the assumption that species are exposed to threats across their entire range, not just where the threat occurs, overestimating impacts [20,25,26]. Clearly, our understanding of where individual species are being impacted by threats or where their threat-free refugia are remains limited at the global scale [27] and is a major gap in our ability to prioritise conservation actions [27,28].

Here, we present the first global assessment of the spatial distribution of human impacts on globally threatened and near-threatened terrestrial birds, mammals, and amphibians. We developed a novel, to our knowledge, method for quantifying and mapping human impacts that jointly considers the distributions of 5,457 threatened and near-threatened species (1,277 mammals, 2,120 birds, and 2,060 amphibians), the distribution of species-specific threats, and the extent to which the distribution of each species is impacted by relevant threats (Fig 1).

Spatial data on threats were obtained from the recently updated Human Footprint [1], which is unique for considering eight human pressures globally at a 1 km^2 resolution, including built environments, crop lands, pasture lands, human population density, night lights, railways, major roadways, and navigable waterways. This makes the Human Footprint the most complete and highest resolution globally consistent dataset of anthropogenic threats [29]. Each individual pressure was linked to a species if they directly or indirectly correspond to threats identified by the International Union for Conservation of Nature (IUCN) Red List [30]

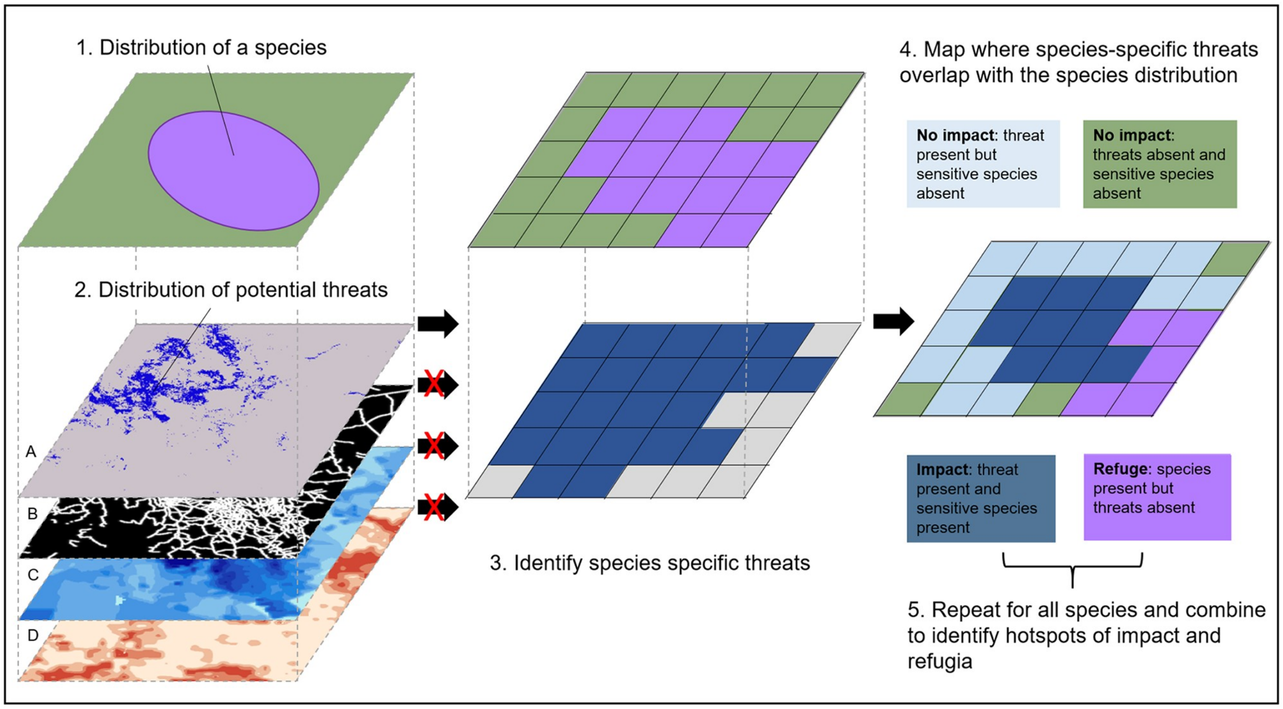

Fig 1. Methodological framework for mapping cumulative human impacts on threatened vertebrate species.

https://doi.org/10.1371/journal.pbio.3000158.g001

as driving the endangerment of that species. The Human Footprint data correspond with seven major classes and 15 subclasses of IUCN threats (Table 1; S1 Table). Although these do not include all threats to species, they do include all of the most prevalent drivers of global biodiversity decline [4]. We calculated the proportion of each species range that is currently impacted by a threat and then mapped cumulative human impacts in a 30 km × 30 km grid globally (see Materials and methods). We also examined patterns of human impacts across individual species distributions, taxonomic groups, and threat status categories. Finally, we used the inverse of our cumulative impact maps to identify threat refugia, the places where high numbers of threatened (and near-threatened) species persist unimpacted by human activity.

Results

Human impacts on threatened vertebrate species

We found that on average, 38% of a species' distribution range is impacted by one or more relevant threats (Table 2, S1 Data), including an average 21% of the distribution impacted by multiple co-occurring threats. Mammals are the most impacted of all taxa, with on average 52% of a species' distribution impacted by relevant threats. Concerningly, almost one-quarter of all species (23%, $n = 1{,}237$) are impacted by threats across >90% of their distribution, with 395 (7%) impacted by at least one relevant threat across their entire distribution. Conversely, we found that one-third of all species (34%, $n = 1{,}863$) are not exposed to the threats we mapped across any portion of their distribution; however, this result should be interpreted within the context of threats we consider. We also found that the proportion of a species distribution impacted by threats correlates with its threat status (IUCN Red List categories; Fig 2)

Table 1. Major classes and subclasses of threats to biodiversity, as classified in the IUCN Red List of Threatened Species, and the corresponding spatially explicit pressure variable from the updated Human Footprint dataset.

Major threat class (IUCN)	Subclass threats (IUCN)	Pressure (Human Footprint)	Species Impacted
1. Residential and commercial development	1.1 Housing and urban areas	Electric infrastructure (nightlights)	1,748
		Built environments	
	1.2 Commercial and industrial areas	Electric infrastructure (nightlights)	349
		Built environments	
2. Agriculture and aquaculture	2.1 Annual and perennial nontimber crops	Crop lands	4,017
	2.3 Livestock farming and ranching	Pasture lands	1,850
4. Transportation and service corridors	4.1 Roads and railroads	Railways	563
		Roads	
	4.2 Utility and service lines	Roads	88
5. Biological resource use	5.1 Hunting and collecting terrestrial animals	Navigable waterways	1,594
		Population density	
		Roads	
	5.2 Gathering terrestrial plants	Navigable waterways	149
		Population density	
		Roads	
6. Human intrusions and disturbance	6.1 Recreational activities*	Electric infrastructure (nightlights)	
		Population density	373
	6.3 Work and other activities	Electric infrastructure (nightlights)	196
		Population density	
8. Invasive and other problematic species, genes, and diseases	8.1 Invasive non-native/alien species/diseases	Population density	1,319
		Roads	
9. Pollution	9.1 Domestic and urban waste water	Population density	205
		Built environments	
	9.3 Agriculture and forestry effluents	Crop lands	805
	9.4 Garbage and solid waste	Built environments	27
	9.6 Excess energy	Electric infrastructure (nightlights)	24
		Built environments	

*We excluded navigable waterways because these pressures are generally limited to aquatic-dwelling species by the IUCN, and our analysis focuses on terrestrial species. Additionally, we excluded roads because the pressures described by the IUCN for this category are generally limited to dirt roads, which are not represented in the Human Footprint. **Abbreviation**: IUCN, International Union for Conservation of Nature.

https://doi.org/10.1371/journal.pbio.3000158.t001

(analysis of variance $P < 0.001$, F = 7.5). Species classified as critically endangered on the IUCN Red List had almost half their distribution impacted by threats on average (46%, $n = 851$), whilst near-threatened species had one-third of their distribution impacted by threats on average (31%, $n = 1{,}439$).

Table 2. The number and percentage of species and the proportion of their distribution impacted by threats.

		Proportion of range impacted by threats					
	Total number of species	0%	1%–50%	50%–90%	90%–99%	100%	Mean (%)
**Amphibians**	2,060	**1,082 (52.5%)**	293 (14.2%)	301 (14.6%)	213 (10.3%)	171 (8.3%)	31.5
**Birds**	2,120	387 (18.3%)	**911 (43%)**	442 (20.8%)	292 (13.8%)	88 (4.2%)	37.2
**Mammals**	1,277	337 (26.4%)	259 (20.3%)	216 (16.9%)	**354 (27.7)**	111 (8.7%)	51.5
**Total**	5,457	**1,806 (33.1%)**	1,463 (26.8)	959 (17.6%)	859 (15.7%)	370 (6.8%)	38.4

The most common category for each taxon is shown in bold.

https://doi.org/10.1371/journal.pbio.3000158.t002

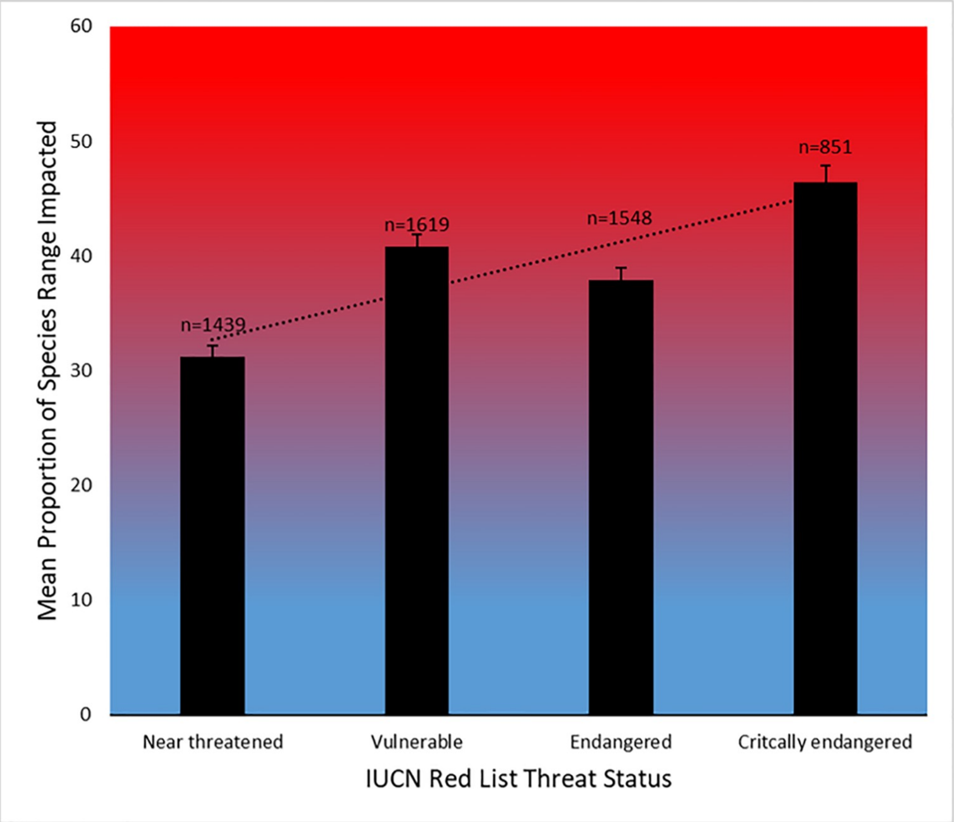

Fig 2. Mean proportion of species distributions impacted by threats across extinction risk categories of threatened and near-threatened terrestrial vertebrates. Bars represent means with standard errors. The data underlying this figure are freely available [31] (doi:10.1594/PANGAEA.897391). Species extinction risk assessed by the IUCN (2015). IUCN, International Union for Conservation of Nature.

https://doi.org/10.1371/journal.pbio.3000158.g002

Global hotspots of human impact

Human impacts on threatened vertebrates are widespread, extending across 84% of Earth's terrestrial surface (S2 Table; S1 and S2 Figs). There is strong spatial variation in the intensity of human impacts, with alarming peaks in Southeast Asia (Fig 3). Hotspots of human impact differ spatially between taxa (S3 Fig) and, as expected, are largely driven by patterns of threatened species richness (S4 Fig) and human pressure, although they are not congruent.

The top five countries most impacted by anthropogenic threats to species are all found in Southeast Asia (S3 Table), which we confirm is overwhelmingly the dominant global hotspot of impacts to species [32]. Malaysia has the highest average human impact score (125 species impacted per grid cell), followed by Brunei and Singapore (124 and 112 species, respectively). These scores are substantially higher than the global average of 15.6 species impacted per grid cell. Concerningly, there are 13 grid cells (11,700 km^2) in Southeast Asia where >150 species are impacted by threats.

When aggregated across biomes and ecoregions, which represent distinct biogeographic spatial units at the global scale [33] (S4 and S5 Tables), the highest human impacts are in mangroves, where on average 35 species are impacted per grid cell. Human impacts are also high throughout the tropical forests that harbour Earth's richest biota and are critically important for biodiversity conservation [34]. The tropical and subtropical moist broadleaf forests in

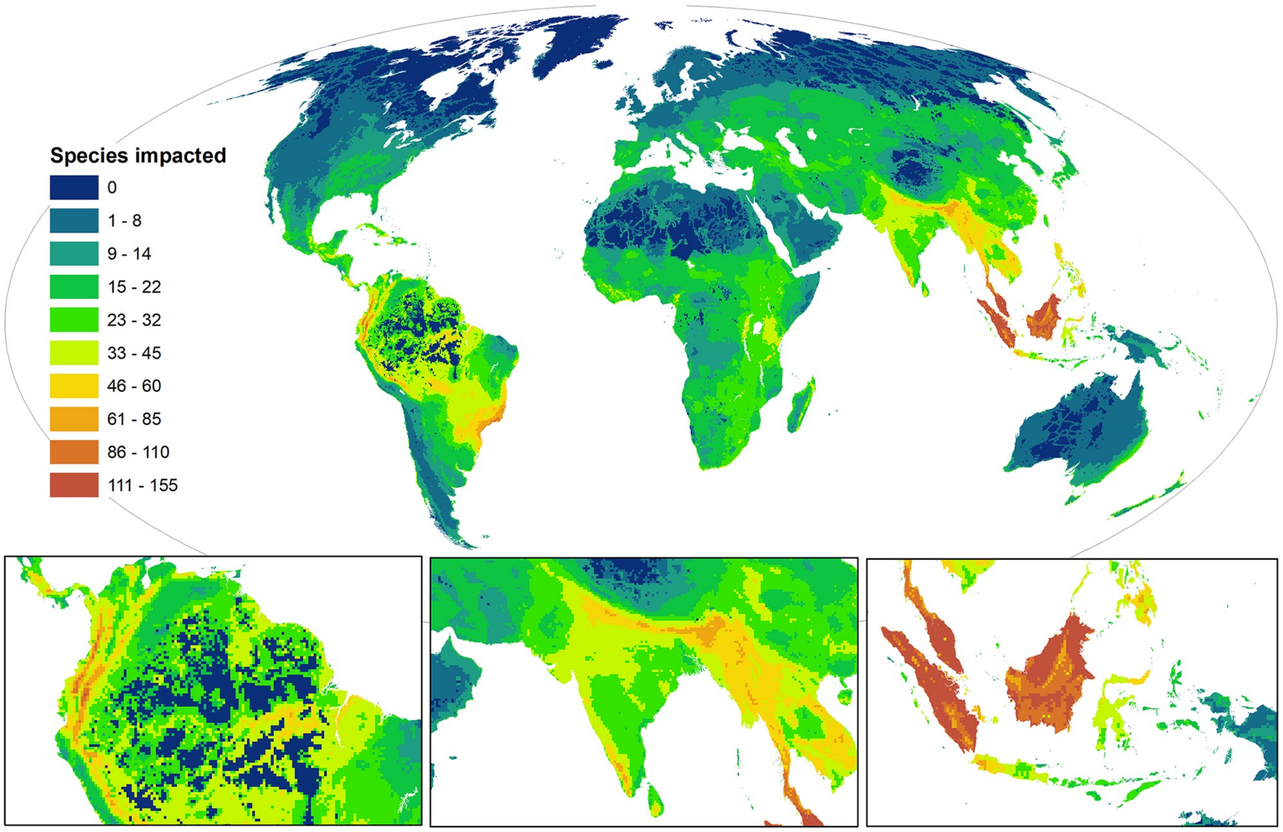

Fig 3. Cumulative human impacts on threatened and near-threatened terrestrial vertebrates (*n* = 5,457). Legend indicates the number of species in a grid cell impacted by at least one threat. Maps use a 30 km × 30 km grid and a Mollweide equal area projection. The data underlying this figure are freely available [31] (doi:10.1594/PANGAEA.897391).

https://doi.org/10.1371/journal.pbio.3000158.g003

Southeast Brazil, Malaysia, and Indonesia are the second most impacted biome, followed by the tropical and subtropical dry broadleaf forests in India, Myanmar, and Thailand (35 and 34 species impacted per 900 km^2 grid cell).

Global coolspots of threat refugia

We mapped threat refugia for threatened vertebrates by combining the unimpacted parts of each species' distribution. Almost the entire Earth's surface (97%) hosts at least one unimpacted threatened species, acting as a potential refugium for that species (Fig 4); however, impacted and unimpacted species co-occur across 80% of Earth's surface, identifying places where species with divergent sensitivities to threatening processes are present. There is strong spatial variation in the intensity of threat refugia for threatened species and between coolspots for different taxa (S5 Fig). Threat refugia often follow similar patterns to hotspots of impact, with Southeast Asia again the dominant global coolspot. Although counterintuitive, our results are largely driven by species richness and individual species' different sensitivities to threats. Therefore, in species-rich areas, it is logical that many species will be impacted, whilst many others remain unimpacted. The highest average threat refugia score is in Brunei (49 species unimpacted per grid cell), but the highest score for an individual grid cell occurs in Malaysia, where 144 species are unimpacted. Encouragingly, there are 12 grid cells (10,800 km^2) in

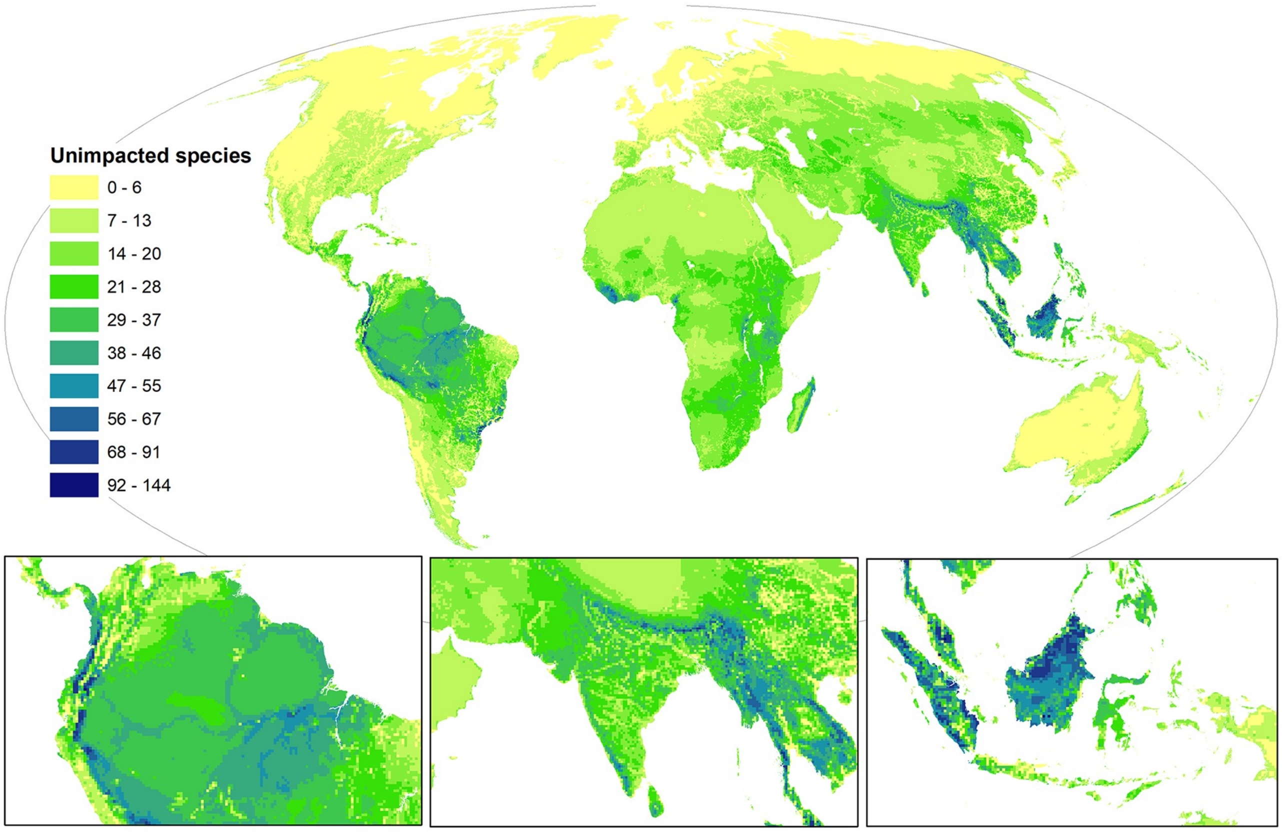

Fig 4. Coolspots of refugia for threatened and near-threatened terrestrial vertebrates (*n* = 5,457). Legend indicates the number of species that are not impacted by any threats in a grid cell. Maps use a 30 km × 30 km grid and a Mollweide equal area projection. The data underlying this figure are freely available [31] (doi:10.1594/PANGAEA.897391).

https://doi.org/10.1371/journal.pbio.3000158.g004

Southeast Asia with >100 unimpacted species, although this is primarily due to the large number of threatened species in the region.

Other coolspots of threat refugia include Liberia in West Africa, the Amazon rainforest and Andes mountains in South America, and the Eastern Himalayan biodiversity hotspot in Nepal, Bhutan, and Myanmar. When aggregated across biomes and ecoregions (S4 and S5 Tables), the tropical and subtropical moist broadleaf forests and tropical and subtropical dry broadleaf forests act as the greatest threat refugia, supporting on average 29 and 22 unimpacted species per grid cell, respectively. These are also two of the most impacted biomes, demonstrating that despite this, there are still considerable conservation opportunities here. The tundra and boreal forest are the only biomes where more species are unimpacted than impacted on average.

Proportion of species impacted

Some areas of the planet contain low numbers of threatened species (for example, the high latitudes or arid and desert regions). Therefore, it is instructive to examine the corresponding proportions of impacted versus unimpacted species. On average, there are more impacted than unimpacted species in a grid cell globally (15.6 versus 13.8; ratio 1.13) (Fig 5; S6 Fig). The proportion varies for taxonomic groups, with amphibians having the highest ratio of impacted

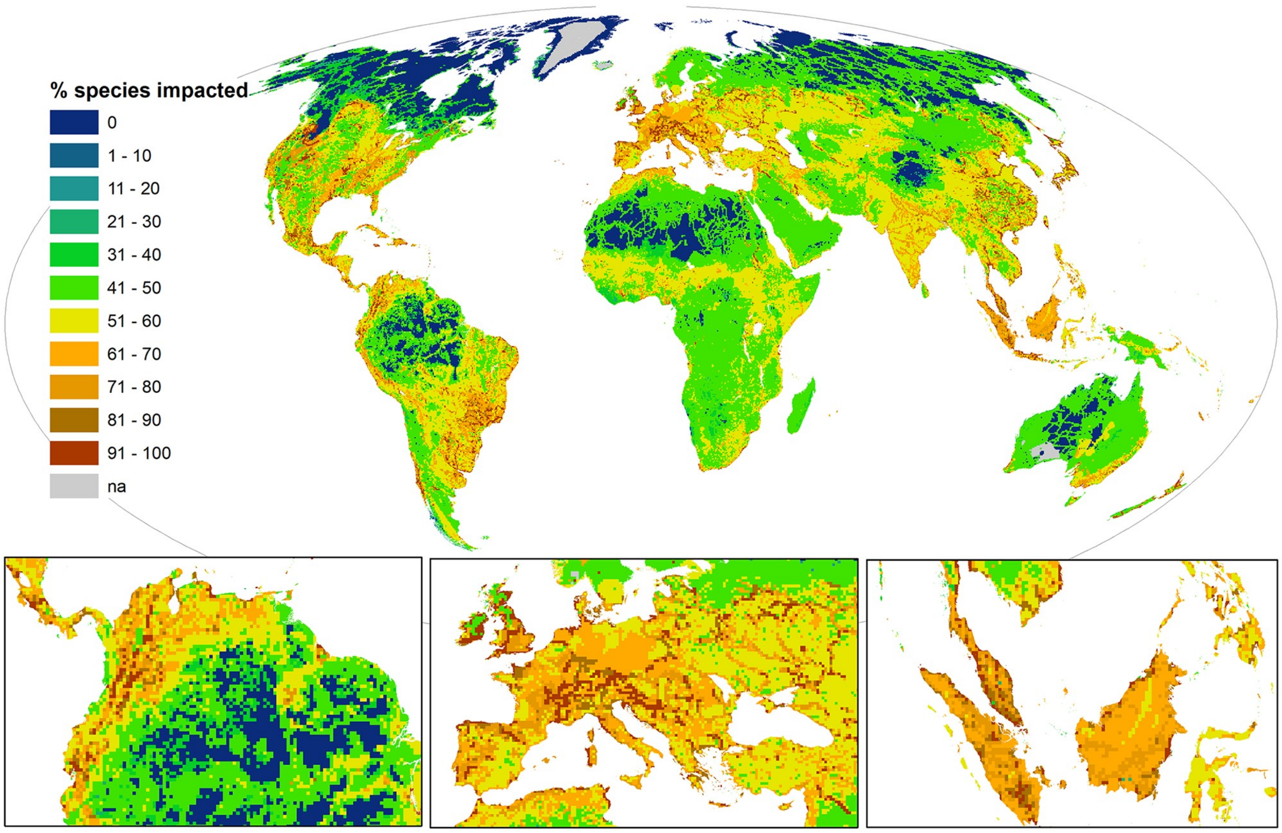

Fig 5. The percentage of species in a grid cell impacted by a threat (and inversely the number of unimpacted species for whom it is a refuge) for all taxa (*n* = 5,457). Maps use a 30 km × 30 km grid and a Mollweide equal area projection. The data underlying this figure are freely available [31] (doi:10.1594/PANGAEA.897391).

https://doi.org/10.1371/journal.pbio.3000158.g005

versus unimpacted species (2.3 versus 1.6; ratio 1.5) compared to birds and mammals (birds 10.5 versus 9.3, ratio 1.2 and mammals 5.4 versus 5.1, ratio 1.1).

In our 30 km^2 grid cells, the proportion of species impacted extends across the full range from 0%–100%. We found that >90% of species were impacted in 3,826 grid cells globally, amounting to a staggering 3.4 million km^2 (2.4% of Earth's terrestrial area), which is an area greater in size than India. Encouragingly, species are present, but none are impacted in 24,233 grid cells (21.8 million km^2; 15.1% of Earth's terrestrial area). The majority of this is wilderness where no human pressures occur. However, we found 919 grid cells (827,100 km^2; 0.5% of terrestrial area) where a species and a human pressure co-occur, but there is no impact (i.e., none of the species present are sensitive to the human activity or land use occurring in that area).

The distribution of areas with high proportions of impacted species differs substantially from hotspots of human impact. Europe and North and Central America now emerge as global hotspots, particularly for mammals and amphibians. The proportion of birds impacted presents a more spatially homogenous pattern, with hotspots in Southeast Asia and the Southeast South America. When aggregated across biomes, mangroves have the highest mean proportion of impacted species (61.3%), followed by temperate broadleaf and mixed forests (60.7%) (S4 Table). The tundra and boreal/taiga forests have the lowest mean proportions of impacted species (14.6% and 29%, respectively).

Discussion

Implications for biodiversity conservation

Our results represent the current best estimate of the spatial distribution of human impacts on terrestrial vertebrates. Continued extirpations, the precursors of extinction, will continue to occur in the impacted portions of species ranges, which our results demonstrate are substantial. Consequently, completely impacted species or those persisting in threat refugia that are too small to support viable populations in the long term [35] likely face imminent extinction. These findings complement recent work showing that hundreds of mammals have lost considerable portions of their historic distributions [36] and that habitat fragmentation has greatly reduced the proportion of highly suitable habitat within species distributions, reducing their movements [37] and increasing their extinction risk [38].

Although our results are concerning, there is room for hope. The threats we map can be mitigated by in situ conservation actions, but diverse approaches are required. To ensure the survival of highly impacted species with little or no threat refugia, active threat management, restoration, and rewilding efforts [39] are needed to open up enough viable habitat for species to persist. Conservation action in the hotspots of human impact we identify will have high benefits since they are areas with exceptionally high threatened species richness and species-specific threats [40]. Our results therefore extend previous efforts to identify biodiversity hotspots [40], which were developed following somewhat similar logic and have helped guide conservation action and millions of dollars of funding. The hotspots of human impact we identify are priorities for actions that mitigate the specific threats [41].

Rather than being purely reactive and focusing solely on securing a future for imperilled species in the short term, conservation efforts would also benefit from proactively securing coolspots of species refugia and avoiding any initial human impacts in these places [42]. This will help ensure many species' long-term persistence, especially in a time of rapid climate change, where areas free of threatening processes will be critical for species adaptation [43,44]. Securing refugia will be particularly effective if protection is targeted at the most species-rich places that currently remain threat free but may soon be jeopardised [45,46]. Additionally, conservation action is also likely to have a high chance of success in threat refugia and be more cost effective [47,48]. Proactive and reactive approaches to conservation have historically been pitted against each other [49], with reactive approaches deemed more urgent and taking precedence [49–51]. However, our discovery of the spatial overlap existing between hotspots of impacted species richness and coolspots of unimpacted species richness provides opportunities for multifaceted conservation action that is reactive for some species while simultaneously being proactive for others.

The utility of our work extends beyond conservation and can inform sustainable development planning. Conservation action within some of the hotspots of impact we identified (especially in Southeast Asia) are likely to deliver synergistic benefits to other environmental goals, such as carbon conservation and global reduction of deforestation rates [52]. Additionally, according to our definition, species threat refugia do not necessarily have to be off limits to human development, just free of the actions and land usage that directly threaten species found in that area. This provides a unique framework for quantifying the tradeoffs associated with the development of alternate human activities and land usage and for identifying locations and strategies to minimise their impacts on biodiversity. This has implications for nations striving to meet ambitious development targets such as the United Nations Sustainable Development Goals (SDGs), especially where achieving development goals involves tradeoffs with biodiversity goals [53,54]. The framework presented here could be adapted to inform conservation and development planning from local to regional scales and could be particularly useful in Southeast

Asia, Latin America, and sub-Saharan Africa, regions that are undergoing rapid economic development but are also hotspots of human impact and coolspots of threat refugia [55,56].

It is important to note that our data are not comprehensive of all threats to all species. For example, our analysis does not take into account infectious diseases, a driver of global declines in amphibians [57], or climate change, a threat already impacting many species across all taxa [43]. The results are therefore conservative, and many species will be more impacted than our maps indicate. Notably, one of the fundamental ways to manage global-scale threats such as climate change is to stop more easily abatable threats such as those considered in this analysis [58] to avoid antagonistic or synergistic interactions between multiple threats [59,60]. Other caveats worthy of discussion are that we assume the intensity of threats (for example, agricultural land use or roads) are equal across their distribution and that species are equally sensitive to each threat known to affect them. This assumption could mean we are overestimating impacts in cases in which species are sensitive to several threats where only the secondary threat is present. The IUCN has collected data on the severity of threats to species, but a comprehensive database is still lacking because this information is often unknown. The further development of these data would allow important nuances to be included in future extensions of this work.

A species and threat overlap does not necessarily mean that the threat is acting in that location. However, our analysis extends beyond a species threat overlay by incorporating three co-occurring and connected forms of data: a species distribution, a threat distribution, and that species' vulnerability to that threat. To the best of our knowledge, this is the first time species-specific sensitivity to threats has been incorporated into an impact mapping exercise at this scale. By mapping species-specific threats, it is much more likely that a threat is acting in a given location and impacting a species. This approach does rely on the current knowledge of threats to species and cannot account for the possibility that undocumented threats could be impacting a species. We sourced information on threats to species from the IUCN, who are the main authority on assessing species extinction risk, and limited our analyses to threatened terrestrial vertebrates, which include the most studied taxa globally [61]. Yet, it is important to note that there is still variation between species assessments because of taxonomic and geographical biases that could influence our findings [62]. For example, our understanding of threats to mammals is greater than for amphibians, which could partly explain why our results show mammals as the most impacted taxon, whilst amphibians are generally regarded as the more threatened taxon.

This analysis provides a framework for mapping human impacts that represents a conceptual advance over cumulative pressure mapping or threatened-species-richness mapping that can be applied to any scale, taxa, or realm. Furthermore, the framework and baseline can be continually updated and enhanced as additional data on species distributions, their sensitivity to threats, and the spatial distribution of threats become available and our understanding of threat interactions improves. Improvements in our understanding of species sensitivity to threats will also allow this analysis to be extended to other forms of life such as plant and invertebrate species. We have shown that human impacts on species are almost ubiquitous across Earth and that hundreds of species have no refuge from these impacts, including many of the most charismatic large mammals. The survival of these species, and many more, hinges on humanity's ability and willingness to compromise and share space.

Materials and methods

Spatial data on threatened species ranges

We focused our analysis on terrestrial vertebrate groups (amphibians, birds, mammals) with distribution maps and assessment of identified threat available for all species. Spatial data on

mammal and amphibian distributions were obtained from the IUCN Red List of Threatened Species [3] and bird distributions from Birdlife International and NatureServe [63]. We focused on species which are listed as near threatened, vulnerable, endangered, or critically endangered since their major threats have been identified and comprehensively assessed for the IUCN Red List of Threatened Species [3,4,64,65]. Following established practice, we only considered native and reintroduced parts of each species distribution range in our analysis, which are listed as extant, possibly extant, or possibly extinct within their range [66]. We excluded introduced, vagrant, and extinct species as well as species whose origin or presence is uncertain. Although reintroduced species ranges may be theoretically subject to fewer threats, they may still be under threats not realised during the reintroduction process [67]. As such, incorporating all portions of a species range, including reintroduced areas, can provide a robust picture of the threats for a given species. Finally, we only included species whose distribution overlapped (even just partially) with the extent of the Human Footprint threat dataset, which does not include Antarctica. A total of 2,060 amphibian species, 2,120 bird species, and 1,277 mammal species qualified for our analysis based on these criteria.

Spatial data on threats to species

Spatially explicit data on the distribution of threats to species were obtained from the recently updated Human Footprint maps [1,68]. These are globally standardised maps of cumulative human pressures on the natural environment at 1 km^2 resolution globally for eight of the most harmful pressures humans exert on nature, including 1) built environments, 2) population density, 3) electric infrastructure, 4) crop lands, 5) pasture lands, 6) roads, 7) railways, and 8) navigable waterways. This makes the Human Footprint the most up-to-date and comprehensive global cumulative pressure/threat map available [29]. The Human Footprint is also the first global-scale threat dataset to have been validated for accuracy. This was done by visually confirming if human pressures were present or absent across thousands of randomly selected 1 km × 1 km plots globally [68]. The data were found to exhibit an excellent degree of accuracy (88.5% agreement between visual plots and Human Footprint data), especially at identifying threat-free areas (98.9% agreement between visual plots and wilderness) [69].

In the Human Footprint, each pressure layer is scaled between 1 and 10 based on its estimated impact on the environment. These scores are then cumulated in each pixel to give a total score out of 50. We converted these scores to binary (present or absent in any 1 km^2 pixel) for our analyses since there are no data on the relative severity of individual threats to species. To convert pressure layers from continuous scales to binary (present/absent), we set cutoffs at which the pressure was considered absent. For example, roads have a direct pressure score of 8 up to 500 meters either side; beyond this, the pressure score decays exponentially from a score of 4 out to 0 at 15 km. When converting this to a binary score, we set a threshold that considered the pressure present up to 3 km either side of the road, and absent beyond this (see S6 Table for comprehensive details on how each layer was handled).

Mapping species-specific threats

We identified cases where the eight pressures in the updated Human Footprint dataset directly or indirectly correspond with threats to biodiversity as listed in the IUCN Red List [30] (Table 1, S1 Table). This allowed us to globally map seven major classes and 15 subclasses of threats. Although this is not comprehensive of all the threats to species, it importantly includes the biggest drivers of biodiversity declines globally [4]. For example, multiple forms of agriculture, urban development, and transportation corridors are directly accounted for by our pressure data, whilst biological resource use and overexploitation through hunting, pollution,

human disturbance, and invasive species are indirectly accounted for by human population density, roads and navigable river networks that act as proxies [56,58,70–72].

Analysing the extent of human impacts on individual species

For a pressure to impact a species, it must spatially overlap with that species' distribution and have been identified in the IUCN Red List as a threat to that species [19]. Therefore, we calculated the extent of overlap between each species distribution and each pressure layer that that species is sensitive to at a 1 km^2 resolution globally. We accounted for the overlap between threats, identifying where multiple threats are present. All spatial data were analysed in a Mollweide equal area projection in ESRI ArcGIS and PostGIS, and statistics were calculated in R statistical software. We used a one-way analysis of variance to test for correlation between a species extinction risk category and the proportion of that species range impacted by threats.

Mapping hotspots of cumulative human impacts

We estimated cumulative human impacts on threatened species using a global 30 km × 30 km planning unit grid, since this has been identified as the ideal resolution for reducing the effects of commission errors (where species are thought to be present but are not) when working with species range maps [73]. An impact was scored in a grid cell if a species and at least one threat it is sensitive to were both present. This means that the presence of a threat and a species in the same grid cell is not considered an impact unless the species is known to be sensitive to that threat. We then calculated the sum of all impacted species in a grid cell to give a total estimate of cumulative human impact.

As a sensitivity analysis, we calculated the area of each species distribution within each planning unit and the area of each pressure in each planning unit, converting both to proportions of planning unit area. To estimate how impacted each species is within each planning unit, we multiplied the proportion of the species distribution by the proportion of each pressure that threatens the species and then summed the scores. By using the proportion of planning unit area, we scale for the likelihood of a species and a pressure overlapping within a grid cell. Finally, we calculated the sum of all the individual species impact scores within each grid cell to give a total estimate of cumulative human impact. Spatial patterns of impact were strongly coherent between the two approaches, so we report on the more intuitive binary metric in the manuscript. We also ran a multiple linear regression on 10,000 randomly selected grid cells comparing the binary impact metric reported in the paper (a species and $\geq$1 threat = 1 impact in a grid cell) (response variable) and species richness and the mean human footprint in a grid cell as predictor variables. We obtained an R^2 value of 0.9, which shows that the human footprint and richness explain 90% of the variation in the model but also suggests that including species sensitivities to threats explains the other 10% of the variation. When we incorporate cumulative impacts (1 species + 3 threats = 3 impacts in a grid cell) and rerun the multiple linear regression, the R^2 drops to 0.77, suggesting that in areas where multiple threats are present, including species-specific threats is particularly important.

Mapping coolspots of threatened vertebrate anthropogenic refugia

We followed similar methods to mapping human impacts, where a cell was scored as an anthropogenic refuge if a species was present in the cell but no pressures that threaten it were present. These were then summed to give a cumulative score of the number of unimpacted species in a cell.

Supporting information

S1 Fig. Impact hotspots of individual human pressures on all threatened terrestrial vertebrates (*n* = 5,457), mammals (*n* = 1,277), birds (*n* = 2,120), and amphibians (*n* = 2,060). Scale represents the number of species impacted by the threat in a grid cell. Hotspots of impact are in dark red. Maps use a 30 km × 30 km grid and a Mollweide equal area projection. The data underlying this figure are freely available [31] (doi:10.1594/PANGAEA.897391).
(TIF)

S2 Fig. Impact hotspots of individual human pressures on all threatened terrestrial vertebrates (*n* = 5,457), mammals (*n* = 1,277), birds (*n* = 2,120), and amphibians (*n* = 2,060). Scale indicates the number of species impacted by the threat in a grid cell. Hotspots of impact are dark red. Maps use a 30 km × 30 km grid and a Mollweide equal area projection. The data underlying this figure are freely available [31] (doi:10.1594/PANGAEA.897391).
(TIF)

S3 Fig. Cumulative human impacts on all threatened terrestrial birds (*n* = 2,120), mammals (*n* = 1,277), and amphibians (*n* = 2,060). Scale indicates the number of species in a grid cell impacted by at least one threat. Areas of high human impact (hotspots) are red. Maps use a 30 km × 30 km grid and a Mollweide equal area projection. The data underlying this figure are freely available [31] (doi:10.1594/PANGAEA.897391).
(TIF)

S4 Fig. Threatened species richness for all taxa (*n* = 5,457), mammals (*n* = 1,277), birds (*n* = 2,120), and amphibians (*n* = 2,060). Areas of high human richness are red. Maps use a 30 km × 30 km grid and a Mollweide equal area projection. The data underlying this figure are freely available [31] (doi:10.1594/PANGAEA.897391).
(TIF)

S5 Fig. Coolspots of refugia for all threatened terrestrial mammals (*n* = 1,277), birds (*n* = 2,120), and amphibians (*n* = 2,060). Scale indicates the number of species not impacted by any threats in a grid cell. Coolspots of refugia are blue. Maps use a 30 km × 30 km grid and a Mollweide equal area projection. The data underlying this figure are freely available [31] (doi:10.1594/PANGAEA.897391).
(TIF)

S6 Fig. The percentage of species in a grid cell impacted by a threat (and inversely, the number of unimpacted species for whom it is a refuge) for (A) birds (*n* = 2,120), (B) mammals (*n* = 1,277), and (C) amphibians (*n* = 2,060). Maps use a 30 km × 30 km grid and a Mollweide equal area projection. The data underlying this figure are freely available [31] (doi:10.1594/PANGAEA.897391).
(TIF)

S1 Table. Major classes and subclasses of threats to biodiversity, as classified in the IUCN Red List of Threatened Species, the corresponding spatially explicit human pressure variable from the updated Human Footprint dataset and a brief description of how it was created, along with justifications for linking spatially explicit pressures to threats. IUCN, International Union for Conservation of Nature.
(DOCX)

S2 Table. The eight mapped human pressures, the number of sensitive species they impact, the area in which these impacts are occurring, and the proportion of Earth's terrestrial

area where these impacts are occurring.
(DOCX)

S3 Table. The top ten countries with the most impacted and unimpacted species on average.
(DOCX)

S4 Table. The average number of species impacted and unimpacted by threats per grid cell, and the proportion of species impacted by threats, in each of Earth's biomes.
(DOCX)

S5 Table. The average number of species impacted by threats per grid cell, and unimpacted by threats per grid cell, in each of Earth's ecoregions.
(DOCX)

S6 Table. Weights assigned to individual pressures in the Human Footprint and threshold scheme used to convert pressures into binary scores (present or absent) for impact analyses.
(DOCX)

S1 Data. Database containing information on the area and proportion of a threatened vertebrate species' range that is impacted by threats, including the data underpinning Fig 2.
(XLSX)

Acknowledgments

We would like to thank Kendall Jones and Sean Maxwell for comments on the manuscript and Ayesha Tulloch and Jonas Geldmann for productive discussions on the concept.

Author Contributions

Conceptualization: James R. Allan, James E. M. Watson, Moreno Di Marco, Hugh P. Possingham, Oscar Venter.

Formal analysis: James R. Allan, Moreno Di Marco, Christopher J. O'Bryan, Scott C. Atkinson.

Methodology: James R. Allan, James E. M. Watson, Moreno Di Marco, Oscar Venter.

Writing – original draft: James R. Allan, Christopher J. O'Bryan, Oscar Venter.

Writing – review & editing: James R. Allan, James E. M. Watson, Moreno Di Marco, Christopher J. O'Bryan, Hugh P. Possingham, Scott C. Atkinson, Oscar Venter.

References

1. Venter O, Sanderson EW, Magrach A, Allan JR, Beher J, Jones KR, et al. Sixteen years of change in the global terrestrial human footprint and implications for biodiversity conservation. Nature Communications. 2016; 7:12558. https://doi.org/10.1038/ncomms12558 PMID: 27552116
2. Newbold T, Hudson LN, Hill SLL, Contu S, Lysenko I, Senior RA, et al. Global effects of land use on local terrestrial biodiversity. Nature. 2015; 520:45. https://doi.org/10.1038/nature14324 PMID: 25832402
3. IUCN. The IUCN Red List of Threatened Species 2015 [cited on 9 December 2015]. http://www.iucnredlist.org.
4. Maxwell SL, Fuller RF, Brooks TM, Watson JEM. Biodiversity: The ravages of guns nets and bulldozers. Nature. 2016; 536:143–5. https://doi.org/10.1038/536143a PMID: 27510207

5. Jenkins CN, Pimm SL, Joppa LN. Global patterns of terrestrial vertebrate diversity and conservation. Proceedings of the National Academy of Sciences. 2013; 110(28):E2602–E10. https://doi.org/10.1073/pnas.1302251110 PMID: 23803854

6. Wilson KA, McBride MF, Bode M, Possingham HP. Prioritizing global conservation efforts. Nature. 2006; 440(7082):337–40. http://www.nature.com/nature/journal/v440/n7082/suppinfo/nature04366_S1.html. PMID: 16541073

7. Allan JD, McIntyre PB, Smith SDP, Halpern BS, Boyer GL, Buchsbaum A, et al. Joint analysis of stressors and ecosystem services to enhance restoration effectiveness. Proceedings of the National Academy of Sciences. 2013; 110(1):372–7. https://doi.org/10.1073/pnas.1213841110 PMID: 23248308

8. Hoffmann M, Hilton-Taylor C, Angulo A, Böhm M, Brooks TM, Butchart SHM, et al. The Impact of Conservation on the Status of the World's Vertebrates. Science. 2010; 330(6010):1503–9. https://doi.org/10.1126/science.1194442 PMID: 20978281

9. Waldron A, Miller DC, Redding D, Mooers A, Kuhn TS, Nibbelink N, et al. Reductions in global biodiversity loss predicted from conservation spending. Nature. 2017; 551:364–367. https://www.nature.com/articles/nature24295#supplementary-information. PMID: 29072294

10. Watson JEM, Evans T, Venter O, Williams B, Tulloch A, Stewart C, et al. The exceptional value of intact forest ecosystems. Nature Ecology & Evolution. 2018; 2(4):599–610. https://doi.org/10.1038/s41559-018-0490-x PMID: 29483681

11. Watson JEM, Venter O, lee J, Jones KR, Robinson JG, Possingham HP, et al. Protect the last of the wild. Nature. 2018; 536:27–30.

12. Halpern BS, Walbridge S, Selkoe KA, Kappel CV, Micheli F, D'Agrosa C, et al. A Global Map of Human Impact on Marine Ecosystems. Science. 2008; 319(5865):948–52. https://doi.org/10.1126/science.1149345 PMID: 18276889

13. Halpern BS, Kappel CV, Selkoe KA, Micheli F, Ebert CM, Kontgis C, et al. Mapping cumulative human impacts to California Current marine ecosystems. Conservation Letters. 2009; 2(3):138–48. https://doi.org/10.1111/j.1755-263X.2009.00058.x

14. Woolmer G, Trombulak SC, Ray JC, Doran PJ, Anderson MG, Baldwin RF, et al. Rescaling the Human Footprint: A tool for conservation planning at an ecoregional scale. Landscape and Urban Planning. 2008; 87(1):42–53. http://dx.doi.org/10.1016/j.landurbplan.2008.04.005.

15. Geldmann J, Joppa LN, Burgess ND. Mapping Change in Human Pressure Globally on Land and within Protected Areas. Conservation Biology. 2014; 28(6):1604–16. https://doi.org/10.1111/cobi.12332 PMID: 25052712

16. Sanderson EW, Jaith M, Levy MA, Redford KH, Wannebo AV, Woolmer G. The human footprint and the last of the wild. BioScience. 2002; 52(10):891–903.

17. Vorosmarty CJ, McIntyre PB, Gessner MO, Dudgeon D, Prusevich A, Green P, et al. Global threats to human water security and river biodiversity. Nature. 2010; 467(7315):555–561. https://doi.org/10.1038/nature09440 PMID: 20882010.

18. Ramírez F, Afán I, Davis LS, Chiaradia A. Climate impacts on global hot spots of marine biodiversity. Science Advances. 2017; 3(2):e1601198. https://doi.org/10.1126/sciadv.1601198 PMID: 28261659

19. Martins JH, Camanho AS, Gaspar MB. A review of the application of driving forces–Pressure–State–Impact–Response framework to fisheries management. Ocean & Coastal Management. 2012; 69:273–281. https://doi.org/10.1016/j.ocecoaman.2012.07.029

20. Evans MC, Watson JEM, Fuller RA, Venter O, Bennett SC, Marsack PR, et al. The Spatial Distribution of Threats to Species in Australia. BioScience. 2011; 61(4):281–289. https://doi.org/10.1525/bio.2011.61.4.8

21. Halpern BS, Frazier M, Potapenko J, Casey KS, Koenig K, Longo C, et al. Spatial and temporal changes in cumulative human impacts on the world's ocean. Nat Commun. 2015; 6:7615. https://doi.org/10.1038/ncomms8615 PMID: 26172980

22. Bellard C, Leclerc C, Courchamp F. Combined impacts of global changes on biodiversity across the USA. 2015; 5:11828. https://www.nature.com/articles/srep11828#supplementary-information. PMID: 26149694

23. Maxwell SM, Hazen EL, Bograd SJ, Halpern BS, Breed GA, Nickel B, et al. Cumulative human impacts on marine predators. Nature Communications. 2013; 4:2688. http://www.nature.com/articles/ncomms3688#supplementary-information. PMID: 24162104

24. Shackelford N, Standish RJ, Ripple W, Starzomski BM. Threats to biodiversity from cumulative human impacts in one of North America's last wildlife frontiers. Conservation Biology. 2018; 32(3):672–684. https://doi.org/10.1111/cobi.13036 PMID: 29068083

25. Schipper J, Chanson JS, Chiozza F, Cox NA, Hoffmann M, Katariya V, et al. The Status of the World's Land and Marine Mammals: Diversity, Threat, and Knowledge. Science. 2008; 322(5899):225–230. https://doi.org/10.1126/science.1165115 PMID: 18845749

26. Moran D, Kanemoto K. Identifying species threat hotspots from global supply chains. Nature Ecology & Evolution. 2017; 1:0023. http://www.nature.com/articles/s41559-016-0023#supplementary-information. PMID: 28812571

27. Joppa LN, O'Connor B, Visconti P, Smith C, Geldmann J, Hoffmann M, et al. Filling in biodiversity threat gaps. Science. 2016; 352(6284):416–418. https://doi.org/10.1126/science.aaf3565 PMID: 27102469

28. Tulloch VJD, Tulloch AIT, Visconti P, Halpern BS, Watson JEM, Evans MC, et al. Why do we map threats? Linking threat mapping with actions to make better conservation decisions. Frontiers in Ecology and the Environment. 2015; 13(2):91–99. https://doi.org/10.1890/140022

29. McGowan PJK. Conservation: Mapping the terrestrial human footprint. Nature. 2016; 537(7619):172–173. https://doi.org/10.1038/537172a PMID: 27604944

30. IUCN. Threats Classification Scheme Downloaded on 12 December 2015 [cited Version 3.2 on 12 December 2015]. Available from: http://www.iucnredlist.org/technical-documents/classification-schemes/threats-classification-scheme.

31. Allan JR, Watson JEM, Di Marco M, O'Bryan CJ, Possingham HP, Atkinson SC, et al. Supporting data for hotspots of human impact on threatened terrestrial vertebrates. PANGAEA. 2019.

32. Sodhi NS, Koh LP, Brook BW, Ng PKL. Southeast Asian biodiversity: an impending disaster. Trends in Ecology & Evolution. 2004; 19(12):654–660. https://doi.org/10.1016/j.tree.2004.09.006.

33. Olson DN, Dinerstein E, Wikramanayake ED, Burgess N, Powell GV, Underwood EC, et al. Terrestrial ecoregions of the world: a new map of life on Earth. BioScience. 2001; 51(11):933–938.

34. Gibson L, Lee TM, Koh LP, Brook BW, Gardner TA, Barlow J, et al. Primary forests are irreplaceable for sustaining tropical biodiversity. Nature. 2011; 478(7369):378–381. https://doi.org/10.1038/nature10425 PMID: 21918513

35. Maron M, Bowen M, Fuller RA, Smith GC, Eyre TJ, Mathieson M, et al. Spurious thresholds in the relationship between species richness and vegetation cover. Global Ecology and Biogeography. 2012; 21 (6):682–692. https://doi.org/10.1111/j.1466-8238.2011.00706.x

36. Ceballos G, Ehrlich PR, Dirzo R. Biological annihilation via the ongoing sixth mass extinction signaled by vertebrate population losses and declines. Proceedings of the National Academy of Sciences. 2017; 114(30):E6089–E96. https://doi.org/10.1073/pnas.1704949114 PMID: 28696295

37. Tucker MA, Böhning-Gaese K, Fagan WF, Fryxell JM, Van Moorter B, Alberts SC, et al. Moving in the Anthropocene: Global reductions in terrestrial mammalian movements. Science. 2018; 359(6374):466–469. https://doi.org/10.1126/science.aam9712 PMID: 29371471

38. Crooks KR, Burdett CL, Theobald DM, King SRB, Di Marco M, Rondinini C, et al. Quantification of habitat fragmentation reveals extinction risk in terrestrial mammals. Proceedings of the National Academy of Sciences. 2017; 114(29):7635–7640. https://doi.org/10.1073/pnas.1705769114 PMID: 28673992

39. Ceaușu S, Hofmann M, Navarro LM, Carver S, Verburg PH, Pereira HM. Mapping opportunities and challenges for rewilding in Europe. Conservation Biology. 2015; 29(4):1017–1027. https://doi.org/10.1111/cobi.12533 PMID: 25997361

40. Myers N, Mittermeier RA, Mittermeier CG, da Fonseca GAB, Kent J. Biodiversity hotspots for conservation priorities. Nature. 2000; 403(6772):853–858. http://www.nature.com/nature/journal/v403/n6772/suppinfo/403853a0_S1.html. PMID: 10706275

41. Brooks TM, Mittermeier RA, da Fonseca GAB, Gerlach J, Hoffmann M, Lamoreux JF, et al. Global biodiversity conservation priorities. Science. 2006; 313(5783):58–61. ISI:000238850200027. https://doi.org/10.1126/science.1127609 PMID: 16825561

42. Betts MG, Wolf C, Ripple WJ, Phalan B, Millers KA, Duarte A, et al. Global forest loss disproportionately erodes biodiversity in intact landscapes. Nature. 2017; 547:441. https://doi.org/10.1038/nature23285 PMID: 28723892

43. Scheffers BR, De Meester L, Bridge TCL, Hoffmann AA, Pandolfi JM, Corlett RT, et al. The broad footprint of climate change from genes to biomes to people. Science. 2016; 354(6313): aaf7671. https://doi.org/10.1126/science.aaf7671 PMID: 27846577

44. Martin TG, Watson JEM. Intact ecosystems provide best defence against climate change. Nature Clim Change. 2016; 6(2):122–124.

45. Margules CR, Pressey RL. Systematic conservation planning. Nature. 2000; 405(6783):243–253. https://doi.org/10.1038/35012251 PMID: 10821285

46. Venter O, Magrach A, Outram N, Klein CJ, Possingham HP, Di Marco M, et al. Bias in protected-area location and its effects on long-term aspirations of biodiversity conventions. Conservation Biology. 2018; 32(1):127–134. Epub 2017 Nov 16. https://doi.org/10.1111/cobi.12970 PMID: 28639356

47. Balmford A, Gaston KJ, Blyth S, James A, Kapos V. Global variation in terrestrial conservation costs, conservation benefits, and unmet conservation needs. Proceedings of the National Academy of Sciences. 2003; 100(3):1046–1050.

48. Tulloch AIT, Maloney RF, Joseph LN, Bennett JR, Di Fonzo MMI, Probert WJM, et al. Effect of risk aversion on prioritizing conservation projects. Conservation Biology. 2015; 29(2):513–524. https://doi.org/10.1111/cobi.12386 PMID: 25327837

49. Kareiva P, Marvier M. Conserving Biodiversity Coldspots: Recent calls to direct conservation funding to the world's biodiversity hotspots may be bad investment advice. American Scientist. 2003; 91(4):344–351.

50. Pressey RL, Weeks R, Gurney GG. From displacement activities to evidence-informed decisions in conservation. Biological Conservation. 2017; 212:337–348. https://doi.org/10.1016/j.biocon.2017.06.009.

51. Hoekstra JM, Boucher TM, Ricketts TH, Roberts C. Confronting a biome crisis: global disparities of habitat loss and protection. Ecol Lett. 2005; 8(1):23–29. https://doi.org/10.1111/j.1461-0248.2004.00686.x

52. Di Marco M, Butchart SHM, Visconti P, Buchanan GM, Ficetola GF, Rondinini C. Synergies and trade-offs in achieving global biodiversity targets. Conservation Biology. 2016; 30(1):189–95. https://doi.org/10.1111/cobi.12559 PMID: 26041135

53. Singh GG, Cisneros-Montemayor AM, Swartz W, Cheung W, Guy JA, Kenny T-A, et al. A rapid assessment of co-benefits and trade-offs among Sustainable Development Goals. Marine Policy. 2018; 93:223–231. Epub 2017 June 5. https://doi.org/10.1016/j.marpol.2017.05.030.

54. Ibisch PL, Hoffmann MT, Kreft S, Pe'er G, Kati V, Biber-Freudenberger L, et al. A global map of roadless areas and their conservation status. Science. 2016; 354(6318):1423–1427. https://doi.org/10.1126/science.aaf7166 PMID: 27980208

55. Wadey J, Beyer HL, Saaban S, Othman N, Leimgruber P, Campos-Arceiz A. Why did the elephant cross the road? The complex response of wild elephants to a major road in Peninsular Malaysia. Biological Conservation. 2018; 218:91–98. https://doi.org/10.1016/j.biocon.2017.11.036.

56. Laurance WF, Goosem M, Laurance SG. Impacts of roads and linear clearings on tropical forests. Trends Ecol Evol. 2009; 24(12):659–669. https://doi.org/10.1016/j.tree.2009.06.009 PMID: 19748151.

57. Stuart SN, Chanson JS, Cox NA, Young BE, Rodrigues ASL, Fischman DL, et al. Status and Trends of Amphibian Declines and Extinctions Worldwide. Science. 2004; 306(5702):1783–1786. https://doi.org/10.1126/science.1103538 PMID: 15486254

58. Ripple WJ, Abernethy K, Betts MG, Chapron G, Dirzo R, Galetti M, et al. Bushmeat hunting and extinction risk to the world's mammals. Royal Society Open Science. 2016; 3(10): 160498. https://doi.org/10.1098/rsos.160498 PMID: 27853564

59. Brook BW, Sodhi NS, Bradshaw CJA. Synergies among extinction drivers under global change. Trends in Ecology & Evolution. 2008; 23(8):453–460. https://doi.org/10.1016/j.tree.2008.03.011.

60. Mantyka-Pringle CS, Visconti P, Di Marco M, Martin TG, Rondinini C, Rhodes JR. Climate change modifies risk of global biodiversity loss due to land-cover change. Biological Conservation. 2015; 187:103–111. https://doi.org/10.1016/j.biocon.2015.04.016.

61. Di Marco M, Chapman S, Althor G, Kearney S, Besancon C, Butt N, et al. Changing trends and persisting biases in three decades of conservation science. Global Ecology and Conservation. 2017; 10:32–42. https://doi.org/10.1016/j.gecco.2017.01.008.

62. Donaldson MR, Burnett NJ, Braun DC, Suski CD, Hinch SG, Cooke SJ, et al. Taxonomic bias and international biodiversity conservation research. FACETS. 2016; 1(1):105–113. https://doi.org/10.1139/facets-2016-0011

63. NatureServe BIa. Bird Species Distribution Maps of the World. In: BirdLife International, editor. 2015.

64. Brooks TM, Akçakaya HR, Burgess ND, Butchart SHM, Hilton-Taylor C, Hoffmann M, et al. Analysing biodiversity and conservation knowledge products to support regional environmental assessments. Scientific Data. 2016; 3:160007. https://doi.org/10.1038/sdata.2016.7 PMID: 26881749

65. Rodrigues ASL, Pilgrim JD, Lamoreux JF, Hoffmann M, Brooks TM. The value of the IUCN Red List for conservation. Trends in Ecology & Evolution. 2006; 21(2):71–76. https://doi.org/10.1016/j.tree.2005.10.010.

66. Butchart SHM, Clarke M, Smith RJ, Sykes RE, Scharlemann JPW, Harfoot M, et al. Shortfalls and Solutions for Meeting National and Global Conservation Area Targets. Conservation Letters. 2015; 8(5):329–337. https://doi.org/10.1111/conl.12158

67. Seddon PJ, Griffiths CJ, Soorae PS, Armstrong DP. Reversing defaunation: Restoring species in a changing world. Science. 2014; 345(6195):406–412. https://doi.org/10.1126/science.1251818 PMID: 25061203

68. Venter O, Sanderson EW, Magrach A, Allan JR, Beher J, Jones KR, et al. Global terrestrial Human Footprint maps for 1993 and 2009. Scientific Data. 2016; 3:160067. https://doi.org/10.1038/sdata.2016.67 PMID: 27552448

69. Allan JR, Venter O, Watson JEM. Temporally inter-comparable maps of terrestrial wilderness and the Last of the Wild. Scientific Data. 2017; 4:170187. https://doi.org/10.1038/sdata.2017.187 PMID: 29231923

70. Laurance, Sloan S, Weng L, Sayer J. Estimating the Environmental Costs of Africa's Massive Development Corridors Current Biology. 2015; 25(24):3202–3208. https://doi.org/10.1016/j.cub.2015.10.046 PMID: 26628009

71. Hulme PE. Trade, transport and trouble: managing invasive species pathways in an era of globalization. Journal of Applied Ecology. 2009; 46(1):10–18. https://doi.org/10.1111/j.1365-2664.2008.01600.x

72. Meunier G, Lavoie C. Roads as Corridors for Invasive Plant Species: New Evidence from Smooth Bedstraw (Galium mollugo). Invasive Plant Science and Management. 2012; 5(1):92–100. https://doi.org/10.1614/IPSM-D-11-00049.1

73. Di Marco M, Watson JEM, Possingham HP, Venter O. Limitations and trade-offs in the use of species distribution maps for protected area planning. Journal of Applied Ecology. 2017; 54(2):402–411. Epub 2016 August 13. https://doi.org/10.1111/1365-2664.12771
